# Synthesis and Structure of Novel Phenothiazine Derivatives, and Compound Prioritization via In Silico Target Search and Screening for Cytotoxic and Cholinesterase Modulatory Activities in Liver Cancer Cells and In Vivo in Zebrafish

**DOI:** 10.1021/acsomega.3c06532

**Published:** 2024-07-03

**Authors:** Mehmet Murat Kisla, Murat Yaman, Fikriye Zengin-Karadayi, Busra Korkmaz, Omer Bayazeid, Amrish Kumar, Ravindra Peravali, Damla Gunes, Rafed Said Tiryaki, Emine Gelinci, Gulcin Cakan-Akdogan, Zeynep Ates-Alagoz, Ozlen Konu

**Affiliations:** † Department of Pharmaceutical Chemistry, Faculty of Pharmacy, Ankara University, 06100 Ankara, Turkey; ‡ Graduate School of Health Sciences, Ankara University, 06100 Ankara, Turkey; § Interdisciplinary Program in Neuroscience, Bilkent University, 06800 Ankara, Turkey; ∥ Department of Molecular Biology and Genetics, Bilkent University, 06800 Ankara, Turkey; ⊥ Institute of Toxicology and Genetics (ITG), Karlsruhe Institute of Technology (KIT), 76344 Eggenstein-Leopoldshafen, Germany; # Izmir Biomedicine and Genome Center (IBG), 35340 Izmir, Turkey; ¶ Medical Biology Department, Dokuz Eylul University, 35340 Izmir, Turkey; ∇ UNAM-Institute of Materials Science and Nanotechnology, Bilkent University, 06800 Ankara, Turkey

## Abstract

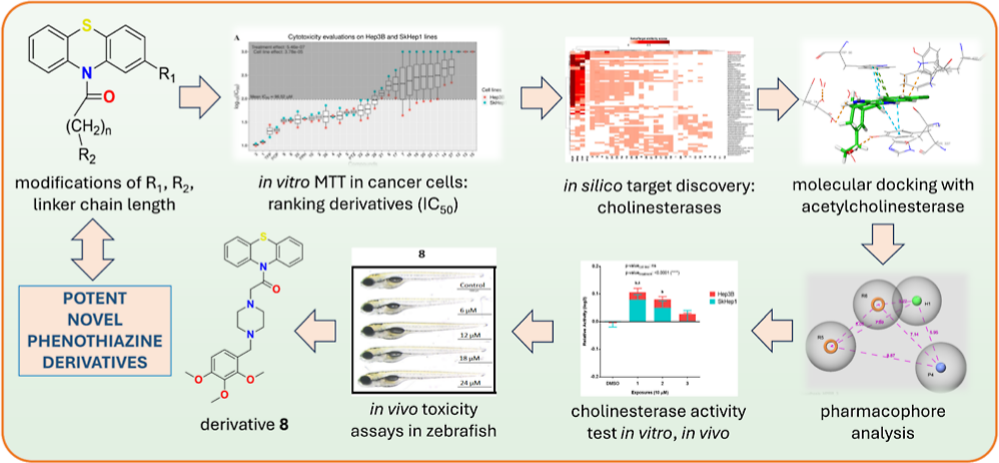

Phenothiazines (PTZ) are antipsychotics known to modulate a variety of neurotransmitter activities that include dopaminergic and cholinergic signaling and have been identified as potential anticancer agents in vitro. However, it is important to also test whether a highly cytotoxic, repurposed, or novel PTZ has low toxicity and neuromodulatory activity in vivo using vertebrate model organisms, such as zebrafish. In this study, we synthesized novel phenothiazines and screened them in vitro in liver cancer and in vivo in zebrafish embryos/larvae. The syntheses of several intermediate PTZ 10-yl acyl chlorides were followed by elemental analysis and determination of ^1^H NMR and ^13^C NMR mass (ESI^+^) spectra of a large number of novel PTZ 10-carboxamides. Cytotoxicities of 28 PTZ derivatives **(1–28)** screened against Hep3B and SkHep1 liver cancer cell lines revealed five intermediate and five novel leads along with trifluoperazine (TFP), prochlorperazine (PCP), and perphenazine, which are relatively more cytotoxic than the basic PTZ core. Overall, the derivatives were more cytotoxic to Hep3B than SkHep1 cells. Moreover, in silico target screening identified cholinesterases as some of the commonest targets of the screened phenothiazines. Interestingly, molecular docking studies with acetylcholinesterase (AChE) and butyrylcholinesterase proteins showed that the most cytotoxic compounds **1**, **3**, PCP, and TFP behaved similar to Huprin W in their amino acid interactions with the AChE protein. The highly cytotoxic intermediate PTZ derivative **1** exhibited a relatively lower toxicity profile than those of **2** and **3** during the zebrafish development. It also modulated in vivo the cholinesterase activity in a dose-dependent manner while significantly increasing the total cholinesterase activity and/or *ACHE* mRNA levels, independent of the liver cancer cell type. Our screen also identified novel phenothiazines, i.e., **8** and **10**, with significant cytotoxic and cholinesterase modulatory effects in liver cancer cells; yet both compounds had low levels of toxicity in zebrafish. Moreover, they modulated the cholinesterase activity or expression of *ACHE* in a cancer cell line-specific manner, and compound **10** significantly inhibited the cholinesterase activity in zebrafish. Accordingly, using a successful combination of in silico, in vitro, and in vivo approaches, we identified several lead anticancer and cholinesterase modulatory PTZ derivatives for future research.

## 1 Introduction

Drug repurposing is the practice of utilizing clinically tested drugs in the market for alternative pathologies.^1^ Previous findings and published reviews have demonstrated the highly antiproliferative effects and therapeutic potential of antipsychotic phenothiazine (PTZ)-based drugs that act via different mechanisms including modulation of autophagy, membrane disruption/permeabilization, efflux pump inhibition, calcium overload, and/or other cell signaling pathways.^2−14^ For instance, thioridazine selectively targets leukemia cancer stem cells of metastatic nature^15^ while halting cell cycle at *G*_0_/*G*_1_ phase and prevents the migration of tumor cells.^16^ Haloperidol, fluphenazine, and flupentixol induce dose-dependent cell death in neuroblastoma and glioma cell lines^17,18^ while perphenazine (PPH) has been shown to modulate negatively the cell cycle of SH-SY5Y neuroblastoma cell line.^18^ Additionally, the combined therapy of chlorpromazine and tamoxifene has led to synergistic anticancer effects.^19^ In addition, the dose-dependent DNA fragmentation in glioma and neuroblastoma cell lines by fluphenazine, thioridazine, and PPH^20^ and enhanced apoptosis of B16 melanoma cells, triggered by thioridazine,^21^ have also been demonstrated.

Liver has been another tissue targeted by phenothiazines. For example, fluphenazine exhibits hepatocellular effects^12,14^ while chlorpromazine has emerged to reduce the hepatotoxic effects of acetaminophen.^13^ Moreover, pathways, such as MAP kinase, Wnt, and retinoic acid signaling, also known to be involved in liver tumorigenesis, have been identified as targets of phenothiazines.^22^ Although liver cancer treatments include the use of kinase inhibitors, like sorafenib (SFB) and lenvatinib^23^ recently, trifluoperazine (TFP) and chlorpromazine have also been repurposed with anticancer activity against liver cancer cell lines in a high-throughput study.^24^ Therefore, there is a continuing need to synthesize and test novel phenothiazines for their promising cytotoxic effects in vitro by using liver cancer cell lines.

Although PTZ derivatives are well-known to interact with dopaminergic, serotoninergic, histaminergic, and muscarinic receptors, they also can modulate cholinesterase activity.^25^ Acetylcholine (ACh) levels are modulated upon hydrolysis by acetylcholinesterase (AChE) and butyrylcholinesterase (BChE) enzymes,^26,27^ which interact with different proteins,^28^ and can modulate cell proliferation and spheroid formation in hepatocellular carcinoma (HCC) via different mechanisms that might include loss of AChE activity.^29^ Moreover, in the HCC cell lines Huh-7, and HepG2, AChE activity might decrease with respect to increasing *ACHE* protein levels, suggesting presence of a feedback and/or post-translational dysregulation; yet this needs further support. In addition, the tumor suppressor-like effects of AChE activity has been stated as a potential prognostic marker in HCC^30^ while the cholinesterase levels in the serum have indeed predicted the efficacy of SFB therapy for HCC in clinic.^31,32^ All of these findings suggest that liver cancer cell lines could be used effectively to test cytotoxic and cholinergic effects of novel and known phenothiazines. However, in silico target discovery analyses of novel and known phenothiazines are also needed to better assess the potential interactions between phenothiazines and cholinesterases.

**Figure 1:**
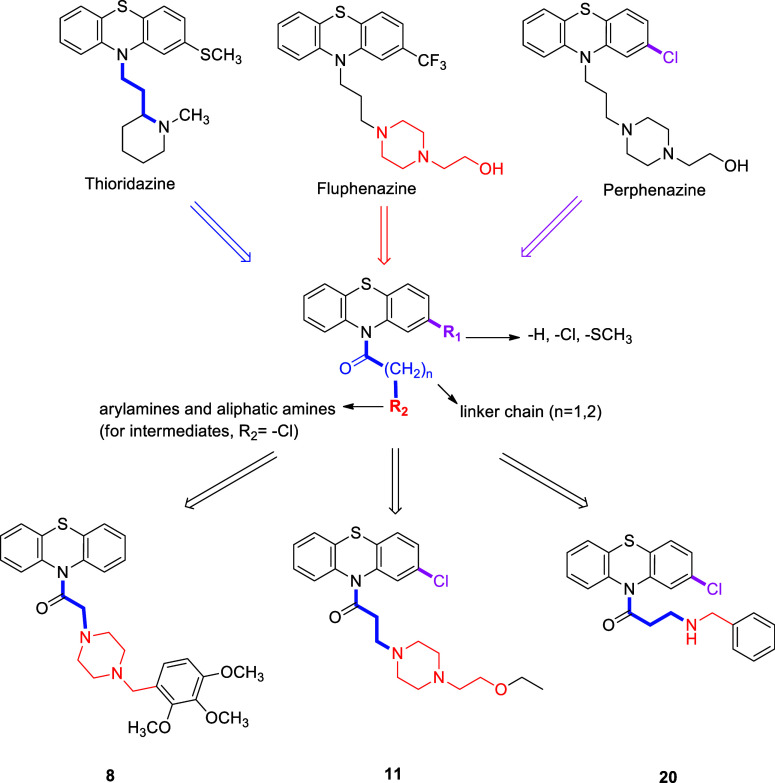
Design strategy for PTZ derivatives based on the structures of cytotoxic phenothiazines.

Interestingly, different PTZ structures demonstrate selectivity toward modulating cholinesterase activity in both derivative- and/or concentration-dependent manners.^25,33,34^ For instance, amine and methylamine-substituted PTZ derivatives have ACh modulatory effects.^35^ Fluphenazine can block ACh receptor-operated potassium current that is induced by carbachol.^36^ Ashoor et al. (2011) have discovered that fluphenazine could also inhibit the CHRNA7 ligand binding at a concentration of 10 μM.^37^ Therefore, it could be important to synthesize and discover novel phenothiazines that can modulate cholinesterase activity and, at the same time, exhibit cytotoxic effects in liver cancers. In this study, we have implemented a design strategy starting with the PTZ derivatives with known anticancer effects, to obtain novel drug candidates with potential cholinergic and/or anticancer effects (Figure 1).

In addition, ACh synthesis and degradation has to be in balance because sudden inhibition of AChE leads to paralysis and death.^38^ Previous studies have established the presence of a feedback between AChE activity and *ACHE* transcription^39−41^ as well as other feedback mechanisms involving changes in the *BCHE* expression and activity of BChE^42−44^ and acetycholine receptors^40,45^ acting as modulators of cholinergic signaling.^46^ However, to our knowledge, there is no study in the literature testing the association between the enzyme activity and transcription levels of cholinesterases in response to the PTZ exposure in cancer cells.

Moreover, the regulation of ACh levels is evolutionarily conserved across species, allowing in vivo studies in model organisms^47−49^ with keeping in mind atypical enzymatic activities, e.g., by butyrylcholinesterases.^50,51^ Zebrafish (*Danio rerio*) is a highly suitable model for screening the cytotoxic effects of drugs during embryonic and larval stages.^52^ In addition, zebrafish has only *ache* but no *bche* expression/activity, making it an excellent model organism to decipher the role of changes in AChE activity on mortality rates and LC_50_ estimates in response to drugs.^53^ Several studies have demonstrated that phenothiazines can be effective cytotoxic agents in zebrafish inducing apoptosis,^54^ are used in models of tumor xenografts,^55^ and play roles in autophagy^56^ and antibacterial activity.^57^ In addition to the abovementioned toxicology and anticancer applications, several antipsychotics have already been tested in zebrafish for their effects on locomotion^58^ and/or photomotor activity using high throughput behavioral systems.^59−61^

The present study has been based on the synthesis of intermediate and novel PTZ derivatives with potential cytotoxic and cholinesterase modulatory activities.^25^ In vitro cytotoxicity together with molecular docking, other in silico analyses, and in vivo zebrafish assays have led us to identify multiple intermediate, novel, as well as known phenothiazines with potential cholinesterase modulatory activities and/or exhibiting significantly high cytotoxicity in cancer cells but low toxicity in zebrafish. Moreover, we demonstrated that several lead compounds also had significant cholinesterase modulatory activity at the level of enzyme activity and/or mRNA. Accordingly, the acquired data from this study are also likely to shed light on the criteria that influence the interactions of phenothiazines with modulation of cholinesterases, which has received relatively less attention in the literature.

## 2 Results and Discussion

### 2.1 Synthesis of the PTZ Derivatives

**Scheme 1:**
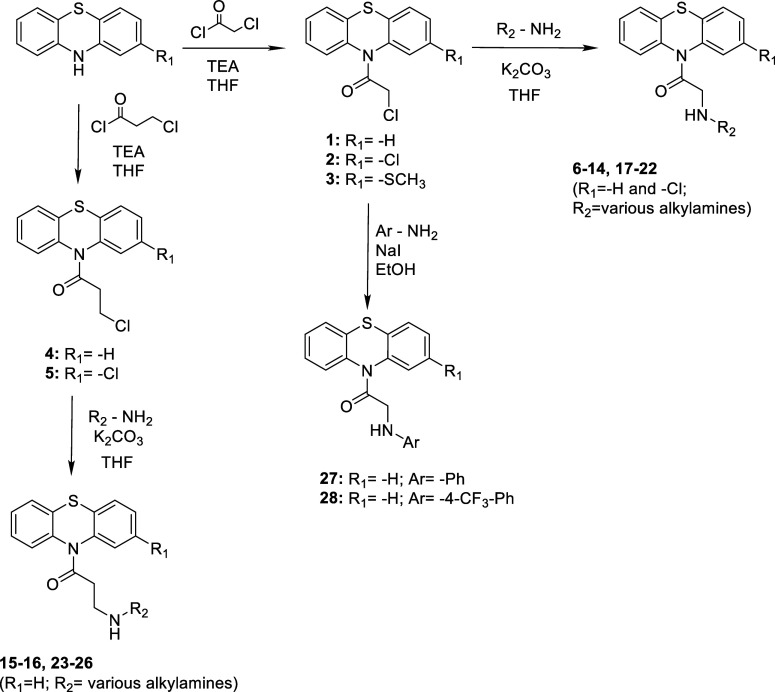
General Synthesis Procedure for the PTZ Derivatives **1-28**

As shown in Scheme 1, the synthesis of PTZ derivatives began with suitable phenothiazines. Compounds **1**–**5** were synthesized by combining acyl chlorides and tetrahydrofuran (THF). Afterward, the solution of produced intermediate was added dropwise to the alkylamine solution and heated under reflux until the starting material was consumed, yielding **6**–**26** (Scheme 1). For the synthesis of **27** and **28**, arylamines and NaI were added to a solution of **1** in EtOH at rt (Scheme 1).^62^ The mixture was then heated under reflux until the starting material had been consumed. Section 4.2 describes the full processes for the synthesis of **1**–**28**. For the final derivatives, –Cl was preserved in the PTZ ring; and using Darvesh et al.’s technique^25^ as a basis, 10-carbonyl derivatives of this ring that are expected to have cholinesterase activity were prepared. Simultaneously, the atomic distances between the PTZ and amine groups were fixed at 1 or 2 carbons in order to study how chain length affected the activity. Instead of the piperazine ring found in fluphenazine structures, aliphatic or aromatic ring systems derived from fluphenazine and thioridazine with –F, –Cl, and –CF_3_ were used. The structures of these derivatives were identified via instrumental analysis. The proton-decoupled ^13^C NMR spectra of compounds **17** and **25** revealed carbon–fluorine couplings. The interaction between the initial carbon and fluorine atoms had a frequency of 243 Hz and emerged around 160–163 ppm. The frequencies of the second, third, and fourth carbon and fluorine interactions were 21.2, 7.7–8.4, and 3–3.1 Hz, and they emerged at 115, 129, and 135 ppm. The quartet at 126.6 ppm (*q*,–CF_3_) observed in compound **28** can be attributed to the trifluoromethyl group.

### 2.2 Cytotoxicity of the PTZ Derivatives

**Figure 2:**
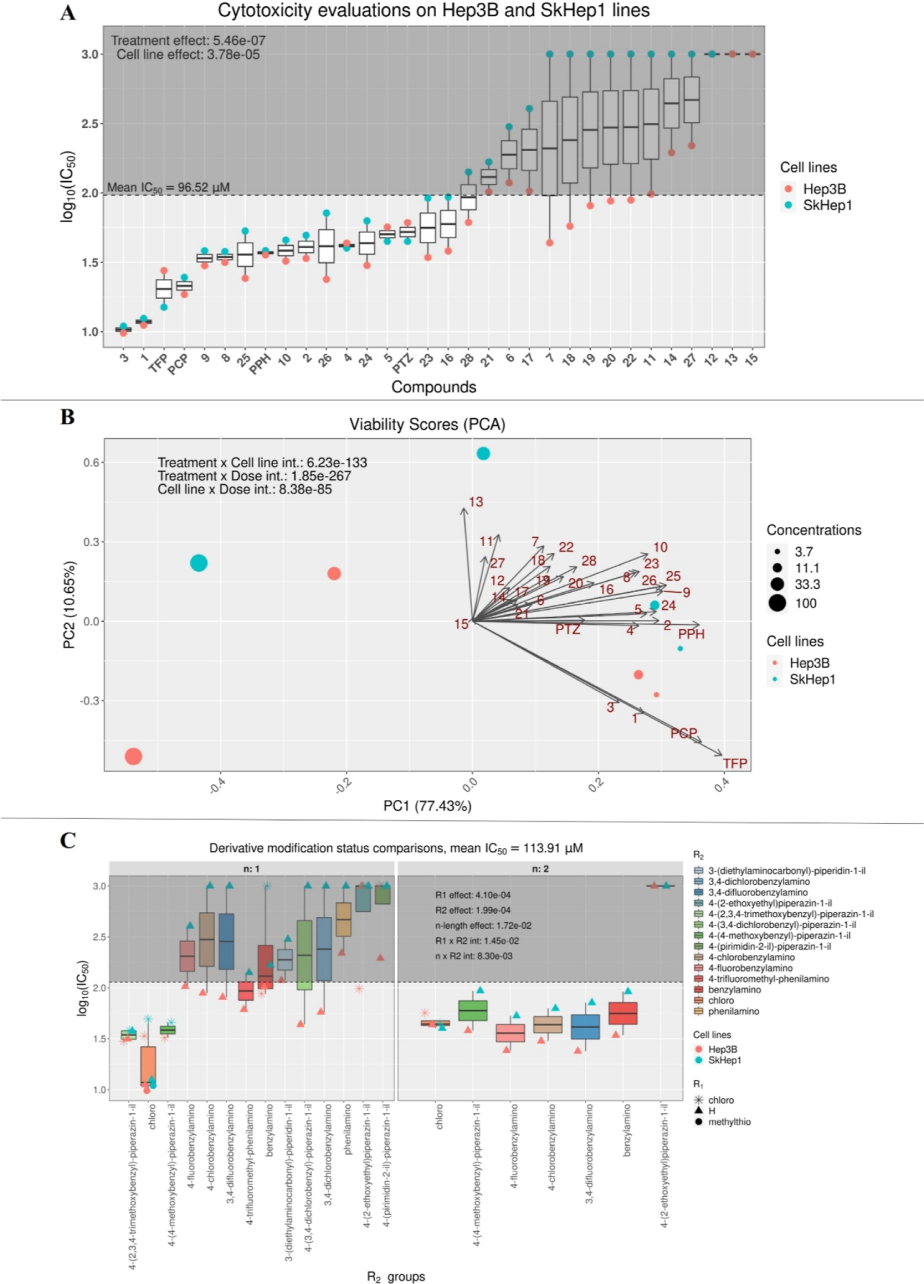
Changes in cell viability upon exposure to the derivatives in Hep3B and SkHep1 cells. (A) Known and novel derivatives’ IC_50_ values; (B) PCA on cell viabilities across different doses of drugs; and (C) side-chain modifications by the intermediary and novel derivatives and their influences on the IC_50_ levels. Significance levels (*p*-values) are derived from n-way ANOVA for each respective comparison in R environment.

We have evaluated the effects of four known [TFP, PCP, PPH, and PTZ] and **1**–**28** intermediate or novel compounds that we have synthesized on liver cancer cell viability by calculating the IC_50_ values in Hep3B and SkHep1 cell lines in vitro (Figure 2). We have seen that the derivatives yielded significantly different treatment effects (p-value: 5.46 × 10^–7^) in a cell-type-dependent manner (p-value: 3.78 × 10^–5^) (Figure 2A). Among the screened derivatives, intermediary compounds **1** and **3** stood out, having the highest cytotoxic effects. Commercial derivatives TFP, PCP, and PPH as well as the novel derivatives **8**, **9**, **10**, and **25** were also among the most cytotoxic compounds when both cell lines were examined. Interestingly, the original PTZ scaffold was relatively less toxic (Figure 2A). Nonetheless, SFB used as a control still remained as the most active compound with the IC_50_ values of 5.97 and 0.26 μM, for Hep3B and SkHep1, respectively. Moreover, Hep3B indicated a more sensitive profile than SkHep1 in response to the PTZ derivatives overall (*p*-value: 0.017; Figure S1).

First two principal components were able to explain close to 90% of the variability in the data where the most active compounds, **1**, **3**, TFP and PCP aligned together (Figure 2B). The remaining active derivatives (**2**, **4**, **5**, **8**, **9**, **10**, **24**, **25**, PPH, and PTZ) clustered across the first principal component. In addition to the concentration-dependent differences across the principal components, we have observed an interaction between cell-type dependence and concentration (*p*-value: 8.38 × 10^–85^).

Assessments on R_1_, R_2_, and *n*-length of the intermediary and novel derivatives showed statistically significant differences on the cytotoxicity levels (Figure 2C). Despite the limited range of R_1_ substitutions employed, R_2_-based comparisons demonstrated the significant effect of R_2_: -chloro additions, as in the case of the compounds **1** and **3**. Futhermore, the length of the linker chain (n-length) by the R_2_ side was significant (*p*-value: 1.72 × 10^–2^), yet differentially, suggesting a dependence on additional factors like R_1_ and R_2_ status. For instance, R_2_:-chloro moieties yielded relatively less cytotoxicity when the length of the linker chain increased from one to two. In contrast, R_2_:-4-fluorobenzylamino derivations followed an opposite trend with respect to the link length. Hence, the effect of R_2_ modifications can depend on the n-length (p-value: 8.30 × 10^–3^). In addition, significant interaction between R_1_ and R_2_ substitutions (*p*-value: 1.45 × 10^–2^) supported the notion that the effects of R_1_ and R_2_ were interdependent. This relationship was irrespective of the cell lines tested (*p*-values: R_1_ × cell line: 0.25; R_2_ × cell line: 0.078; and R_1_ × R_2_ × cell line: 0.092), underlying the primary importance of side-chain moieties on cytotoxicity profiles.

### 2.3 *In Silico* Target Screening with PTZ Derivatives

SwissTargetPrediction tool, based on 2D/3D similarities with a library of 280,381 small compounds with known interactions,^63^ revealed cholinesterases, and dopamine and serotonine receptors/transporters were among the top candidates with which the derivatives could interact (Figure S2). Among them, AChE and BChE were almost entirely common across the PTZ derivatives. Moreover, muscarinic ACh receptors were found to be mutual targets for the known and most of the intermediary derivatives, except compound **3**. On the one hand, the dopaminergic receptor D2 was found in a separate clade than the other dopaminergic receptors (Figure S2). In addition, the serotoninergic system members were shared among the active compounds PCP, TFP, **1**, **2**, and **10** while tyrosine protein kinases came up as potential targets for the abovementioned compounds along with **8** and **9**. Herein, we focused on in silico molecular docking studies and in vitro/in vivo cholinesterase activity assessments as the most common theme among the derivatives.

### 2.4 Molecular Docking for Cholinesterase Affinity Prediction

**Figure 3:**
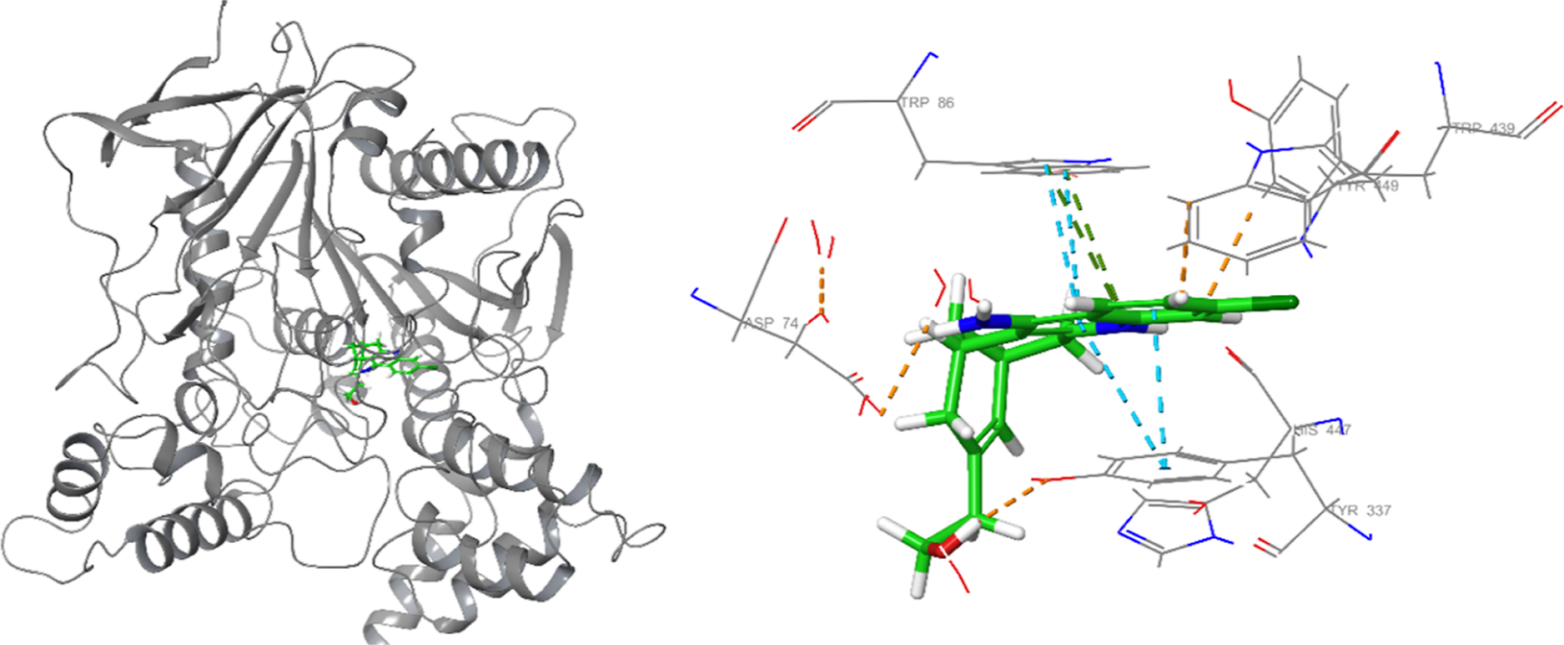
Binding mode of HUW with AChE. Several interactions with polar and hydrophobic residues were apparent.

**Figure 4:**
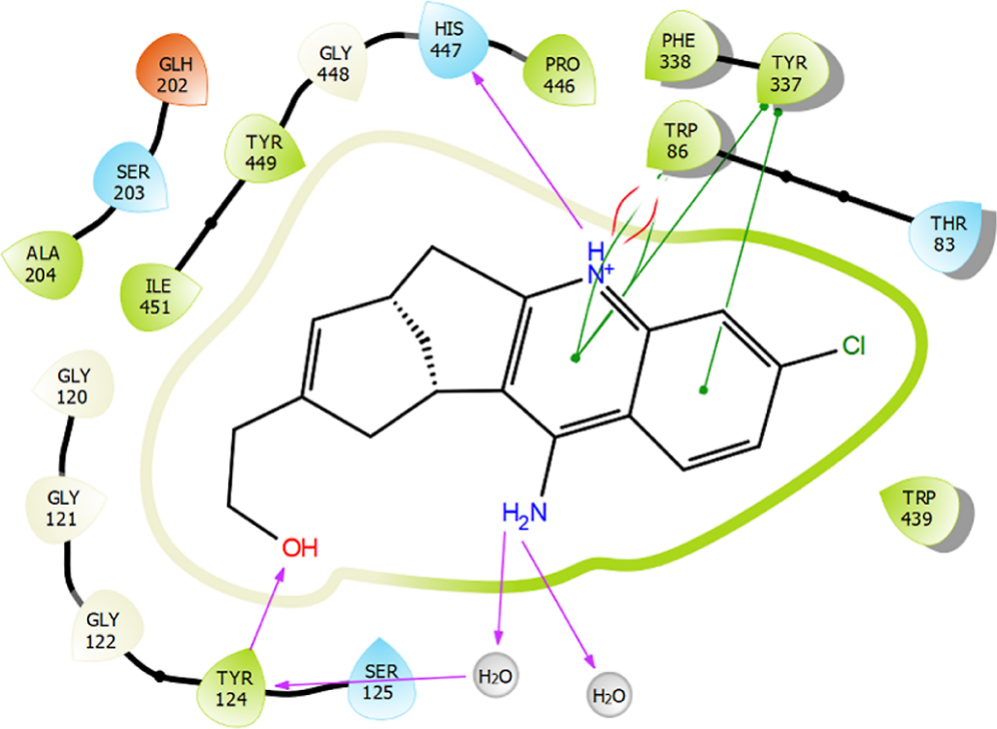
Interaction profile of HUW in the binding site (glide score = −14.76). Purple color represents H-bond interactions, whereas red lines define Pi–cation interactions. Green lines represent Pi–Pi interactions.

For investigating the cholinesterase-modulating activities of the PTZ derivatives, AChE enzyme (pdb id: 4BDT) was prioritized. First, coligand Huprine W (HUW) was extracted and redocked to this protein, and root-mean-square deviation (rmsd) value between the redocked and original pose was calculated. Interactions in this complex and the former studies^64,65^ were used as reference; thus, the phenothiazines mimicking these interactions were investigated. Binding modes and interactions are given in Figures 3 and 4 in detail.

According to the diagram in Figure 4, the positively charged nitrogen in the quinolinic moiety donated a proton to polar residue His447 and created a Pi–cation interaction with hydrophobic Trp86. Meanwhile, the primary amino group afforded indirect H-bond interactions with Tyr124 through water molecules. Another H-bond interaction with this residue took place through alcoholic side chain. On the other hand, the quinoline moiety got stacked with aromatic ring Tyr337.

**Figure 5:**
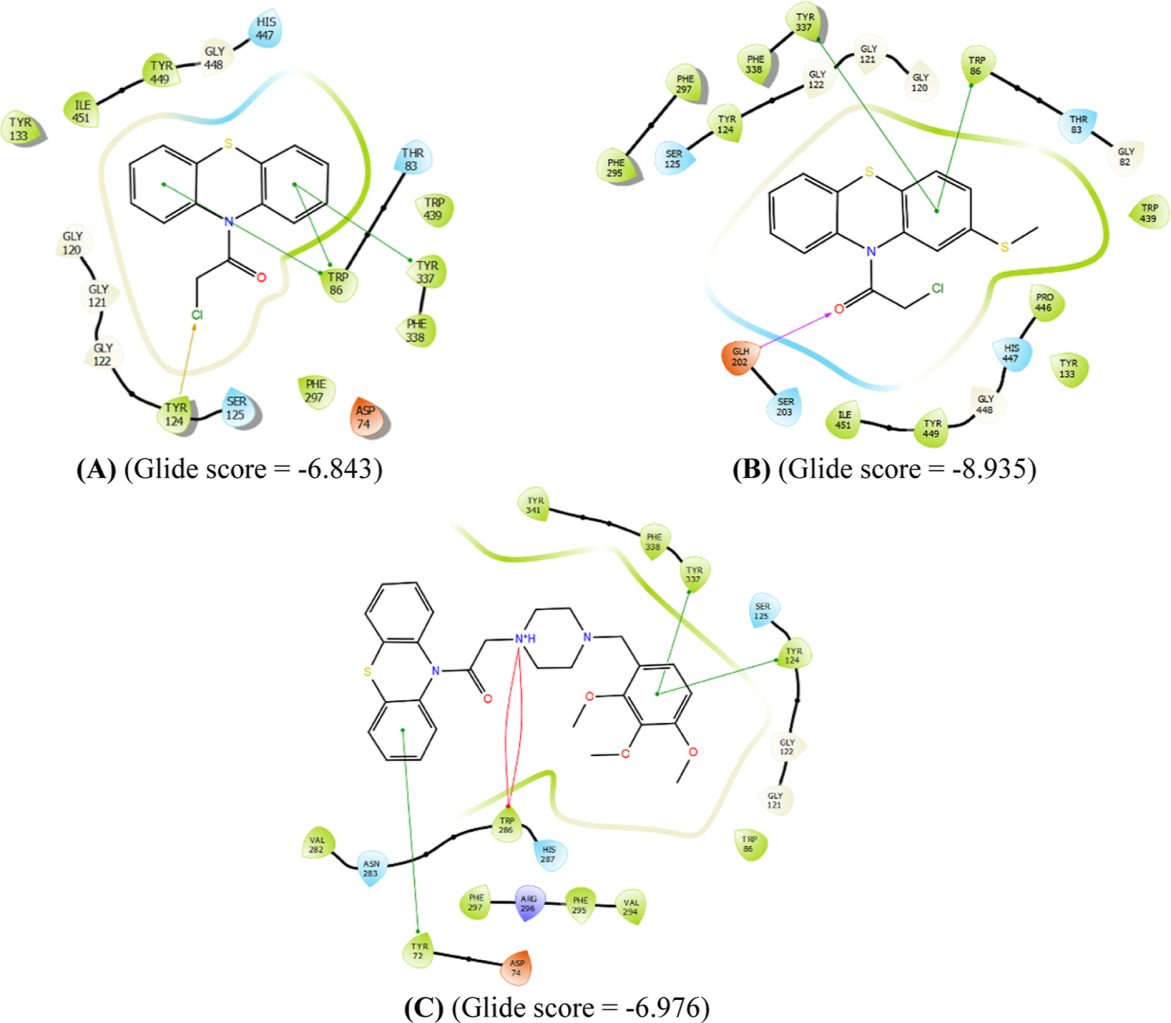
Interaction profiles of the most potent ligands **1** (A), **3** (B), and **8** (C) against HCC cell lines. Purple color represents H-bond interactions, whereas red elliptic line defines Pi-cation interaction. In addition, green lines represent Pi–Pi interactions, and gold arrow is for the halogen bond interaction.

Compound **1** exhibited the most similarity to **HUW** via interactions with Try337 and Trp86 as well as Try124 although the glide score indicated lower affinity (Figure 5A). On the other hand, compound **3** was elected as one of the most cytotoxic PTZ derivatives. Evidently in Figure 5B, this compound formed a similar Pi-cation interaction to that of **HUW** via its carbonyl group. Again, a Pi–Pi interaction has occurred with Tyr337 and Trp86, which would increase the stability of this compound in the binding site. For compound **8**, the PTZ phenyl creates steric interaction with Tyr72 as the phenyl of trimethoxylphenyl creates a steric interaction with Tyr124 and Tyr337. Protonated nitrogen in the piperazinic moiety offered a cationic interaction with Trp286 (Figure 5C). According to IC_50_ values, the most cytotoxic novel lead was chosen as **8**; and in docking studies (Figure 5C), this compound formed steric stacking interactions with hydrophobic residues Tyr337 and Trp86, similar to **HUW**. A surplus H-bond interaction was present between the acceptor carbonyl group and donor Glh202 residue. Relative to the Glide score of **HUW**, these derivatives offered lower affinity.

Glide scores for all the novel, intermediate, and commercial derivatives with AChE and BChE were calculated (Tables S1 and S2); and when compared with standards **HUW** and **Tacrine**, respectively for AChE and BChE, they were found abysmal for BChE (with most of them failing to bind), rendering modulation of AChE far more potent than that of BChE. Among the intermediate derivatives, compound **2** was not able to bind with either of them while **1** and **3** offered high affinities (Figure 5 and Table S1).

### 2.5 Physicochemical Characterization

Molecular descriptors were calculated via the QikProp module of Maestro to evaluate the drug likeness of synthesized PTZ derivatives (Table 1). Molecular weight is an informative value that should be below 725 according to QikProp manual; and all the compounds have suited this rule. Among the ligands, PTZ and **1** had low molecular volumes, which could influence their pharmacokinetic processes. Volume values were also desirable for binding with AChE since the cavity was rather small. Log P range of the QikProp manual was −2.0 to 7.5, and all the derivatives were within this range. Compounds **16**, **18**, and **20** have had extremely high log P values relative to those of **HUW**. This descriptor should be <3 to ensure less permeability through lipophilic barriers (such as blood–brain barrier for side effects) and less toxicity via accumulation in tissues and **HUW** suits to this. The derivatives had high human oral absorption values according to the manual. Unlike **HUW**, **10** and **13** have had 100% oral absorption values which could render them as suitable drug candidates (Table 1).

**Table 1:** Calculated QikProp Molecular Descriptors of HUW, PTZ, and PTZ Derivatives

article codes	molecular weight (g/mol)	molecular volume (Å^3^)	QP log *p*	% oral absorption
PTZ	199.270	663.265	3.568	100.000
HUW	313.829	990.146	2.876	83.916
1	275.752	804.331	3.570	100.000
2	310.197	857.505	4.186	100.000
3	321.839	937.634	4.338	100.000
4	289.779	862.599	4.987	100.000
5	324.224	921.603	4.647	100.000
6	423.570	1296.380	3.610	100.000
7	484.440	1443.618	4.910	100.000
8	505.630	1465.745	3.830	100.000
9	431.979	1328.119	3.192	100.000
10	540.076	1562.860	4.538	86.190
11	437.946	1296.380	4.140	100.000
12	491.665	1478.191	4.406	100.000
13	397.534	1286.723	2.705	87.888
14	403.501	1251.178	3.624	100.000
15	411.561	1375.369	4.508	100.000
16	445.578	1393.128	6.164	100.000
17	364.436	1126.716	4.490	100.000
18	415.336	1199.044	5.237	100.000
19	382.427	1111.739	4.457	100.000
20	380.891	1162.276	4.810	100.000
21	346.446	1117.491	4.299	100.000
22	380.891	1161.551	4.793	100.000
23	360.473	1164.140	5.422	100.000
24	394.918	1207.711	5.912	100.000
27	332.419	1051.181	4.604	100.000
28	400.418	1119.542	5.364	100.000

### 2.6 Pharmacophore Analysis

Known and newly synthesized PTZ derivatives were screened using an authentic HPRR_3 pharmacophore hypothesis (see the Materials and Methods section). Among these derivatives: compounds **8**, **9**, and **10**, which were found to have favorable IC_50_ values, also possessed relatively higher fitness values (Table S3). However, **7** offered the worst fitness score. For **9** and **10**, the chlorine atom at the 2° position of the PTZ ring has increased the fitness to HPRR_3 that has a hydrophobic feature. This feature was absent for compounds **7** and **8**, resulting in abysmal fitness scores. All these hits had a piperazinic moiety with two protonable tertiary nitrogen atoms, which was also necessary for a favorable fitness value. Judging by the substituents of the benzylic regions, -mono and -trimethoxy substitution could be the main rationale for increasing the activity. However, hydrophobic substitution of this moiety resulted in the lowest fitness value. These results altogether created a foundation for a preliminary SAR analysis of these PTZ derivatives.

### 2.7 Modulation of Cholinesterase Activity by PTZ Derivatives

**Figure 6:**
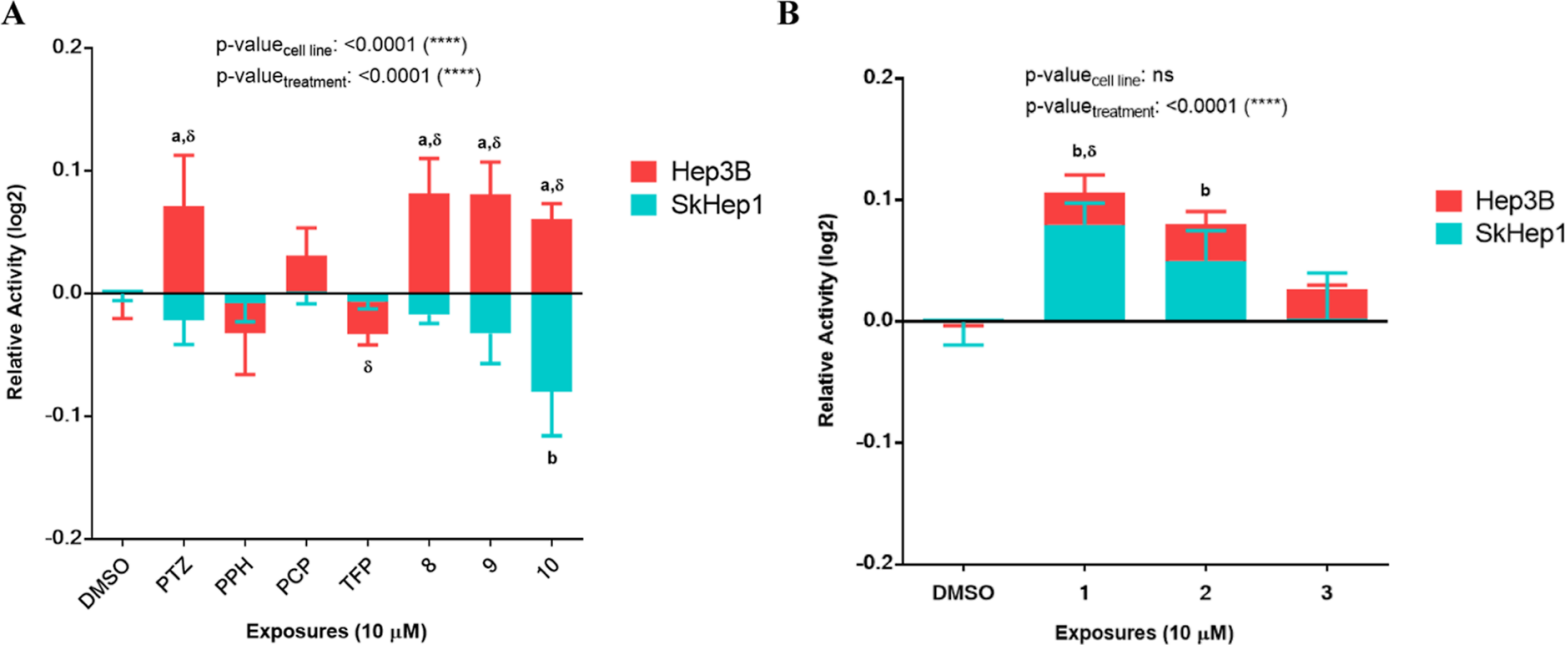
Cholinesterase activity level changes upon PTZ derivative exposures. (A) Hep3B and SkHep1 cholinesterase activity levels. (B) Cholinesterase activity levels after 24 h PTZ derivative exposures to SkHep1 and Hep3B cells. Two-way ANOVA/Dunnett’s comparisons test with respect to DMSO control (*p*-values: *a*,*b* ≤ 0.05) and multiple *t* tests/Holm-Sidak between the cell lines (*p*-value: δ ≤ 0.05) were applied as the statistical methods.

The derivatives’ cholinesterase activities were evaluated in vitro in Hep3B and SkHep1 cells (Figure 6); and the cholinesterase activity response to PTZ derivatives differed between Hep3B and SkHep1 cells, in which the latter had higher basal endogenous activity. We found that the cholinesterase activity of SkHep1 cells remained relatively stable in response to the tested drugs except compound **10**, which significantly reduced this activity (Figure 6A). On the other hand, in Hep3B, a cell line with endogenously low basal cholinesterase activity, several different derivatives, e.g., PTZ, **8**, **9**, and **10**, resulted in significant increases in the cholinergic activity. On the other hand, the exposure to derivatives **1**, **2**, or **3** showed a tendency to increase cholinesterase activity in both Hep3B and SkHep1 (Figure 6B). The effect of compounds **1** and **2** was significant only in SkHep1. The activity levels in response to derivatives also reflected concentration-dependent effects (Figure S3).

**Figure 7:**
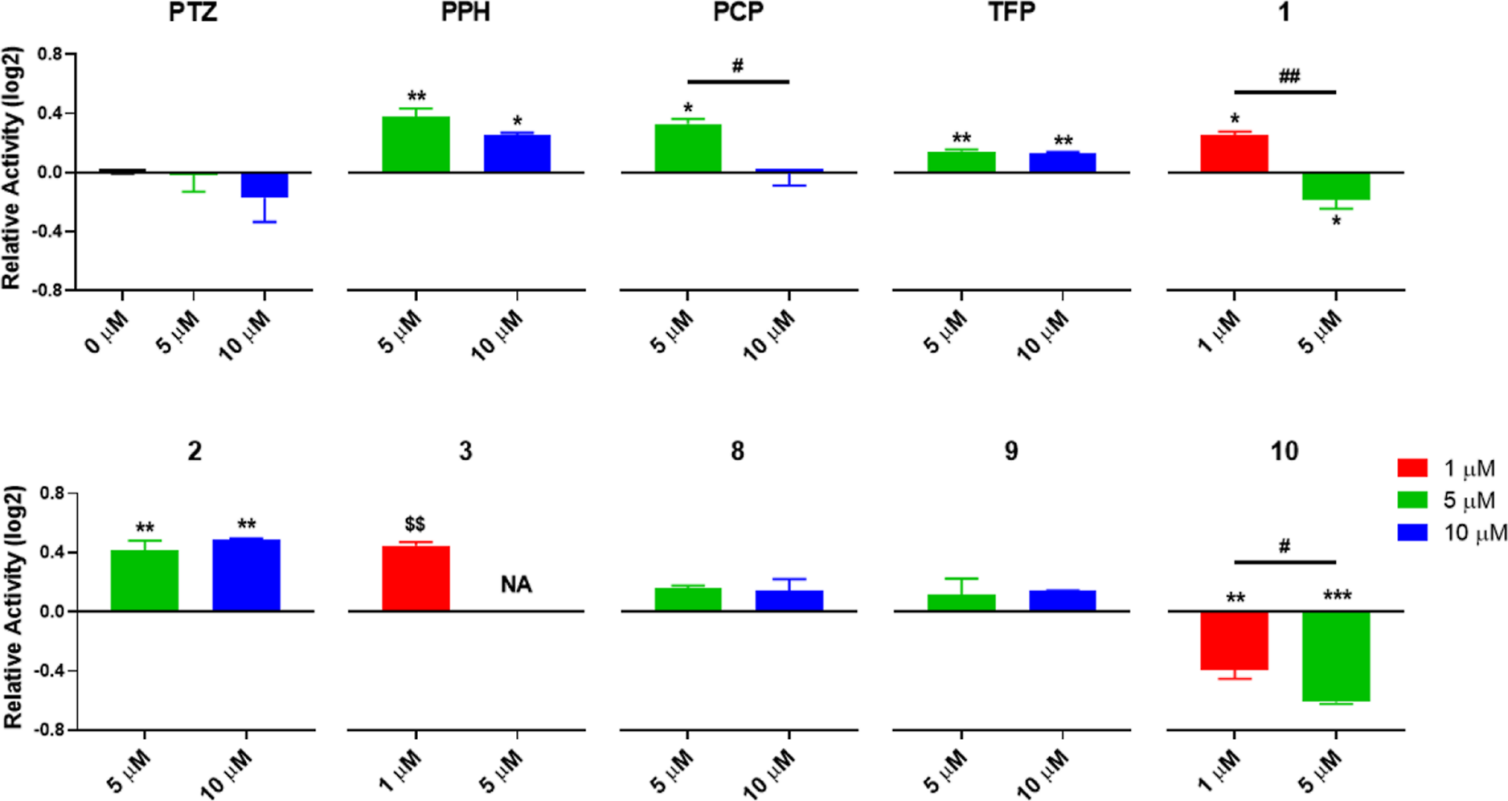
Zebrafish embryo cholinesterase activity levels after 48–120 hpf exposures: one-way ANOVA/Tukey tests with respect to DMSO control or across the applied concentrations, respectively (*p*-values: *,# ≤ 0.05, **,## ≤ 0.01, and ***,### ≤ 0.001), or unpaired *t* tests against DMSO control where total mortality was observed for the secondary groups (NAs) ($$ ≤ 0.01).

Zebrafish has been coined as a good model for studying AChE activity because it has no *bche* gene.^66,67^ We screened the prominent compounds in developing embryos exposed to drugs between 48 and 120 hpf and discovered a general activatory trend overall, except for the derivative **10** that exhibited significant inhibitory effects (Figure 7). Interestingly, the cholinesterase activity was also dependent on the drug concentration in zebrafish where the higher doses showed a tendency to lower the cholinesterase activity (Figure 7), possibly through allosteric effects on AChE structures.^68,69^

### 2.8 Changes in *ACHE* mRNA Expression in Dose- and Cell-Dependent Manners

**Figure 8:**
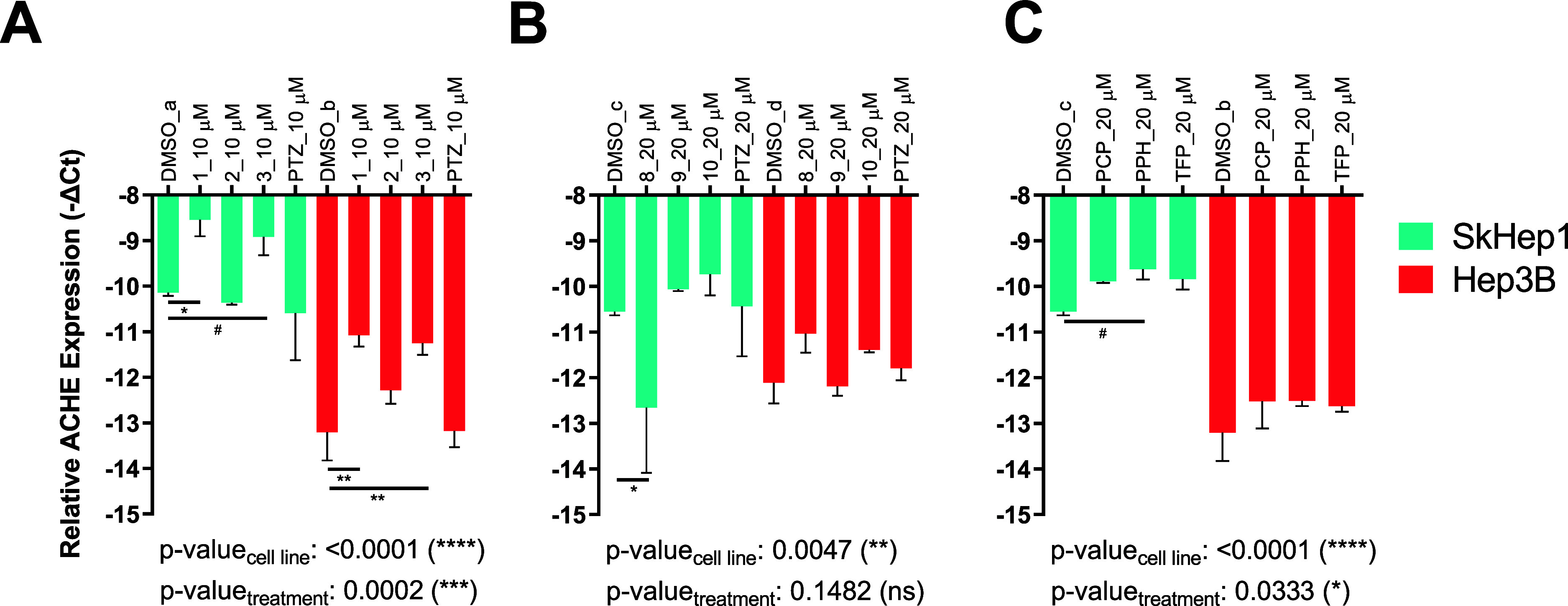
Expression of ACHE in SkHep1 and Hep3B cells, respectively, after treatment with (A) **1**, **2**, **3**, PTZ at 10 μM; (B) **8**, **9**, **10**, PTZ at 20 μM; and (C) PCP, PPH, TFP at 20 μM for 24 h. While the *y*-axis shows relative ACHE expression to TPT1 reference gene as—DeltaCt, two-way ANOVA followed by Sidak’s test was used to compare each treatment group to a batch and cell-line specific DMSO control group, indicated as DMSO_a–d. Main group tests are reported on graphs as cell line and treatment-specific *p*-values (*: *p* ≤ 0.05, **: *p* ≤ 0.01, ***: *p* ≤ 0.001, ****: *p* ≤ 0.0001, and #: *p* ≤ 0.1).

We also tested the effects of selected derivatives on the mRNA levels of cholinesterases. In particular, we found that the endogenous amount of *ACHE* mRNA was relatively and significantly lower in Hep3B cells in comparison with that of SkHep1 cells (Figure 8). 10 μM of intermediate compounds **1** and **3**, but not **2** nor PTZ, increased significantly the *ACHE* mRNA level, when compared to the DMSO control group in both cell lines (Figure 8A). Accordingly, only compound **1** increased both the cholinesterase activity as well as *ACHE* mRNA and only in SkHep1 cells (Figure 8A).

On the other hand, the novel derivatives caused no significant changes in the *ACHE* mRNA expression in Hep3B cells even at 20 μM although they had significant effects on the enzyme activity (Figure 8B). On the other hand, compound **8** resulted in a significant decrease of *ACHE* expression in SkHep1 cells, yet no effect was seen at the level of cholinesterase activity, unlike in Hep3B (Figure 8B). Finally, known PTZ derivatives did not show any effect on the amount of *ACHE* mRNA in either cell line (Figure 8C). Overall, the pattern of mRNA expression in response to phenothiazines was similar in direction (except compounds **8** and **9**) between the two cell lines. On the other hand, *BCHE* mRNA levels in response to the compounds did not vary as in the case of *ACHE* in the SkHep1 cells, and TFP was the only molecule that lowered the expression of *BCHE* significantly (Figure S4). In addition, *BCHE* was expressed in a very low amount in Hep3B cells, and its response to drugs could not be quantified. We also analyzed expression levels and DNA copy numbers of *ACHE* and *BCHE* in different liver cancer cells, which may help select other cell lines for future studies (Figure S5).

### 2.9 Embryonic Toxicity Profiles of the Compounds

**Figure 9:**
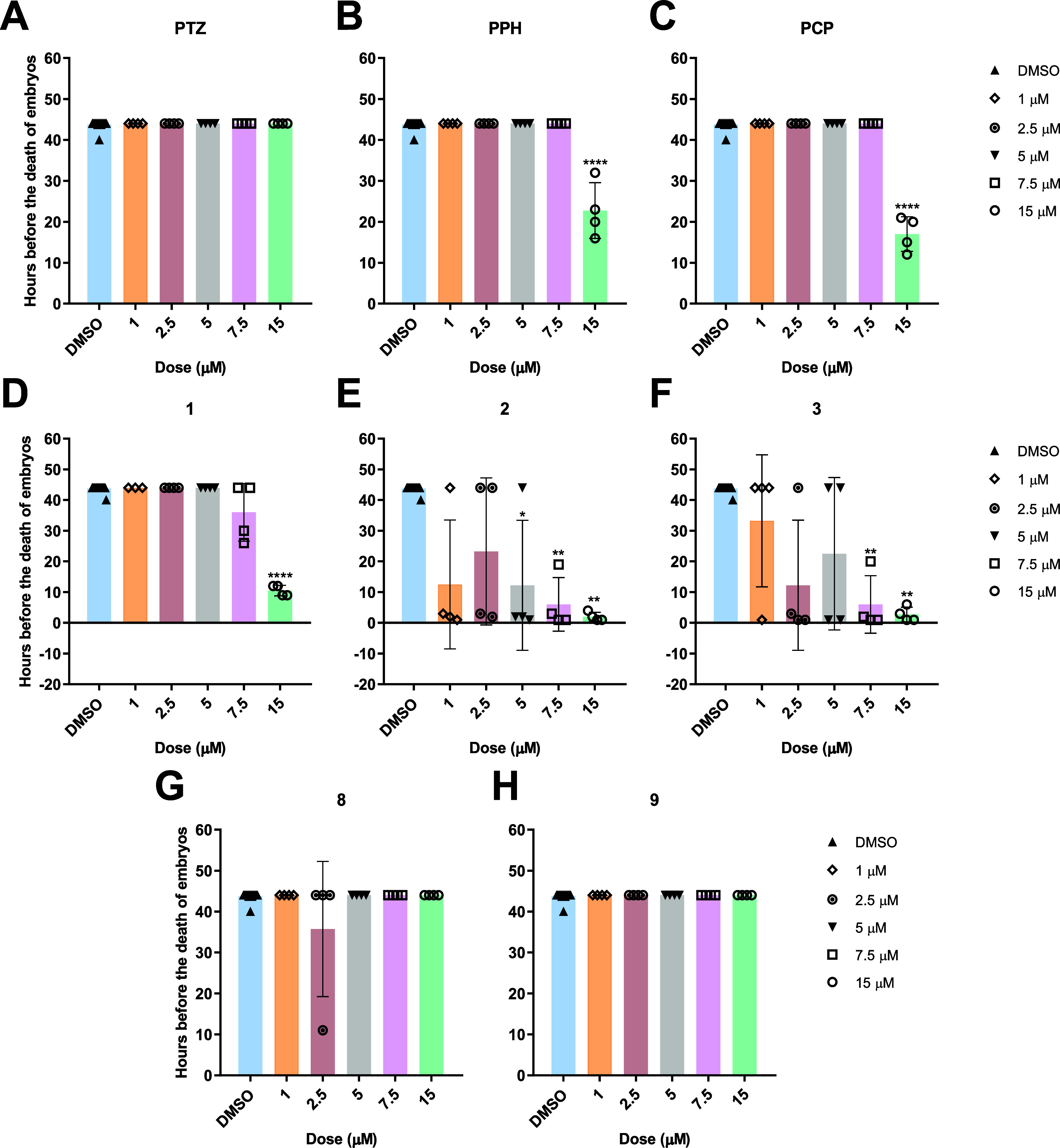
Number of hours before the death of the embryos at 9–24 hpf stage after being treated with different concentrations of known phenothiazines (A) PTZ, (B) PPH, (C) PCP, intermediate phenothiazines (D) **1**, (E) **2**, (F) **3**, and novel phenothiazines (G) **8** and (H) **9**. The statistical analysis was performed using the Kruskal–Wallis test (*: *p* ≤ 0.05, **: *p* ≤ 0.01, ***: *p* ≤ 0.001, and ****: *p* ≤ 0.0001).

PTZ, PPH, PCP, and compounds **1**, **2**, **3**, **8**, and **9** were studied on zebrafish embryos staged 9–24 hpf with respect to the rate of mortality; 15 μM PCP and PPH were highly lethal while PTZ did not affect the embryos at any doses (Figure 9A–C). Among intermediate derivatives, compound **1** showed a significant toxicity only at the highest dose tested while compounds **2** and **3** were highly toxic (Figure 9D–F). Accordingly, compound **1** could be put forward as a lead molecule with high in vitro cytotoxicity and relatively low in vivo teratogenecity (Figures 2A and 9D). On the other hand, exposure with novel derivatives **8** and **9** did not have a significant toxic effect on viability of the embryos as PTZ, suggesting that they were potentially safe molecules up until 15 μM, yet more cytotoxic than PTZ in vitro in the SkHep1 and Hep3B cells (Figures 9G–H; 2A).

**Figure 10:**
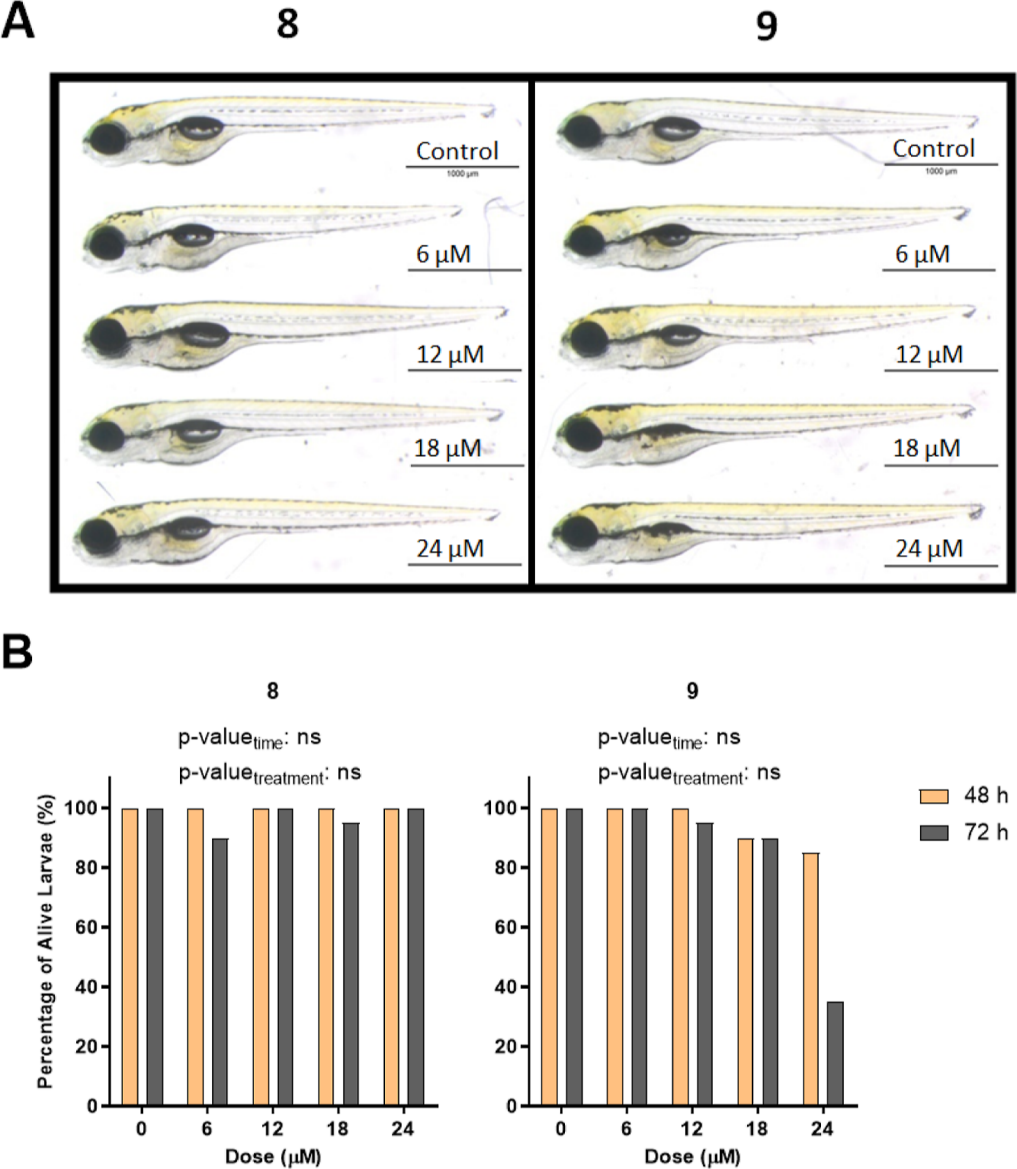
(A) Representative images of 5 dpf larvae after 72 h of exposure with novel phenothiazines **8** and **9**. (B) Percentage of alive larvae after treatment with different concentrations of compounds **8** and **9** for 48 and 72 h starting from 2 dpf. The statistical analysis was performed using two-way ANOVA (*: *p* ≤ 0.05, **: *p* ≤ 0.01, ***: *p* ≤ 0.001, and ****: *p* ≤ 0.0001).

Since compounds **8** and **9** did not seem to be toxic up to 15 μM concentration (Figure 9), the interval for doses was extended up to 24 μM, and no major morphological abnormality was observed in the larvae treated when compared to DMSO control (Figure 10A) although those exposed to compound **9** showed impairment in the development of swim bladder and had slimmer yolks. Moreover, while 72 h of exposure to compound **9** resulted in mortality at 24 μM to a large extent, compound **8** was not as toxic even at the highest dose tested (Figure 10B).

**Figure 11:**
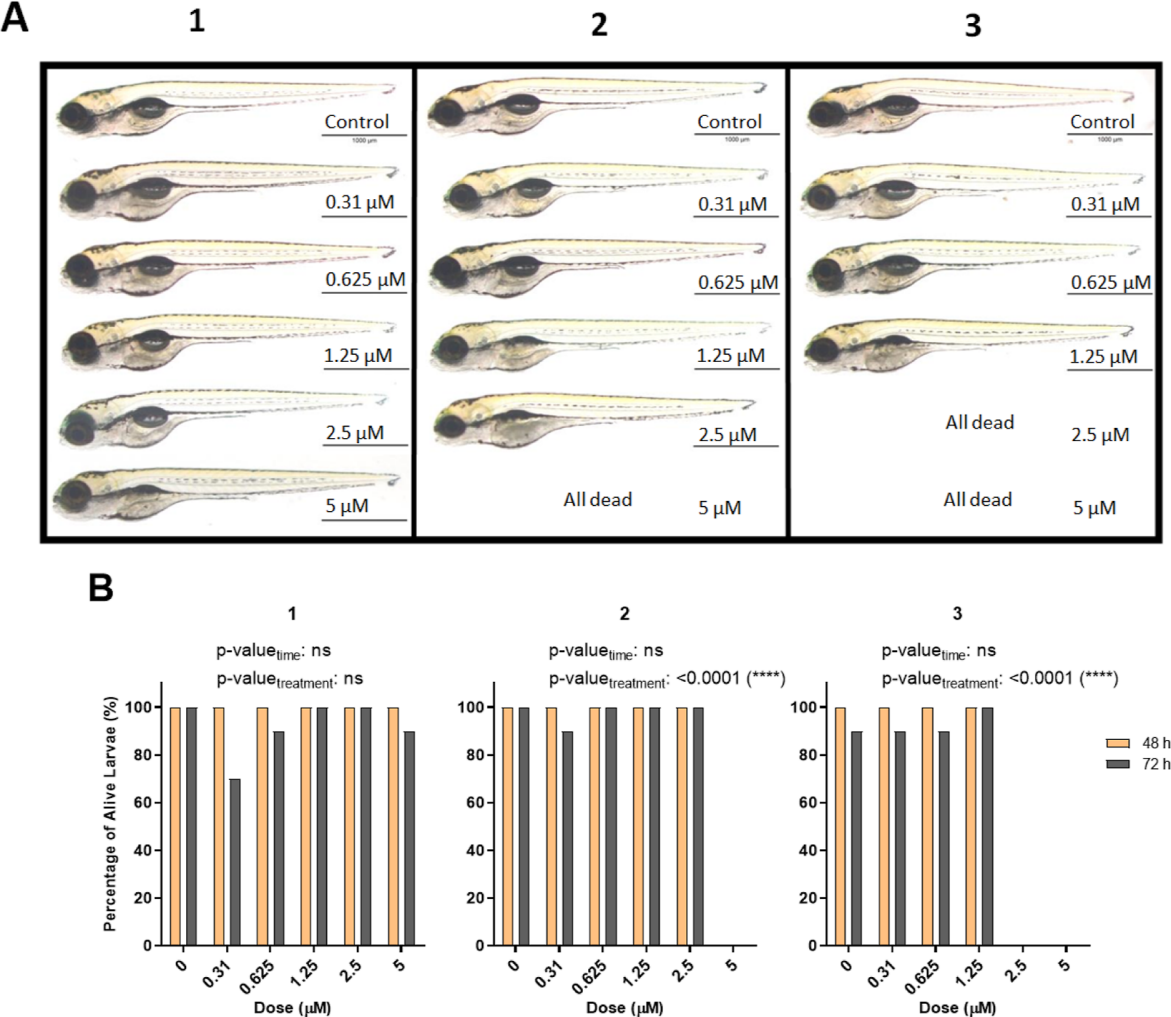
(A) Representative images of 5 dpf larvae after 72 h of exposure with intermediate phenothiazines **1**, **2**, and **3**. (B) Percentage of alive larvae after treatment with different concentrations of compounds **1**, **2**, and **3** for 48 and 72 h starting from 2 dpf. The statistical analysis was performed using two-way ANOVA (*: *p* ≤ 0.05, **: *p* ≤ 0.01, ***: *p* ≤ 0.001, and ****: *p* ≤ 0.0001).

As observed in the embryonic stages, the intermediate compounds **2** and **3** were highly toxic also at 5 dpf even at low concentrations while compound **1** was relatively safe at 5 μM (Figure 11A). 72 h of exposure caused larvae to have deformed yolks at 2.5 μM of compound **2** and 1.25 μM of compound **3**, and at 5 μM, both compounds resulted in the death of all larvae (Figure 11B). These results further confirmed the relative safety of compound **1** among the three intermediate derivatives as a potential lead molecule for future tests. The compound **4** was relatively less toxic in vivo than compound **2**–**3** as evident by the tolerance of larvae exposed to concentrations less than 18 μM at both 4 and 5 dpf (Figure S6).

Novel compound **10** also did not show any significant toxicity, although a developmental delay in the formation of a swim bladder was observed at the highest dose tested at 5 dpf (Figure S7). While an exposure to 36 μM of compound **4** caused 100% mortality at 5 dpf (Figure S8A), survival was not affected by compound **10** at the same dose, making it a potential lead derivative with low in vivo toxicity (Figure S8B).

There was no significant change in larval length caused by compounds **1**, **2**, **3**, **9**, and **10** (Figures S9C, S10 and S11B), yet the mean total length of 5 dpf larvae was reduced significantly by 6 μM of compound **8** (Figure S9B), while compound **4** at doses above 12 μM resulted in a gradual decrease in the larval length (Figure S11A).

These results suggested that compounds **8** and **10** and to a lesser degree **1** were the least toxic in vivo while all exhibiting significant cytotoxicity in vitro and can be studied further as the lead molecules. Compound **1** and **10** resulting in modulations in *ACHE* mRNA levels and/or cholinesterase activity also make them safe and effective cholinesterase modulators.

## 3 Conclusions

In the present study, we have used in silico, in vitro, and in vivo approaches to test a relatively large set of phenothiazines, both novel and known, and identified several lead molecules whose exposures caused high cytotoxicity in liver cancer cells but low adverse effects on zebrafish development. Moreover, we have tested the cholinesterase activity of selected phenothiazines, based on the guides provided by in silico target search and docking analyses, demonstrating cell line and dose specific effects in vitro, complemented with in vivo zebrafish assays. Our study highlights the importance of using in vivo zebrafish models to identify less teratogenic novel PTZ leads with or without cholinesterase modulatory activities for further investigation. In particular, the cytotoxicity assays performed in Hep3B and SkHep1 cells have led to the identification of the intermediary derivatives **1** and **3** with the most profound effects on the cancer cell survival in a cell-independent manner, which was followed by the known derivatives TFP, PCP, and PPH, and the novel derivatives **8**, **9**, **10**, and **25**. Our results suggested that the anticancer effects could depend on cell-, dose-, and compound-specific structural moieties.

In addition, based on the obtained Glide scores and findings in zebrafish, where no *bche* is found, AChE modulation by phenothiazines was more likely. We confirmed that Hep3B and SkHep1 cells had lower and higher levels of basal cholinesterase activities, respectively.^70−72^ The novel compounds **8**, **9**, and **10** increased the cholinesterase activity in the Hep3B cells with a low baseline AChE activity while they did not significantly alter the *ACHE* mRNA levels. On the other hand, SkHep1 cells with higher basal levels of *ACHE* mRNA responded to compound **10** with a significant decrease in the cholinesterase activity and compound **8** with lowered *ACHE* mRNA levels. This cell line specific transcriptional *ACHE* response to phenothiazines may be reflective of a feedback response^73,74^ that can help normalize the endogenous and/or derivative-driven AChE activity in cancer cells and hence warrants further study.

Future studies can also be used to test whether depletion of *ACHE* or *BCHE* mRNA via RNAi or Crispr/Cas9 in different cell lines can modulate the observed cytotoxic profiles. In addition, targets other than cholinesterases of these intermediate and novel phenothiazines can be pursued in the future. Our in silico clustering of targets across phenothiazines suggests unique interactions of molecule **10** with dopamine receptor D2, D3, and D4, this also warrants further investigation. Although phenothiazines are well-known as dopamine receptor modulators, more studies are needed if they lead to enhanced associations between dopamine receptor and AChE activities.^75−77^

## 4 Materials and Methods

### 4.1 Chemistry

Melting points were determined with a Buchi B-540 (BuchiLabortechnik, Flawil, Switzerland) and Electrothermal 9100 capillary melting point apparatus (Electrothermal, Essex, UK) and are uncorrected. The ^1^H NMR spectra in DMSO-*d*_6_ using a Varian Mercury-400 FT-NMR spectrometer (Varian Inc., Palo Alto, CA, USA) and the mass spectra based on the ESI(+) method using Waters ZQ micromass LC–MS spectrometer (Waters Corporation, Milford, MA, USA) were recorded (Figures S12–S57). For elemental analysis, we used a LECO 932 CHNS (Leco-932, St. Joseph, MI, USA) instrument. Silica gel 60 (40 mm to 63 mm particle size) was used for column chromatography.

### 4.2 Synthesis of the Intermediate and Novel Phenothiazines

#### 4.2.1 General Procedure for Synthesis of 1–5

Synthesis of the PTZ derivatives was initiated from commercial nonsubstituted and substituted PTZ derivatives. To a solution of PTZ derivatives (2 mmol) and TEA (2.2 mmol) in 10 mL of dry THF, chloroacetyl chloride or chloropropionyl chloride in THF was added dropwise and stirred until the starting materials were consumed (rt, determined by thin-layer chromatography (TLC), 1 w). The mixture was poured on ice-cold water and extracted with diethyl ether. The organic phase was washed with 5% NaHCO_3_ and distilled H_2_O then dried with Na_2_SO_4_. Afterward, the solvent was evaporated. The residue was purified by column chromatography to give 1–5 (Scheme 1 and Table 2).^62^

**Table 2:**
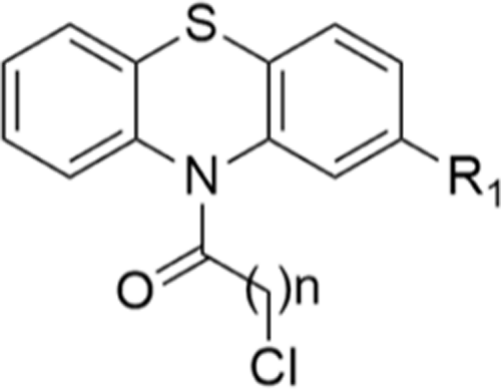
Physicochemical Data for the Synthesized Intermediates **1–5**

	*n*	R_1_	MS (ESI^+^)*m*/*z*	melting point (°C)
**1**(78)	1	–H	276	117
**2**(79)	1	–Cl	311	118
**3**(80)	1	–SCH_3_	321	124
**4**(81)	2	–H	290	144
**5**(82)	2	–Cl	325	113

#### 4.2.2 General Procedure for Synthesis of Aliphatic Amine-Substituted PTZ 10-Carboxamides (**6–26**)

1 mmol intermediate **1**–**5** in THF was added dropwise to the mixture of 1.2 mmol amine and K_2_CO_3_, and the mixture was heated under reflux until the starting material was consumed (determined by TLC, 6–8 h) (Scheme 1). Solvent was evaporated, and the residue was extracted with ethyl acetate. The organic phase was washed with water and dried with Na_2_SO_4_. The solvent was then evaporated, and the residue was purified by column chromatography.^62^

##### 4.2.2.1 *N*,*N*-Diethyl-1-(2-oxo-2-(10*H*-Phenothiazine-10-yl)ethyl)piperidine-3-carboxamide **6**

Compound **6** was prepared according to general methods starting from 2-chloro-1-(10*H*-phenothiazine-10-yl)ethan-1-one (0.150 g, 0.55 mmol) and *N,N*-diethylpiperidine-3-carboxamide (0.65 mmol, 0.12 mL). The residue was purified by cc using the mixture of chloroform/methanol (50:1) as the eluent, mp 178 °C (0.150 g, 65% yield) ^**1**^**H NMR (400 MHz, CDCl**_**3**_**)**: δ ppm 1.05 (t, 3H), 1.15 (t, 3H), 1.43–1.68 (m, 4H), 2.00 (brd s, 1H), 2.23 (brd s, 1H), 2.75 (brd d, 3H), 3.20–3.37 (m, 6H), 7.21–7.25 (m, 2H), 7.28–7.32 (m, 2H), 7.43 (d, *J* = 7.2 Hz, 2H), 7.51 (d, *J* = 7.2 Hz, 2H). ^**13**^**C NMR (CDCl**_**3**_**):** δ ppm 13.0, 14.9, 24.6, 27.2, 39.4, 40.0, 41.7, 53.5, 56.3, 60.5, 126.7, 126.9, 127.9, 133.1, 138.6, 173.0. **MS (ESI**^**+**^**)***m*/*z*: 424. **Anal. Calcd For C**_**24**_**H**_**29**_**N**_**3**_**O**_**2**_**S**: C, 68.05; H, 6.90; N, 9.92; S, 7.57; Found: C, 67.83; H, 7.12; N, 9.88; S, 7.48.

##### 4.2.2.2 2-(4-(3,4-Dichlorobenzyl)piperazine-1-yl)-1-(10*H*-phenothiazine-10-yl)ethan-1-one **7**

Compound **7** was prepared according to general methods starting from 2-chloro-1-(10*H*-phenothiazine-10-yl)ethan-1-one (0.150 g, 0.55 mmol) and 1-(3,4-dichlorobenzyl)piperazine (0.65 mmol, 0.13 mL). The residue was purified by cc using the mixture of chloroform/methanol (50:1) as the eluent, mp 148 °C (0.180 g, 68% yield). ^**1**^**H NMR (400 MHz, CDCl**_**3**_**)**: δ ppm 2.40 (brd d, 8H), 3.30 (s, 2H), 3.40 (s, 2H), 7.13 (brd d, 1H), 7.19–7.44 (m, 8H), 7.53 (d, *J* = 8.0 Hz, 2H). ^**13**^**C NMR (CDCl**_**3**_**)**: δ ppm 52.6, 52.8, 60.1, 61.5, 125.0, 126.8, 126.9, 127.8, 128.2, 130.1, 130.7, 130.8, 132.2, 133.1, 138.6, 168.6. **MS (ESI**^**+**^**)***m*/*z*: 484. **Anal. Calcd For C**_**25**_**H**_**23**_**Cl**_**2**_**N**_**3**_**O**_**2**_**S**: C, 61.98; H, 4.78; N, 8.67; S, 6.61; Found: C, 61.40; H, 4.83; N, 8.67; S, 6.51.

##### 4.2.2.3 1-(10*H*-Phenothiazine-10-yl)-2-(4-(2,3,4-trimethoxybenzyl)piperazine-1-yl)ethan-1-one **8**

Compound **8** was prepared according to general methods starting from 2-chloro-1-(10*H*-phenothiazine-10-yl)ethan-1-one (0.150 g, 0.55 mmol) and 1-(2,3,4-trimethoxybenzyl)piperazine (0.65 mmol, 0.221 g). The residue was purified by cc using the mixture of chloroform/methanol (50:1) as the eluent, mp 109 °C (0.200 g, 72.6% yield). ^**1**^**H NMR (400 MHz, CDCl**_**3**_**)**: δ ppm 2.50 (brd s, 6H), 2.72 (brd s, 1H), 3.13 (t, 1H), 3.28 (s, 2H), 3.53 (brd s, 2H), 3.84–3.87 (m, 9H), 6.61–6.64 (m, 1H), 6.93–7.01(m, 1H), 7.19–7.31 (m, 4H), 7.40–7.42 (m, 2H), 7.52 (d, *J* = 8.0 Hz, 2H). ^**13**^**C NMR (CDCl**_**3**_**)**: δ ppm, 43.9, 50.2, 52.2, 55.9, 60.0, 60.7, 61.1, 106.9, 107.0, 125.6, 126.7, 126.8, 126.9, 127.9, 133.1, 138.6, 142.1, 152.6, 153.3, 168.6. **MS (ESI**^**+**^) *m*/*z*: 506. **Anal. Calcd For C**_**28**_**H**_**31**_**N**_**3**_**O**_**4**_**S-0.7H**_**2**_**O**: C, 64.89; H, 6.30; N, 8.10; S, 6.18; Found: C, 64.91; H, 6.47; N, 8.13; S, 5.92.

##### 4.2.2.4 1-(2-Chloro-10*H*-phenothiazine-10-yl)-2-(4-(2,3,4-trimethoxybenzyl)piperazine-1-yl)ethan-1-one **9**

Compound **9** was prepared according to general methods starting from 2-chloro-1-(2-chloro-10*H*-phenothiazine-10-yl)ethan-1-one (0.150 g, 0.48 mmol) and 1-(2,3,4-trimethoxybenzyl)piperazine (0.576 mmol, 0.195 g). The residue was purified by cc using the mixture of chloroform/methanol (50:1) as the eluent, mp 94 °C (0.190 g, 61% yield). ^**1**^**H NMR (400 MHz, CDCl**_**3**_**)**: δ ppm 2.48 (brd s, 8H), 3.27 (q, 2H), 3.49 (s, 2H), 3.84–3.87 (m, 9H), 6.61–6.4 (m, 1H), 6.98 (d, *J* = 8.4 Hz, 1H), 7.17–7.33 (m, 4H), 7.40 (dd, *J* = 1.6 Hz, *J* = 8.0 Hz, 1H), 7.50 (d, *J* = 7.6 Hz, 1H), 7.64 (d, *J* = 1.6 Hz, 1H) ^**13**^**C NMR (CDCl**_**3**_**)**: δ ppm, 52.4, 52.5, 55.9, 56.1, 60.2, 60.7, 61.1, 106.9, 125.3, 126.7, 126.8, 127.0, 127.1, 127.9, 128.3, 132.6, 138.2, 139.8, 142.2, 152.6, 168.5. **MS (ESI**^**+**^**)***m*/*z*: 541. **Anal. Calcd For C**_**28**_**H**_**30**_**ClN**_**3**_**O**_**4**_**S-0.3H**_**2**_**O**: C, 61.65; H, 5.65; N, 7.70; S, 5.87; Found: C, 61.57; H, 6.19; N, 7.70; S, 5.34.

##### 4.2.2.5 1-(2-Chloro-10*H*-phenothiazine-10-yl)-2-(4-(4-methoxybenzyl)piperazine-1-yl)ethan-1-one **10**

Compound **10** was prepared according to general methods starting from 2-chloro-1-(2-chloro-10*H*-phenothiazine-10-yl)ethan-1-one (0.150 g, 0.48 mmol) and 1-(4-methoxybenzyl)piperazine (0.58 mmol, 0.118 g). The residue was purified by cc using the mixture of chloroform/methanol (50:1) as the eluent. HCl was added to the solution of product in EtOH to create the salt form, mp 201 °C (0.160 g, 64.8% yield). ^**1**^**H NMR (400 MHz, CDCl**_**3**_**)**: δ ppm 3.16 (brd s, 3H), 3.38–3.44 (m, 6H), 3.75 (s, 3H), 4.26 (brd s, 3H), 6.98 (d, *J* = 8.4 Hz, 2H), 7.33–7.44 (m, 3H), 7.53–7.59 (m, 4H), 7.71 (brd s, 1H), 7.78 (brd s, 1H). **MS (ESI**^**+**^**)***m*/*z*: 480. **Anal. Calcd For C**_**26**_**H**_**26**_**ClN**_**3**_**O**_**2**_**S-2.5HCl-0.6H**_**2**_**O:** C, 53.65; H, 5.14; N, 7.22; S, 5.50; Found: C, 53.36; H, 5.44; N, 7.28; S, 5.53.

##### 4.2.2.6 1-(2-Chloro-10*H*-phenothiazine-10-yl)-2-(4-(2-ethoxyethyl)piperazine-1-yl)ethan-1-one **11**

Compound **11** was prepared according to general methods starting from 2-chloro-1-(2-chloro-10*H*-phenothiazine-10-yl)ethan-1-one (0.48 mmol, 0.150 g) and 1-(2-ethoxyethyl)piperazine (0.58 mmol, 0.06 mL). The residue was purified by cc using the mixture of chloroform/methanol (50:1) as the eluent, mp 93 °C (0.150 g, 72% yield). ^**1**^**H NMR (400 MHz, CDCl**_**3**_**)**: δ ppm 1.17 (t, 3H), 2.47–2.57 (m, 10H), 3.26 2 (d, 2H), 3.47 (q, 2H), 3.53 (t, 2H), 7.18–7.34 (m, 4H), 7.44 (dd, *J* = 1.2 Hz, *J* = 7.8 Hz, 1H), 7.51 (dd, *J* = 1.2 Hz, *J* = 7.8 Hz, 1H), 7.65 (d, *J* = 2.0 Hz, 1H). ^**13**^**C NMR (CDCl**_**3**_**)**: δ ppm, 15.1, 52.5, 53.3, 57.7, 60.3, 66.4, 67.9, 126.7, 126.8, 127.0, 127.1, 127.9, 128.3, 132.6, 138.3, 139.8, 168.4. **MS (ESI**^**+**^**)***m*/*z*: 432. **Anal. Calcd For C**_**22**_**H**_**26**_**ClN**_**3**_**O**_**2**_**S**: C, 61.16; H, 6.06; N, 9.72; S, 7.42; Found: C, 61.24; H, 6.29; N, 9.40; S, 7.23.

##### 4.2.2.7 1-(2-Chloro-10*H*-phenothiazine-10-yl)-2-(4-(pyrimidine-2-yl)piperazine-1-yl)ethan-1-one **12**

Compound **12** was prepared according to general methods starting from 2-chloro-1-(2-chloro-10*H*-phenothiazine-10-yl)ethan-1-one (0.48 mmol, 0.150 g) and 2-(piperazine-1-yl)pyrimidine (0.576 mmol, 0.08 mL). The residue was purified by cc using the mixture of chloroform/methanol (50:1) as the eluent, mp 186 °C (0.130 g, 61.7% yield). ^**1**^**H NMR (400 MHz, CDCl**_**3**_**)**: δ ppm 2.50 (t, 4H), 3.38 (s, 2H), 3.70 (t, 4H), 6.44 (t, 1H), 7.20–7.24 (m, 1H), 7.30(dd, *J* = 7.6 Hz, *J* = 1.6 Hz, 1H), 7.33 (d, *J* = 1.6 Hz, 1H), 7.44 (dd, *J* = 7.8 Hz, *J* = 1.6 Hz, 2H), 7.55 (d, *J* = 7.6 Hz, 2H), 8.25 (d, *J* = 4.4 Hz, 2H). ^**13**^**C NMR (CDCl**_**3**_**)**: δ ppm 43.4, 52.5, 60.2, 109.8, 126.6, 126.8, 126.9, 127.9, 133.1, 138.6, 157.6, 161.5, 168.4. **MS (ESI**^**+**^**)***m*/*z*: 438. **Anal. Calcd For C**_**22**_**H**_**20**_**ClN**_**5**_**OS**: C, 60.33; H, 4.60; N, 15.99; S, 7.32; Found: C, 60.55; H, 5.15; N, 15.06; S, 6.92.

##### 4.2.2.8 2-(4-(2-Ethoxyethyl)piperazine-1-yl)-1-(10*H*-phenothiazine-10-yl)ethan-1-one **13**

Compound **13** was prepared according to general methods starting from 2-chloro-1-(10*H*-phenothiazine-10-yl)ethan-1-one (0.48 mmol, 0.150 g) and 1-(2-ethoxyethyl)piperazine (0.65 mmol, 0.11 mL). The residue was purified by cc using the mixture of chloroform/methanol (50:1) as the eluent, mp 112 °C (0.140 g, 64% yield). ^**1**^**H NMR (400 MHz, CDCl**_**3**_**)**: δ ppm 1.17 (t, 3H), 2.47 (brd s, 8H), 2.56 (t, 2H), 3.28 (s, 2H), 3.46 (q, 2H), 3.53 (t, 2H), 7.19 (d, *J* = 1.6 Hz, 1H), 7.22 (dd, *J* = 7.4 Hz, *J* = 1.6 Hz, 1H), 7.28 (d, *J* = 1.6 Hz, 1H), 7.30 (dd, *J* = 7.4 Hz, *J* = 1.6 Hz, 1H), 7.43 (dd, *J* = 7.6 Hz, *J* = 1.6 Hz, 2H), 7.53 (d, *J* = 8.0 Hz, 2H). ^**13**^**C NMR (CDCl**_**3**_**)**: δ ppm 15.1, 52.5, 53.3, 57.7, 60.2, 66.4, 67.8, 126.7, 126.9, 127.8, 133.1, 138.6, 168.6. **MS (ESI**^**+**^**)***m*/*z*: 398. **Anal. Calcd For C**_**22**_**H**_**27**_**N**_**3**_**O**_**2**_**S**: C, 66.47; H, 6.48; N, 10.57; S, 8.06; Found: C, 66.07; H, 7.14; N, 10.59; S, 8.04.

##### 4.2.2.9 1-(10*H*-Phenothiazine-10-yl)-2-(4-(pyrimidine-2-yl)piperazine-1-yl)ethan-1-one **14**

Compound **14** was prepared according to general methods starting from 2-chloro-1-(10*H*-phenothiazine-10-yl)ethan-1-one (0.48 mmol, 0.150 g) and 2-(piperazine-1-yl)pyrimidine (0.48 mmol, 0.07 mL). The residue was purified by cc using the mixture of chloroform/methanol (50:1) as the eluent, mp 178 °C (0.110 g, 56.2% yield). ^**1**^**H NMR (400 MHz, CDCl**_**3**_**)**: δ ppm 2.46 (t, 4H), 3.36 (s, 2H), 3.68 (t, 4H), 7.20–7.25 (m, 2H), 7.30 (d, *J* = 1.6 Hz, 1H), 7.33 (dd, *J* = 7.8 Hz, *J* = 1.6 Hz, 1H), 7.30 (d, *J* = 1.6 Hz, 1H), 7.44 (dd, *J* = 7.8 Hz, *J* = 1.2 Hz, 2H), 7.56 (d, *J* = 7.6 Hz, 2H), 8.26 (d, *J* = 4.4 Hz, 2H). ^**13**^**C NMR (CDCl**_**3**_**)**: δ ppm 43.5, 52.6, 60.4, 109.8, 126.6, 126.8, 127.9, 133.2, 138.7, 157.6, 161.6, 168.6. **MS (ESI**^**+**^**)***m*/*z*: 404. **Anal. Calcd For C**_**22**_**H**_**21**_**N**_**5**_**OS**: C, 65.48; H, 5.27; N, 17.35; S, 7.94; Found: C, 65.07; H, 5.47; N, 16.90; S, 7.91.

##### 4.2.2.10 2-(4-(2-Ethoxyethyl)piperazine-1-yl)-1-(10*H*-phenothiazine-10-yl)propan-1-one **15**

Compound **15** was prepared according to general methods starting from 3-chloro-1-(10*H*-phenothiazine-10-yl)propan-1-one (0.48 mmol, 0.150 g) and 1-(2-ethoxyethyl)piperazine (0.62 mmol, 0.1 mL). The residue was purified by cc using the mixture of chloroform/methanol (50:1) as the eluent, mp 212 °C (0.060 g, 29% yield). ^**1**^**H NMR (400 MHz, DMSO)**: δ ppm 1.12 (t, 3H), 3.07 (s, 2H), 3.32–3.75 (m, 16H), 7.31 (d, *J* = 7.6 Hz, 1H), 7.34 (s, 1H), 7.40 (d, *J* = 7.6 Hz, 1H), 7.43 (s, 1H), 7.57 (d, *J* = 8.0 Hz, 2H), 7.68 (d, *J* = 7.2 Hz, 2H). ^**13**^**C NMR (DMSO)**: δ ppm 14.7, 49.4, 53.0, 55.4, 57.9, 60.3, 66.1, 70.8, 127.1, 127.2, 127.8, 131.3, 132.1, 137.7, 168.0. **MS (ESI**^**+**^**)***m*/*z*: 412. **Anal. Calcd For C**_**22**_**H**_**26**_**ClN**_**3**_**O**_**2**_**S-0.2CHCl**_**3**_**-1.5 H**_**2**_**O**: C, 55.21; H, 6.09; N, 8.70; S, 6.64; Found: C, 55.14; H, 6.65; N, 8.56; S, 6.43.

##### 4.2.2.11 3-(4-(4-Methoxyphenyl)piperazin-1-yl)-1-(10*H*-phenothiazine-10-yl)propan-1-one **16**

Compound **16** was prepared according to general methods starting from 3-chloro-1-(10*H*-phenothiazine-10-yl)propan-1-one (0.48 mmol, 0.150 g) and 1-(4-methoxyphenyl)piperazine (0.62 mmol, 0.1 mL). The residue was purified by cc using the mixture of chloroform/methanol (50:1) as the eluent, mp 212 °C (0.040 g, 18% yield). ^**1**^**H NMR (400 MHz, CDCl**_**3**_**)**: δ ppm 3.1–3.57 (m, 10H), 3.75 (s, 3H), 4.27 (s, 2H), 6.97 (d, *J* = 8.4 Hz, 2H), 7.29–7.41 (m, 4H), 7.52–7.57 (m, 4H), 7.66 (d, *J* = 7.6 Hz, 2H). **MS (ESI**^**+**^**)***m*/*z*: 460. **Anal. Calcd For C**_**26**_**H**_**27**_**N**_**3**_**O**_**2**_**S-0.72CHCl**_**3**_**:** C, 60.37; H, 5.26; N, 7.90; S, 6.03; Found: C, 60.18; H, 5.97; N, 7.96; S, 6.02.

##### 4.2.2.12 2-((4-Fluorobenzyl)amino)-1-(10*H*-phenothiazine-10-yl)ethanone **17**

Compound **17** was prepared according to general methods starting from 2-chloro-1-(10*H*-phenothiazine-10-yl)ethan-1-one (0.54 mmol, 0.150 g) and 4-fluorobenzylamine (0.54 mmol, 0.061 mL). The residue was purified by cc using the mixture of chloroform/hexane (4:1) as the eluent, mp 88.1 °C (0.060 g, 40% yield). ^**1**^**H NMR (400 MHz, CDCl**_**3**_**)**: δ ppm 2.09 (brd s, 1H), 3.48 (s, 2H), 3.66 (s, 2H), 6.92–6.96 (m, 2H), 7.21–7.32 (m, 6H), 7.43–7.45 (m, 4H). ^**13**^**C NMR (CDCl**_**3**_**)**: δ ppm 50.2, 52.3, 115.1 (C–F, d, ^2^*J*_C–F_ = 21.2 Hz), 126.8, 127.1, 128.0, 129.8 (C–F, d, ^3^*J*_C–F_ = 8.4 Hz), 133.0, 135.1 (C–F, d, ^4^*J*_C–F_ = 3.0 Hz), 137.9, 160.7–163.1(C–F, d, ^1^*J*_C–F_ = 243 Hz). **MS (ES**^**+**^**)***m*/*z*: 465. **Anal. Calcd For C**_**21**_**H**_**17**_**FN**_**2**_**OS-0,35H**_**2**_**O:** C, 68.03; H, 4.81; N, 7.55; S, 8.64; Found: C, 68.34; H, 5.34; N, 7.17; S, 8.10.

##### 4.2.2.13 2-((3,4-Dichlorobenzyl)amino-1-(10*H*-phenothiazine-10-yl)ethanone **18**

Compound **18** was prepared according to general methods starting from 2-chloro-1-(10*H*-phenothiazine-10-yl)ethan-1-one (0.54 mmol, 0.150 g) and 3,4-dichlorobenzylamine (0.54 mmol, 0.072 mL). The residue was purified by cc using the mixture of chloroform/hexane (4:1) as the eluent, mp 145.6 °C (0.030 g, 20% yield). ^**1**^**H NMR (400 MHz, CDCl**_**3**_**)**: δ ppm 3.46 (s, 2H), 3.65 (s, 2H), 7.10 (dd, *J* = 8.0 Hz, *J* = 2.0 Hz, 1H), 7.22–7.26 (m, 3H), 7.29–7.33 (m, 2H), 7.38 (d, *J* = 2.0 Hz, 1H), 7.45 (dd, *J* = 8.0 Hz, 4H). ^**13**^**C NMR (DMSO)**: δ ppm 49.5, 50.6, 127.0, 127.1, 127.2, 127.8, 128.1, 128.9, 129.7, 130.1, 130.6, 132.0, 137.8, 141.5, 169.8. **MS (ES**^**+**^**)***m*/*z*: 415. **Anal. Calcd For C**_**21**_**H**_**16**_**Cl**_**2**_**N**_**2**_**OS:** C, 60.73; H, 3.88; N, 6.74; S, 7.72; Found: C, 60.45; H, 3.89; N, 6.91; S, 7.72.

##### 4.2.2.14 2-((3,4-Difluorobenzyl)amino)-1-(10*H*-phenothiazine-10-yl)ethanone **19**

Compound **19** was prepared according to general methods starting from 2-chloro-1-(10*H*-phenothiazine-10-yl)ethan-1-one (0.54 mmol, 0.150 g) and 3,4-difluorobenzylamine (0.54 mmol, 0.063 mL). The residue was purified by cc using the mixture of hexane/chloroform:ethyl acetate (4:1:2) as the eluent, mp 70.4 °C (0.040 g, 27% yield). ^**1**^**H NMR (400 MHz, CDCl**_**3**_**)**: δ ppm 3.41 (s, 2H), 3.59 (s, 2H), 7.03 (s, 1H), 7.26–7.35 (m, 6H), 7.52–7.59 (m, 4H). ^**13**^**C NMR (CDCl**_**3**_**)**: δ ppm 50.3, 52.0, 116.8, 117.0 (t, *J* = 17,5 Hz), 124.0, 126.8, 127.1, 127.2, 128.1, 133.1, 136.7, 137.9, 149.4 (dd, *J* = 245 Hz, *J* = 12,5 Hz), 150.8 (dd, *J* = 245 Hz, *J* = 13.75 Hz), 170.6. **MS (ESI**^**+**^**)***m*/*z*: 383. **Anal. Calcd For C**_**21**_**H**_**16**_**F**_**2**_**N**_**2**_**OS-0,45H**_**2**_**O:** C, 64.58; H, 4.36; N, 7.17; S, 8.21; Found: C, 64.57; H, 4.45; N, 7.05; S, 7.93.

##### 4.2.2.15 2-(Benzylamino)-1-(2-chloro-10*H*-Phenothiazine-10-yl)ethanone **20**

Compound **20** was prepared according to general methods starting from 2-chloro-1-(2-chloro-10*H*-phenothiazine-10-yl)ethan-1-one (0.26 mmol, 0.100 g) and benzylamine (0.312 mmol, 0.034 mL). The residue was purified by cc using the mixture of hexane/chloroform:ethyl acetate (4:1:2) as the eluent, mp 115.8 °C (0.043 g, 43% yield). ^**1**^**H NMR (400 MHz, DMSO)**: δ ppm 2.37 (brd s, 1H), 3.41 (s, 2H), 3.58 (s, 2H), 7.14–7.24 (m, 5H), 7.27–7.31 (m, 1H), 7.34–7.38 (m, 2H), 7.53–7.58 (m, 3H), 7.71 (d, *J* = 2.0 Hz, 1H). ^**13**^**C NMR (DMSO)**: δ ppm 49.7, 52.0, 126.6, 127.0, 127.0, 127.3, 127.5, 127.9, 128.0, 128.0, 129.0, 131.1, 131.5, 131.7, 137.3, 139.1, 140.0, 169.9. **MS (ESI**^**+**^**)***m*/*z*: 382. **Anal. Calcd For C**_**21**_**H**_**17**_**ClN**_**2**_**OS**: C, 66.22; H, 4.50; N, 7.35; S, 8.42; Found: C, 66.31; H, 4.71; N, 7.49; S, 8.38.

##### 4.2.2.16 2-(Benzylamino)-1-(10*H*-Phenothiazine-10-yl)ethanone **21**

Compound **21** was prepared according to general methods starting from 2-chloro-1-(10*H*-phenothiazine-10-yl)ethan-1-one (0.43 mmol, 0.120 g) and benzylamine (0.516 mmol, 0.046 mL). The residue was purified by cc using the mixture of hexane/ethyl acetate (3:1) as the eluent, mp 113.5 °C (0.050 g, 41.6% yield). ^**1**^**H NMR (400 MHz, DMSO)**: δ ppm 2.39 (brd s, 1H), 3.39 (brd s, 2H), 3.57 (s, 2H), 7.15–7.24 (m, 5H), 7.26–7.29 (m, 2H), 7.33–7.37 (m, 2H), 7.56 (m, 4H). ^**13**^**C NMR (DMSO)**: δ ppm 49.7, 52.0, 126.6, 127.1, 127.1, 127.2, 127.9, 127.9, 128.0, 132.0, 137.9, 140.0, 170.0. **MS (ESI**^**+**^**)***m*/*z*: 347. **Anal. Calcd For C**_**21**_**H**_**18**_**N**_**2**_**OS**: C, 72.80; H, 5.24; N, 8.09; S, 9.25; Found: C, 72.65; H, 5.54; N, 8.05; S, 9.09.

##### 4.2.2.17 2-((4-Chlorobenzyl)amino)-1-(10*H*-phenothiazine-10-yl)ethanone **22**

Compound **22** was prepared according to general methods starting from 2-chloro-1-(10*H*-phenothiazine-10-yl)ethan-1-one (0.53 mmol, 0.150 g) and 4-chlorobenzylamine (0.534 mmol, 0.065 mL). The residue was purified by cc using the mixture of chloroform/ethyl acetate (8:1) as the eluent, mp 108.2 °C (0.016 g, 10.6% yield). ^**1**^**H NMR (400 MHz, DMSO)**: δ ppm 3.38 (brd s, 2H), 3.57 (s, 2H), 7.21 (d, *J* = 8.4 Hz, 2H), 7.28 (m, 4H), 7.37 (m, 2H), 7.56 (m, 4H). ^**13**^**C NMR (DMSO)**: δ ppm 49.7, 51.2, 127.1, 127.1, 127.2, 127.3, 127.9, 129.7, 131.0, 132.0, 137.9, 139.2, 169.9. **MS (ESI**^**+**^**)***m*/*z*: 381. **Anal. Calcd For C**_**21**_**H**_**17**_**ClN**_**2**_**O**_**S**_**-0,2H**_**2**_**O**: C, 65.59; H, 4.56; N, 7.28; S, 8.33; Found: C, 65.53; H, 4.64; N, 7.38; S, 8.31.

##### 4.2.2.18 3-(Benzylamino)-1-(10*H*-Phenothiazine-10-yl)propan-1-one **23**

Compound **23** was prepared according to general methods starting from 3-chloro-1-(10*H*-phenothiazine-10-yl)propan-1-one (1.035 mmol, 0.300 g) and benzylamine (1.242 mmol, 0.135 mL). The residue was purified by cc using the mixture of chloroform/hexane (13:1) as the eluent, mp 81.9 °C (0.042 g, 14% yield). ^**1**^**H NMR (400 MHz, CDCl3)**: δ ppm 2.76 (brd s, 3H, CH_2_+NH), 2.89 (t, 2H), 3.76 (s, 2H), 7.20–7.25 (m, 4H), 7.27–7.30 (m, 4H), 7.31–7.33 (m, 1H), 7.43 (dd, *J* = 7.6 Hz, *J* = 1.2 Hz, 2H), 7.49 (d, *J* = 8 Hz, 2H). ^**13**^**C NMR (CDCl**_**3**_**)**: δ ppm 34.4, 44.8, 53.7, 126.9, 127.0, 127.0, 127.2, 128.0, 128.2, 128,4, 138.4, 139.3, 171.3. **MS (ESI**^**+**^**)***m*/*z*: 361. **Anal. Calcd For C**_**22**_**H**_**20**_**N**_**2**_**OS-0,5H**_**2**_**O:** C, 71.51; H, 5.72; N, 7.58; S, 8.67; Found: C, 71.67; H, 5.60; N, 7.65; S, 8.82.

##### 4.2.2.19 3-((4-Chlorobenzyl)amino)-1-(10*H*-phenothiazine-10-yl)propan-1-one **24**

Compound **24** was prepared according to general methods starting from 3-chloro-1-(10*H*-phenothiazine-10-yl)propan-1-one (1.035 mmol, 0.300 g) and 4-chlorobenzylamine (1.242 mmol, 0.151 mL). The residue was purified by cc using the mixture of dichloromethane/methanol (50:1) as the eluent, mp 95.2 °C (0.035 g, 11.6% yield). ^**1**^**H NMR (400 MHz, CDCl**_**3**_**)**: δ ppm 1.9 (brd s, 1H), 2.66 (brd s, 2H), 2.84 (t, 2H), 3.68 (s, 2H), 7.18–7.33 (m, 8H), 7.46 (m, 4H). ^**13**^**C NMR (CDCl**_**3**_**)**: δ ppm 34.7, 44.8, 53.1, 126.9, 126.9, 127.2, 128.0, 128.4, 129.4, 132.5, 138.5, 138.6, 171.3. **MS (ESI**^**+**^**)***m*/*z*: 395. **Anal. Calcd For C**_**22**_**H**_**19**_**ClN**_**2**_**OS-1,5 CH**_**2**_**Cl**_**2**_: C, 54.03; H, 4.24; N, 5.36; S, 6.13; Found: C, 54.13; H, 4.20; N, 5.69; S, 6.41.

##### 4.2.2.20 3-((4-Fluorobenzyl)amino)-1-(10*H*-phenothiazine-10-yl)propan-1-one **25**

Compound **25** was prepared according to general methods starting from 3-chloro-1-(10*H*-phenothiazine-10-yl)propan-1-one (1.035 mmol, 0.300 g) and 4-fluorobenzylamine (1.242 mmol, 0.141 mL). The residue was dissolved in ethyl acetate and crystallized by using hexane, mp 87.9 °C (0.037 g, 12.3% yield). ^**1**^**H NMR (400 MHz, CDCl**_**3**_**)**: δ ppm 2.21 (brd s, 1H), 2.68 (s, 2H), 2.85 (t, 2H), 3.69 (s, 2H), 6.96 (t, *J* = 8.8 Hz, 2H), 7.20–7.25 (m, 4H), 7.31 (t, *J* = 6.8 Hz, 2H), 7.43 (d, *J* = 7.2 Hz, 2H), 7.49 (d, *J* = 8 Hz, 2H). ^**13**^**C NMR (CDCl**_**3**_**)**: δ ppm 34.6, 44.8, 53.0, 115.1 (C–F, d, ^2^*J*_C–F_ = 21.2 Hz), 126.9, 127.0, 127.2, 128.0, 129.6 (C–F, d, ^3^*J*_CF_ = 7.7 Hz), 134.2, 135.5 (C–F, d, ^4^*J*_C–F_ = 3.1 Hz), 138.4, 160.6–163.1 (C–F, d, ^1^*J*_C–F_ = 243 Hz), 171.3. **MS (ESI**^**+**^**)***m*/*z*: 379. **Anal. Calcd For C**_**22**_**H**_**19**_**FN**_**2**_**OS**: C, 69.82; H, 5.06; N, 7.40; S, 8.47; Found: C, 69.51; H, 5.29; N, 7.31; S, 8.26.

##### 4.2.2.21 3-((3,4-Difluorobenzyl)amino)-1-(10*H*-phenothiazine-10-yl)propan-1-one **26**

Compound **26** was prepared according to general methods starting from 3-chloro-1-(10*H*-phenothiazine-10-yl)propan-1-one (1.035 mmol, 0.300 g) and 3,4-difluorobenzylamine (1.242 mmol, 0.15 mL). The residue was dissolved in ethyl acetate and crystallized by using hexane, mp 87.9 °C (0.037 g, 12.3% yield). The residue was purified by cc using the mixture of chloroform/hexane (3:1) as the eluent, mp 94.7 °C (0.036 g, 12% yield). ^**1**^**H NMR (400 MHz, CDCl**_**3**_**)**: δ ppm 2.32 (s, 1H), 2.67 (brd s, 2H), 2.83 (t, 2H), 3.67 (s, 2H), 6.97–7.13 (m, 3H), 7.236 (t, 2H), 7.30–7.34 (m, 2H), 7.43–7.50 (m, 4H). ^**13**^**C NMR (CDCl**_**3**_**)**: δ ppm 34.5, 44.7, 52.6, 116.9 (C–F, d, *J* = 17,5 Hz), 116.99 (C–F, d, *J* = 17,5 Hz), 123.84, 123.86, 123.89, 123.9, 127.02, 127.05, 127.28, 128.08, 133.3, 136.8, 138.4, 149.39 (dd, *J* = 245 Hz, *J* = 12.5 Hz), 150.26 (dd, *J* = 246 Hz, *J* = 12.5 Hz), 171.0. **MS (ESI**^**+**^**)***m*/*z*: 397. **Anal. Calcd For C**_**22**_**H**_**18**_**F**_**2**_**N**_**2**_**OS-0,3H**_**2**_**O**: C, 65.75; H, 4.66; N, 6.97; S, 7.97; Found: C, 65.77; H, 4.86; N, 6.81; S, 7.74.

#### 4.2.3 General Procedure for Synthesis of Arylamine-Substituted PTZ 10-Carboxamides (**27**, **28**)

Appropriate arylamines (5 mmol) and NaI (2.5 mmol) were added to a mixture of **1** (5 mmol) and triethylamine in EtOH at rt (Scheme 1). The mixture was heated under reflux, until the starting material was consumed (determined by TLC, 10 h). The product was filtered, and the solvent was evaporized. The residue was dissolved in ethyl acetate and washed with water. The solvent was evaporated, and the residue was purified by column chromatography.

##### 4.2.3.1 1-(10*H*-Phenothiazine-10-yl)-2-(phenylamino)ethan-1-one **27**

Compound **27** was prepared according to general methods starting from 2-chloro-1-(10*H*-phenothiazine-10-yl)ethan-1-one (0.9 mmol, 0.250 g) and aniline (0.9 mmol). The residue was dissolved in ethyl acetate and crystallized by using hexane, mp 153 °C (0.115 g, 46% yield). ^**1**^**H NMR (400 MHz, CDCl**_**3**_**)**: δ ppm 3.98 (s, 2H), 6.51–6.54 (m, 2H), 6.70–6.74 (m, 1H), 7.13–7.17 (m, 2H), 7.26–7.31 (m, 2H), 7.37 (dd, *J* = 8.0 Hz, *J* = 1.6 Hz, 1H) 7.40 (d, *J* = 1.2 Hz, 1H) 7.49 (dd, *J* = 8.0 Hz, *J* = 1.6 Hz, 2H), 7.56 (d, *J* = 7.6 Hz, 2H). ^**13**^**C NMR (CDCl**_**3**_**)**: δ ppm 46.7, 113.3, 118.2, 126.8, 127.2, 127.4, 128.2, 129.2, 137.6, 146.8, 168.9. **MS (ESI**^**+**^**)***m*/*z*: 333. **Anal. Calcd For C**_**20**_**H**_**16**_**N**_**2**_**OS**: C, 72.26; H, 4.85; N, 8.43; S, 9.64; Found: C, 72.15; H, 5.05; N, 8.45; S, 9.60.

##### 4.2.3.2 1-(10*H*-Phenothiazine-10-yl)-2-((4-(trifluoromethyl)phenyl)amino)ethan-1-one **28**

Compound **28** was prepared according to general methods starting from 2-chloro-1-(10*H*-phenothiazine-10-yl)ethan-1-one (0.9 mmol, 0.250 g) and *p*-trifluoromethylaniline (0.9 mmol). The residue was purified by cc using the mixture of hexane/ethyl acetate (7:2) as the eluent, mp 189 °C (0.018 g, 7.2% yield). ^**1**^**H NMR (400 MHz, CDCl**_**3**_**)**: δ ppm 3.99 (s, 2H), 6.51 (d, *J* = 8.4 Hz, 2H), 7.26–7.42 (m, 6H), 7.51 (d, *J* = 7.6 Hz, 2H), 7.56 (d, *J* = 7.6 Hz, 2H). ^**13**^**C NMR (CDCl**_**3**_**)**: δ ppm 46.3, 112.8, 120.2, 126.6 (q, –CF_3_, *J* = 3,75 Hz), 126.8, 127.3, 127.6, 128.3, 133.4, 137.4, 148.8, 168.2. **MS (ESI**^**+**^**)***m*/*z*: 401. **Anal. Calcd For C**_**21**_**H**_**15**_**F**_**3**_**N**_**2**_**OS-0,24H**_**2**_**O**: C, 62.31; H, 3.95; N, 6.92; S, 7.92; Found: C, 62.31; H, 3.98; N, 6.84; S, 7.85.

### 4.3 Biological Activity

#### 4.3.1 Cell Culture

Human hepatoma cell line Hep3B and an endothelial cell line SkHep1, derived from an HCC patient, were grown in DMEM-low glucose (Lonza) supplemented with 10% FBS (Biowest). Main PTZ scaffold and known PTZ derivatives, i.e., PPH, PCP, and TFP, were purchased from Sigma-Aldrich. Sorafenib (SFB; Selleckchem) was used as the positive control treatment. DMSO (Applichem) was used for dissolving the screened compounds and as the negative control. 3-(4,5-Dimethylthiazol-2-yl)-2,5-diphenyltetrazolium Bromide (MTT) (Molecular Probes, Thermo Fisher Scientific) assay was performed for estimating the cell viability upon exposure to drugs, according to the manufacturer’s instructions using SDS-HCl. The cells were grown on 96-well plates with initial cell densities of 10,000 and 5000 cells/well for 24 and 48 h drug exposures, respectively; and the media were renewed daily. Meanwhile, the concentrations of 3.7, 11.1, 33.3, and 100 μM were applied for cytotoxicity measurements in triplicates. BIO-TEK/μQuant Universal Microplate spectrophotometer and BIO-TEK/KC junior software (v.1.418) were used to measure the absorbance intensity values, and the percentage of relative cell viability was calculated with respect to 0.1% DMSO control group upon subtracting the scores from the blank measurements.^83^ The analysis of MTT results was performed by estimating IC_50_ values for each compound via GraphPad Prism v6.05 (log(inhibitor)—4 parameters). Statistical analyses were conducted using one-way ANOVA on the viability scores and log-scaled IC_50_ values for each treatment. In this regard, the effects of cell lines, exposure time, side-chain modifications, as well as their interactions were evaluated in R environment v3.6.1. *ggplot2* and *ggpubr* R packages were utilized for generation of figures.^84,85^ Cell viability values across the treatment concentrations and cell line effects were further complemented with two-way ANOVA/Tukey statistics in GraphPad Prism by taking the DMSO control and differences between groups into account.

#### 4.3.2 Zebrafish Husbandry

Zebrafish embryos were raised and used for experimental purposes, in compliance with Bilkent University Ethical Committee approval (2016/7 and 2021/4) and Karlsruhe Institute of Technology (KIT) institutional guidelines. Wild-type AB (+/+) strain fish were utilized throughout the experiments.

#### 4.3.3 Zebrafish Embryonic Toxicity Assessments

The embryonic viability experiments were performed at KIT, Karlsruhe using a custom imaging station. The embryos were exposed to different doses of each drug, and a DMSO group was included in each plate, and all DMSO samples were combined before analyses. The images were obtained every hour for a total of 44 h. The number of hours each embryo survived was recorded by examining individual images, and multiple comparisons were performed using the Kruskal–Wallis test to determine the differences between the doses with respect to DMSO in the hours recorded.

#### 4.3.4 High-Resolution Imaging and Morphometrics

The experiments to take high-resolution pictures were performed in Izmir Biomedicine and Genome Center, and Bilkent University according to ethics permission 2021/4. The intermediate and novel lead compounds **1**, **2**, **3**, **4**, and **8**, **9**, **10**, respectively, were applied at 28 °C at low to high doses; and images were obtained by an Olympus SZX10 stereo microscope equipped with an SC50 camera. Measurements were made using Fiji.^86^

#### 4.3.5 Cholinesterase Activity Assays in Cell Lines and In Vivo in Zebrafish

Protein collection was performed at 4 °C unless otherwise mentioned otherwise. The Pierce BCA Protein Assay kit (Thermo Scientific) was used for measuring protein levels across the samples, according to the manufacturer’s instructions. To collect proteins from Hep3B and SkHep1 cell lines in biological duplicates, the cells were scraped in cold phosphate-buffered saline and exposed with lysis buffer [50 mM *N*-(2-hydroxyethyl)piperazine-*N*′-ethanesulfonic acid], 150 mM NaCl, 1 mM EGTA, 10% glycerol, 1% Triton X100, complete protease inhibitor (Roche), and Phosstop phosphatase inhibitor (Roche). Following brief vortexing steps and final centrifugation, the supernatant containing the protein load was collected.

Cholinesterase levels were quantified by regression from exponential curves of the kit’s standard samples, estimated in the Windows Excel 2003 environment. Activity level differences were calculated by referring to the amounts in the DMSO control group (on log2 scale) and then normalized to the protein concentrations.^87,88^ The one-way ANOVA/Dunnett’s multiple comparisons test was applied in evaluating the effects on Hep3B and SkHep1 cells, while multiple *t* tests/Holm-Sidak corrections were made in estimating cell type-dependence for each separate exposure. In addition, Pearson’s correlations were calculated and plotted using ggscatter function in the *ggpubr* package in assessing relationships between IC_50_, cholinesterase activities, and docking scores.

For cholinesterase activity assays in zebrafish, drugs were applied between 48 and 120 hpf for various concentrations (1, 5, and 10 μM). To do so, 15 embryos per biological group in duplicate wells were studied, and the embryo medium was renewed on a daily basis. For further practices, the embryos were euthanized on ice. In retrieving protein samples from the zebrafish embryos, Tris-HCl (50 mM, pH: 8) exposure and homogenization via syringes were performed on ice. After continuous centrifugation steps, supernatants containing the majority of the protein content were kept for further use. The cholinesterase activity was measured by using the colorimetric AChE assay kit (ABCAM, USA), following the manufacturer’s instructions. Activity levels were normalized to the protein concentration. GraphPad Prism and one-way ANOVA/Tukey was used to assess the changes in zebrafısh AChE activity across multiple compounds and doses. Alternatively, effects of the compound **3** were analyzed via unpaired *t*-test due to single concentration point exposures.

#### 4.3.6 qRT-PCR Analyses

Effects of selected compounds on *ACHE* and *BCHE* mRNA expression were evaluated via qRT-PCR (LightCycler 480 II—Roche). Initially total RNA from the biological duplicates were collected by using RNeasy Mini Kit (Qiagen), in accordance with manufacturer’s instructions. RNA was converted into cDNA via the RevertAid First Strand cDNA synthesis kit (Fermentas). The primer sets used for qRT-PCR studies were 5′- GATCGCGGACGGGTTGT-3′ and 5′- TTCAGCGGAGGCATTTCC-3′ for *TPT1*, 5′-TCTCGAAACTACACGGCAGA-3′ and 5′-CGCAGGTCCAGACTAACGTA-3′ for *ACHE*, 5′-AGAATGGATGGGAGTGATGC-3′ and 5′-AGGCCAGCTTGTGCTATTGT-3′ for *BCHE* gene. An analysis using −ΔCt values was applied in estimating the expression level of *ACHE* with respect to that of *TPT1* reference gene for each compound.^89^ Two-way ANOVA/Sidak’s multiple comparisons test was performed to compare each treatment group with DMSO control of the corresponding batch using GraphPad Prism.

### 4.4 *In Silico* Analyses

#### 4.4.1 *In Silico* Target Screens

Simplified Molecular Input Line Entry System (SMILES) designations for the derivatives were retrieved. Canonical SMILES of the PTZ were obtained from PubChem.^90^ For the PTZ derivatives, SMILES’s were generated by using the Marvin tool at ChemAxon (http://www.chemaxon.com). Afterward, their binding probabilities toward possible targets were calculated. For this purpose, the SwissTargetPrediction tool utilizing structure similarity search was executed.^91^ Structure similarity search was performed based on the idea that if two molecules possessed similar 2D/3D structure similarity, they might have similar molecular targets/biological activities. A query molecule (PTZ derivative) was submitted to SwissTargetPrediction server as SMILES, then *Homo sapiens* library was selected as a species. Swiss then compared each query molecule against the ChEMBL library of 370,000 known active molecules and assigned a swiss score. This score was based on the similarity value with the most dissimilar and similar ligands, i.e., 0 (lowest score, no similarity) and 1 (maximum score, high similarity), were obtained. Afterward, the scores of the derivatives were clustered via ward distance and represented using a heatmap.

#### 4.4.2 Molecular Docking and Pharmacokinetic Evaluation

Human AChE (pdb id = 4BDT) crystallographic file was obtained from RCSB Protein Database website.^92^ The protein was prepared with Maestro’s Protein Preparation Wizard,^93^ and the gridbox was prepared via Receptor Grid Generation module of Maestro.^93^ The binding sites for coligands were employed for gridbox generation. PTZ ligands were drawn with 2D builder, minimized, and prepared via LigPrep module.^93^ Structural files for Huprine W (coligand of AChE) and Tacrine (coligand of BChE) were obtained from Drug Bank website,^94^ and for them, the same LigPrep process was implemented. After preparing all the necessary ligand files, Ligand Docking process of Glide program was initiated.^95^ pH was defined as the default 7.4 value. Precision was set to SP (Standard precision), and Ligand Sampling was set to Flexible. 10 poses were generated for each ligand, and the top ranked poses among them were evaluated. 2D-interaction diagrams were visualized via Ligand Interactions. This docking process was validated using the RMSD value between original and redocked Huprine W poses. This value was calculated as 0.871 Å, which indicates a valid docking process, since it should be below 2 Å. Moreover, molecular descriptors of these compounds were calculated via the QikProp module^96^ and were interpreted according to the QikProp manual.

#### 4.4.3 Pharmacophore Modeling

**Figure 12:**
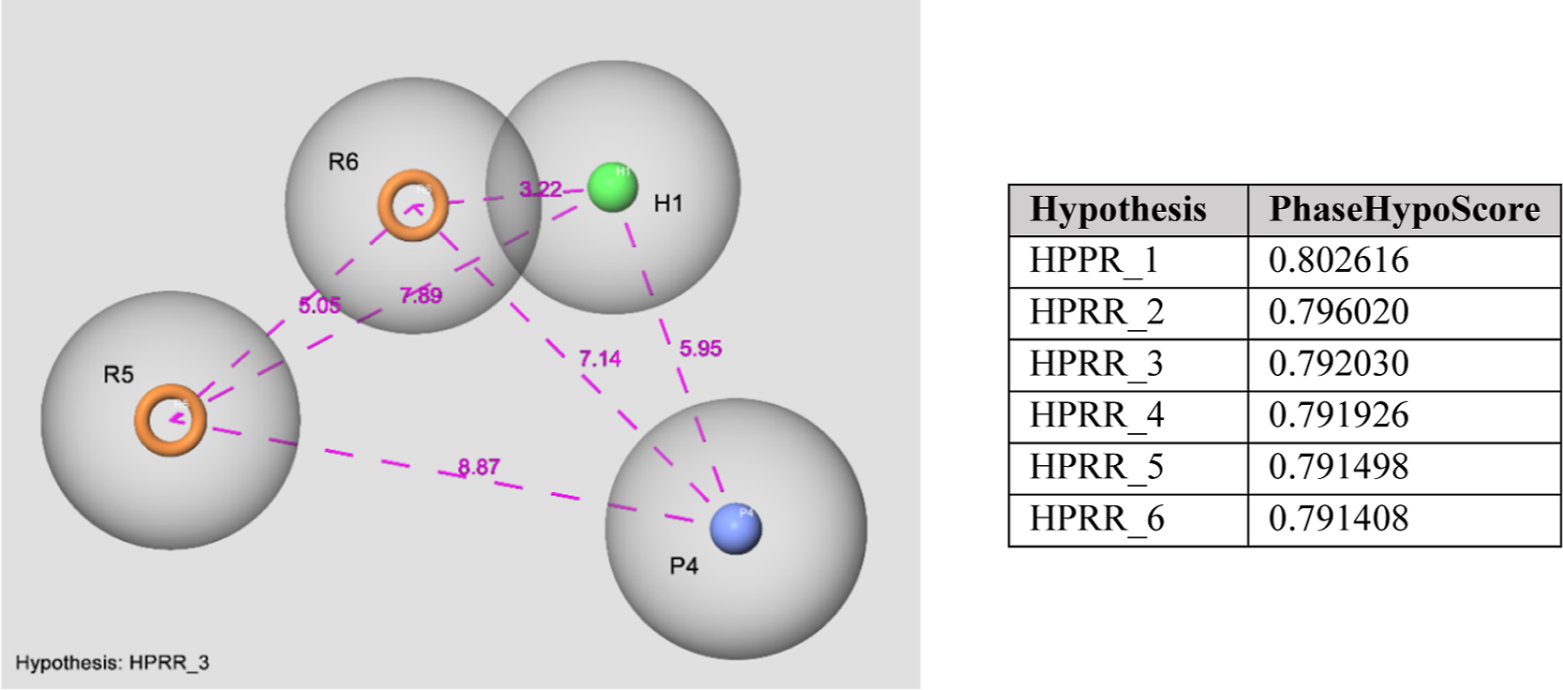
Pharmacophore hypothesis of HPPR_3.

Pharmacophore hypothesis for the synthesized PTZ derivatives were created via Develop Pharmacophore Hypothesis process of Phase module,^97^ and by doing so, these derivatives were tagged as active/inactive according to their IC_50_ values. Suitability of these ligands to this authentic model was evaluated using the Ligand and Database Screening module. In this model, fitting the ligands to the hypothesis can be determined with PhaseScreenScore. This score was defined as 3.00 for the standard, and the scoring of other compounds was done accordingly. In the created pharmacophore hypothesis: two aromatic ring features (R5, R6), one hydrophobic feature (H1), and one positive cation feature (P4) were determined (Figure 12). The PhaseHypoScore for this hypothesis was 0.792030. The compounds in the set were evaluated using this authentic pharmacophore hypothesis. Table 3 represents the data set that was used to create the pharmacophore hypothesis, as presented in Figure 12.

**Table 3:**
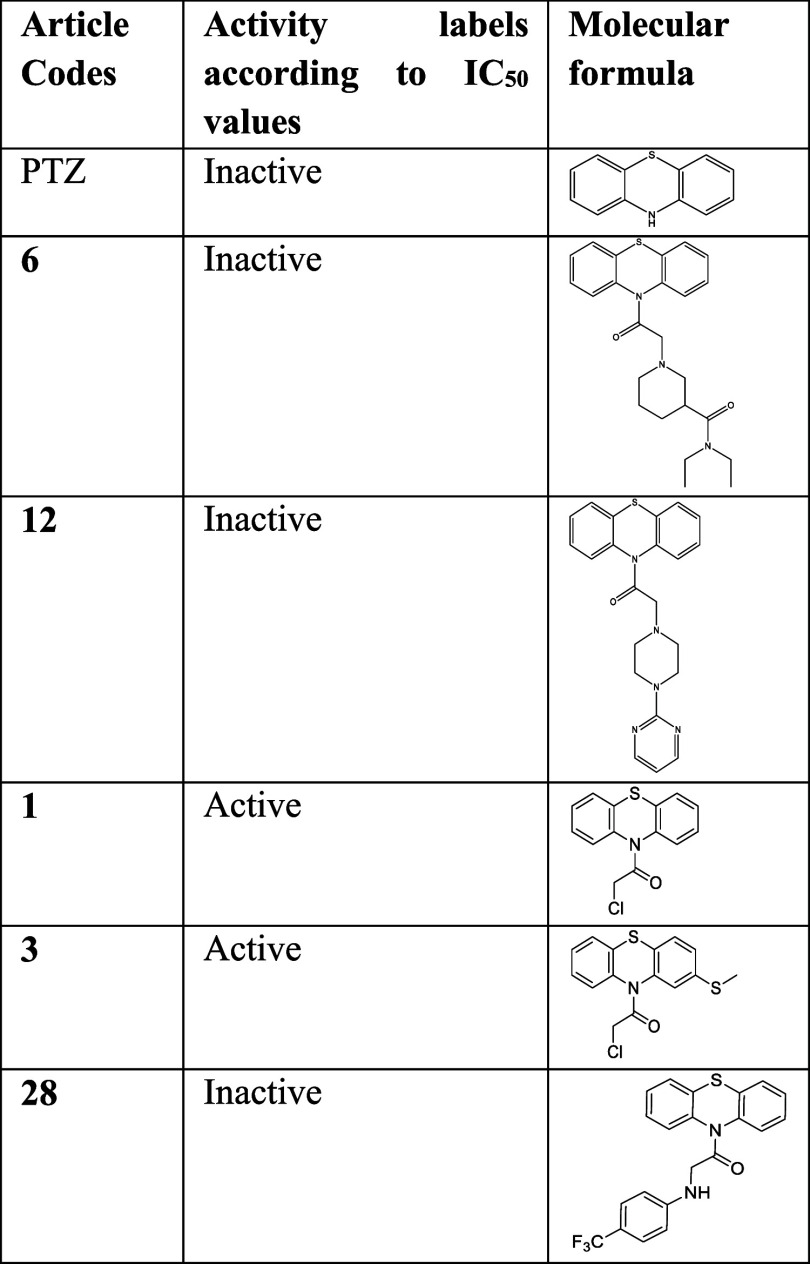
Data Set for the Created Pharmacophore Model

## Supporting Information Available

Spectral data (^1^H NMR and ^13^C NMR), in vitro Hep3B and SkHep1 IC_50_ data, in vitro cholinesterase activity studies, in vivo zebrafish toxicity profiles, target identification plot, and docking and pharmacophore analysis results (PDF)

## Abbreviations

PTZ: phenothiazine
TFP: trifluoperazine
PCP: prochlorperazine
PPH: perphenazine
SFB: sorafenib
ACh: acetylcholine
AchE: acetylcholinesterase
BChE: butyrylcholinesterase
HUW: Huprin W
HCC: hepatocellular carcinoma
rt: room temperature
