# Supplementary material for: Synthesis and Structure of Novel Phenothiazine Derivatives, and Compound Prioritization via In Silico Target Search and Screening for Cytotoxic and Cholinesterase Modulatory Activities in Liver Cancer Cells and In Vivo in Zebrafish
Source: ACS Omega. 2024 Jul 3;9(28):30594–614. doi: 10.1021/acsomega.3c06532 (PMC11256110; doi:10.1021/acsomega.3c06532)
Supplement: Supplementary file 1 — ao3c06532_si_001.pdf [file ao3c06532_si_001.pdf]

Supporting Information

Synthesis and structure of novel phenothiazine derivatives, and compound prioritization via *in silico* target search and screening for cytotoxic and cholinesterase modulatory activities in liver cancer cells and *in vivo* in zebrafish

Mehmet Murat Kisla[#1,2], Murat Yaman[#3], Fikriye Zengin-Karadayi[1], Busra Korkmaz[4], Omer Bayazeid[4], Amrish Kumar[5], Ravindra Peravali[5], Damla Gunes[3], Rafed Said Tiryaki[4], Emine Gelinci[6], Gulcin Cakan-Akdogan[6,7], Zeynep Ates-Alagoz[*1], Ozlen Konu[*3,4,8]

[1]*Department of Pharmaceutical Chemistry, Faculty of Pharmacy, Ankara University, 06100, Ankara, Turkey*

[2]*Graduate School of Health Sciences, Ankara University, 06100, Ankara, Turkey*

[3]*Interdisciplinary Program in Neuroscience, Bilkent University, 06800, Ankara, Turkey*

[4]*Department of Molecular Biology and Genetics, Bilkent University, 06800, Ankara, Turkey*

[5]*Institute of Toxicology and Genetics (ITG), Karlsruhe Institute of Technology (KIT), 76344, Eggenstein-Leopoldshafen, Germany*

[6]*Izmir Biomedicine and Genome Center (IBG), 35340, Izmir, Turkey*

[7]*Medical Biology Department, Dokuz Eylul University, 35340, Izmir, Turkey*

[8]*UNAM-Institute of Materials Science and Nanotechnology, Bilkent University, 06800, Ankara, Turkey*

***Corresponding Authors**

Ozlen Konu, E-mail: konu@fen.bilkent.edu.tr

Zeynep Ates-Alagoz, E-mail: zeynep.ates@pharmacy.ankara.edu.tr

[#]Co-first Authors

CONTENT

1. Derivative Response Comparisons in Hep3B and SkHep1 Cells Based on IC50 Values…………………...……...S2
2. SwissTargetPrediction Plot……………………………………………………………….…………………....S3
3. Changes in Cholinesterase Activity in Hep3B and SkHep1 Cells After Treatment with Compounds 8, 9 and 10……………………………………………………………………………………..……....……..S4
4. Modulation of BChE Expression Levels in SkHep1 Cells As as Result of Compound Exposure…………..……....S4
5. Heatmap Representations of AChE and BChE Expression and Copy Number in Liver Cancer Cell Lines……......S5
6. Zebrafish Toxicity Profiles for Compound 4 and 10……………………………………………………….S5-S6
7. Total Length Measurements on Zebrafish Larvae after Exposure to Compounds…………………….…………….S7
8. NMR Spectrums of the Phenothiazine Derivatives……………………………………………………………..S8-S31
9. Glide Scores of Phenothiazine Derivatives with AChE and BChE……………………………………….....S32-S34
10. Pharmacophore Screening Based on HPRR_3 Pharmacophore Hypothesis…………………...…………………..….S35

Current Affiliation for Murat Yaman: Department of Pediatrics, University of Michigan, 48109, Ann Arbor, MI, USA

Derivative Response Comparisons in Hep3B and SkHep1 Cells Based on IC50 Values

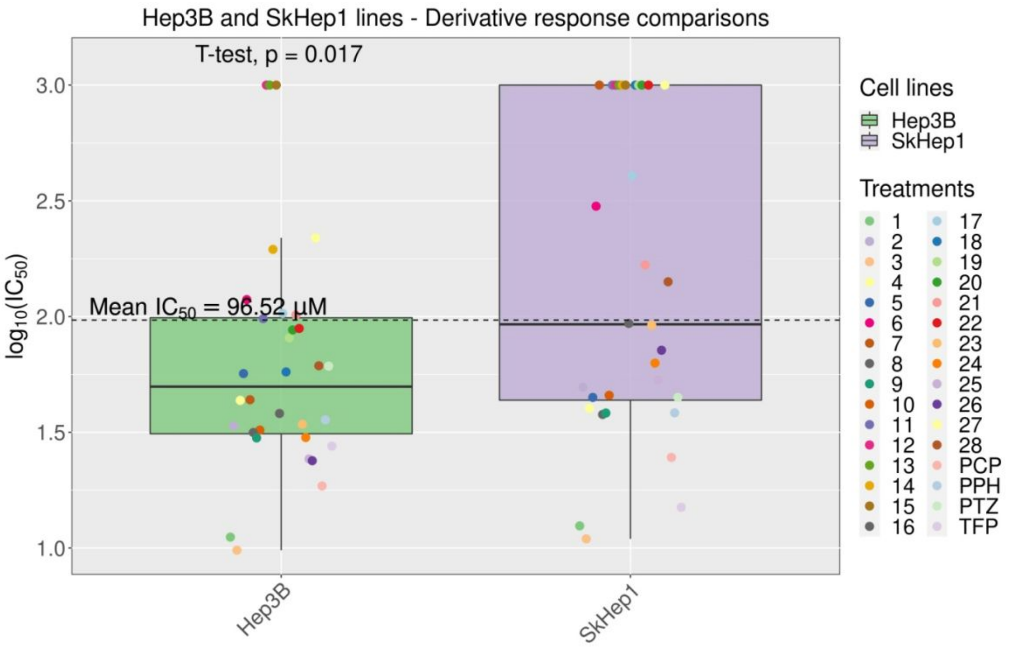

Figure S1. T-test analyses on the log scaled IC_{50} values for the derivative exposures on Hep3B and SkHep1.

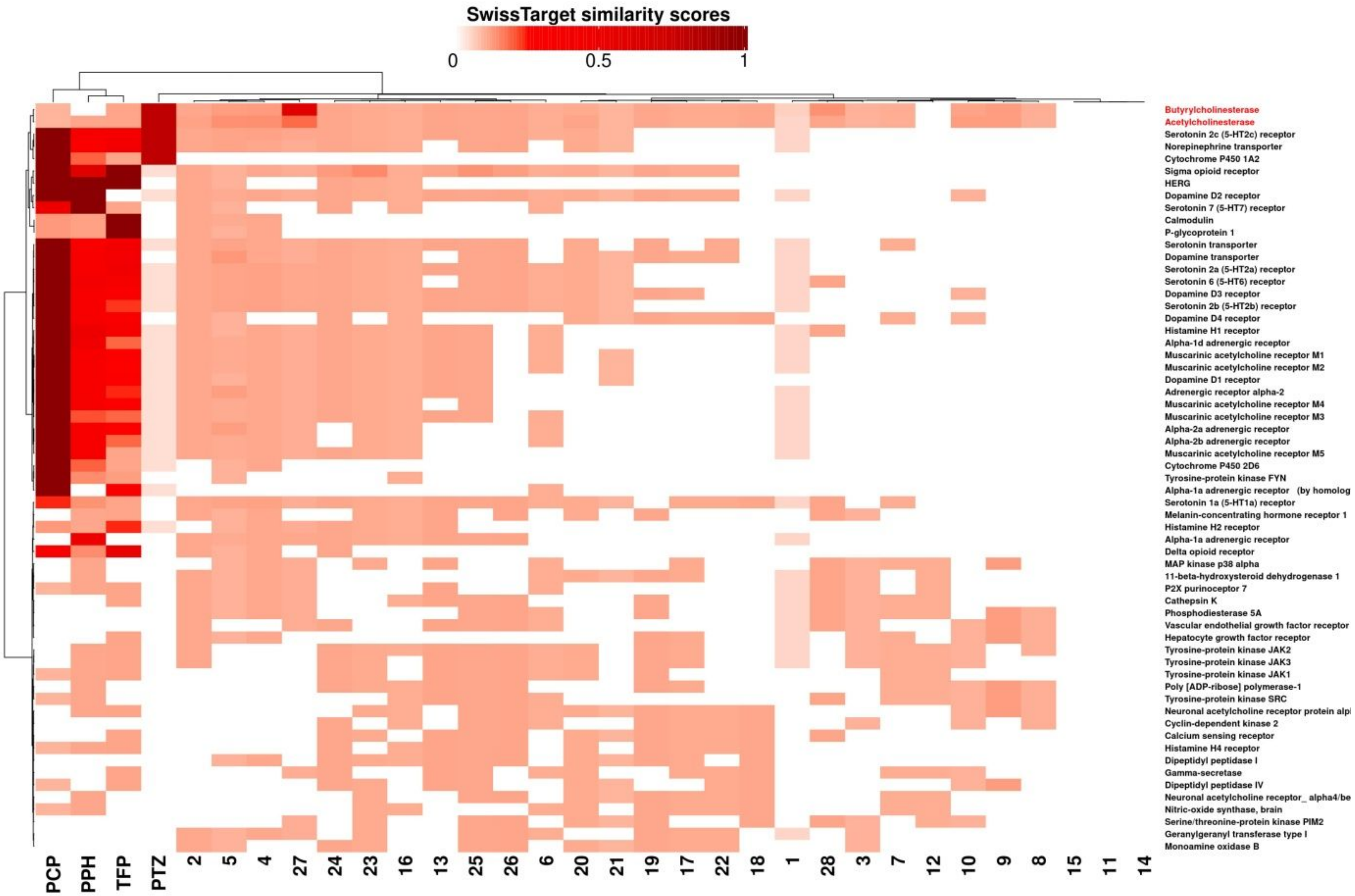

Figure S2. Possible targets of the derivatives according to the SwissTargetPrediction algorithm. The results are depicted on a heatmap where darker tones indicate more likelihood for derivative-target protein interactions.

Changes in Cholinesterase Activity in Hep3B and SkHep1 Cells After Treatment with Compounds 8, 9 and 10

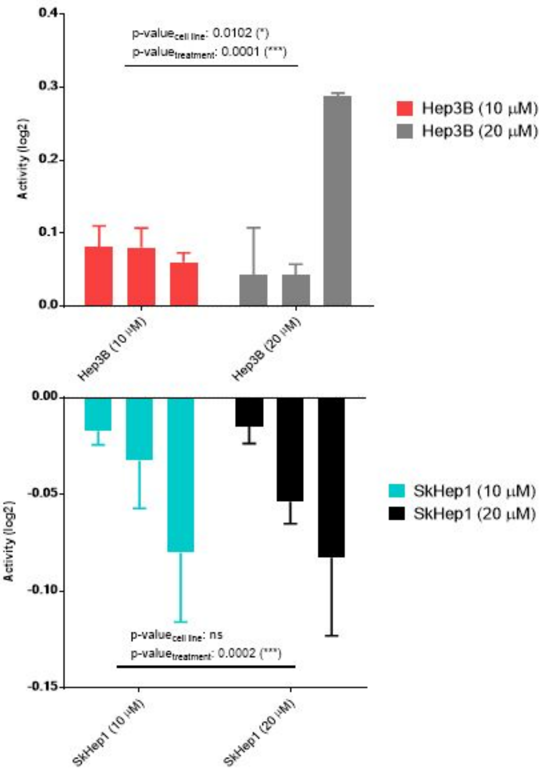

Figure S3. Plots of cholinesterase activity levels after 24-hour exposures. Hep3B and SkHep1 two-way ANOVA dose comparisons between 10 µM and 20 µM (p-value$_{\text{cell line}}$) for novel derivatives **8**, **9** and **10** (p-value$_{\text{treatments}}$), in order.

Modulation of BChE Expression Levels in SkHep1 Cells As as Result of Compound Exposure

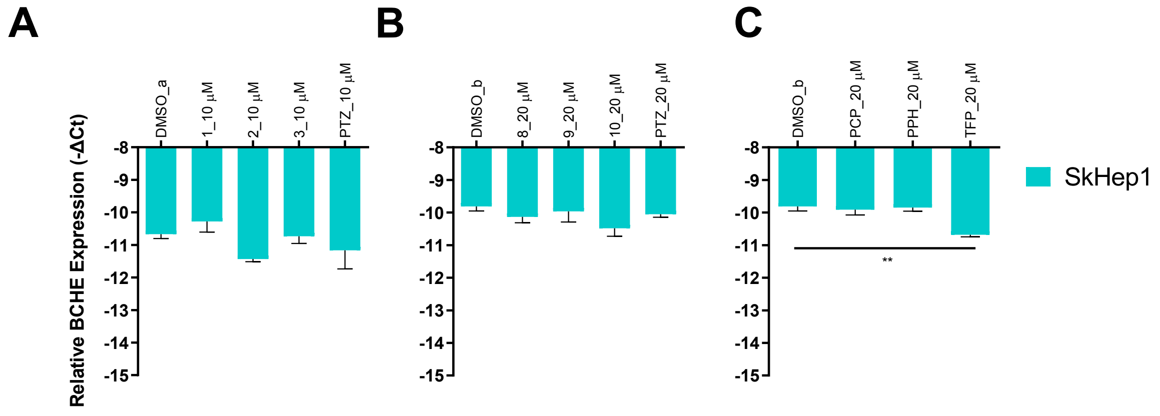

Figure S4. The expression of BCHE in SkHep1 cells after treatment with A) **1**, **2**, **3**, PTZ at 10 µM; B) **8**, **9**, **10**, PTZ at 20 µM; and C) PCP, PPH, TFP at 20 µM for 24 hours. While y-axis shows relative BCHE expression to TPT1 reference gene as -DeltaCt, One-way ANOVA followed by Dunnett's tests were used to compare each treatment group to a batch and cell-line specific DMSO control group, indicated as DMSO_a-b (*: p≤0.05, **: p≤0.01, ***: p≤0.001, ****: p≤0.0001, #: p≤0.1).

Heatmap Representations of AChE and BChE Expression and Copy Number in Liver Cancer Cell Lines

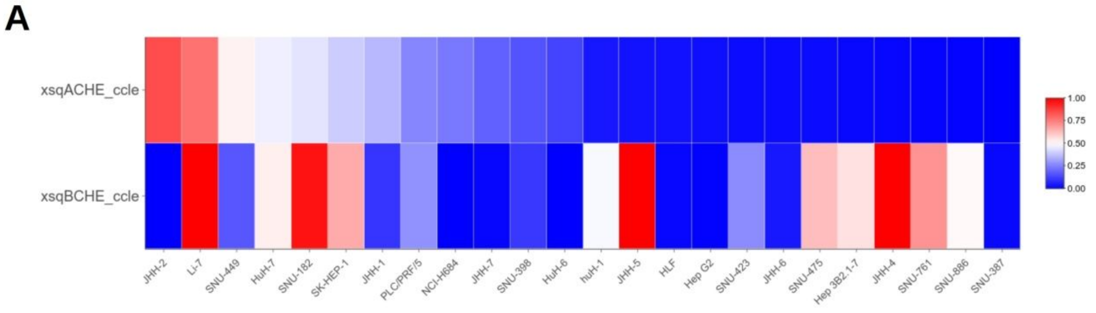

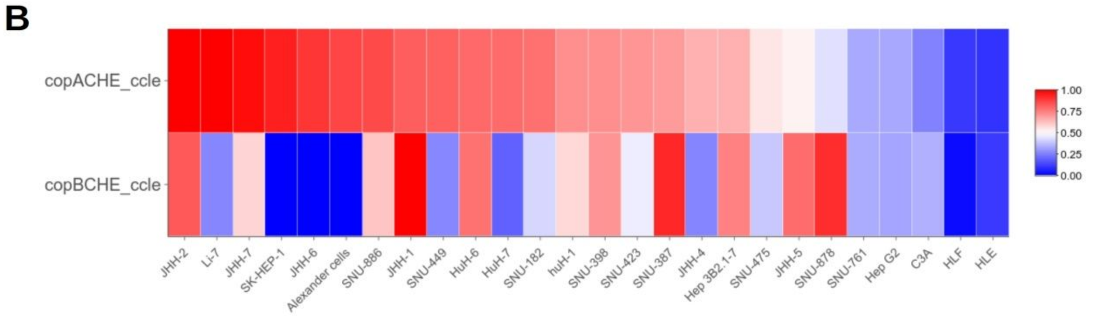

Figure S5. Heatmap representations for AChE and BChE across liver cancer lines, (A) log2 RPKM+1 normalized CCLE-Broad-MIT xsq: RNA-seq data, (B) CCLE-Broad-MIT cop: DNA copy number. The analyses are retrieved from CellMinerCDB Version 1.2 (September 2020 Release)

Zebrafish Toxicity Profiles for Compound 4 and 10

4

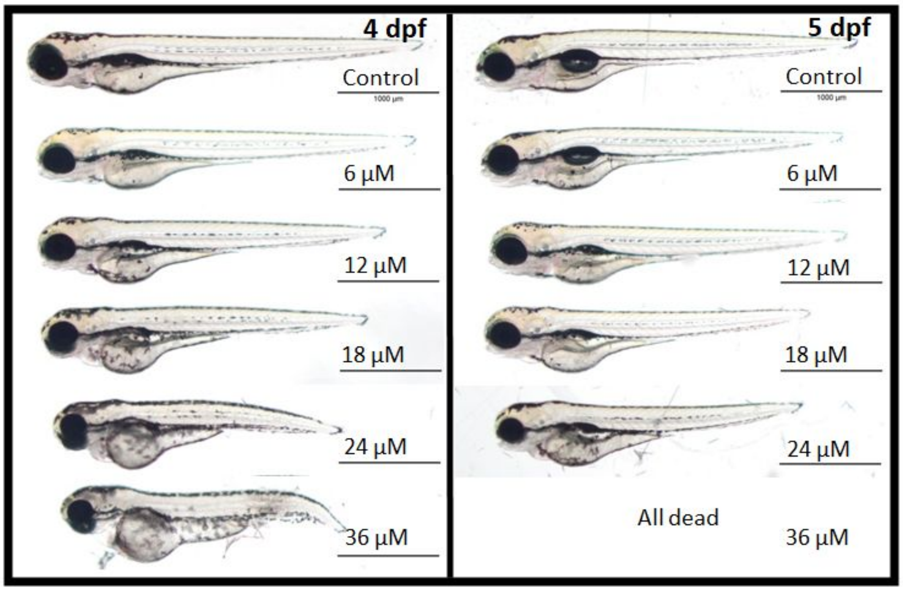

Figure S6. Representative images of 4 and 5 dpf larvae after 48 and 72 hours of exposure, respectively, to compound **4**.

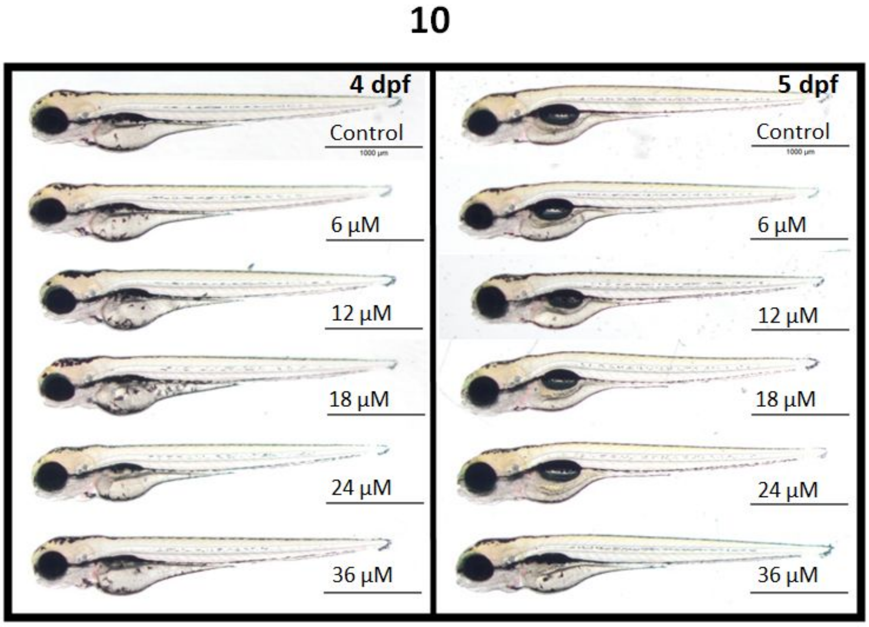

Figure S7. Representative images of 4 and 5 dpf larvae after 48 and 72 hours of exposure, respectively, to compound **10**.

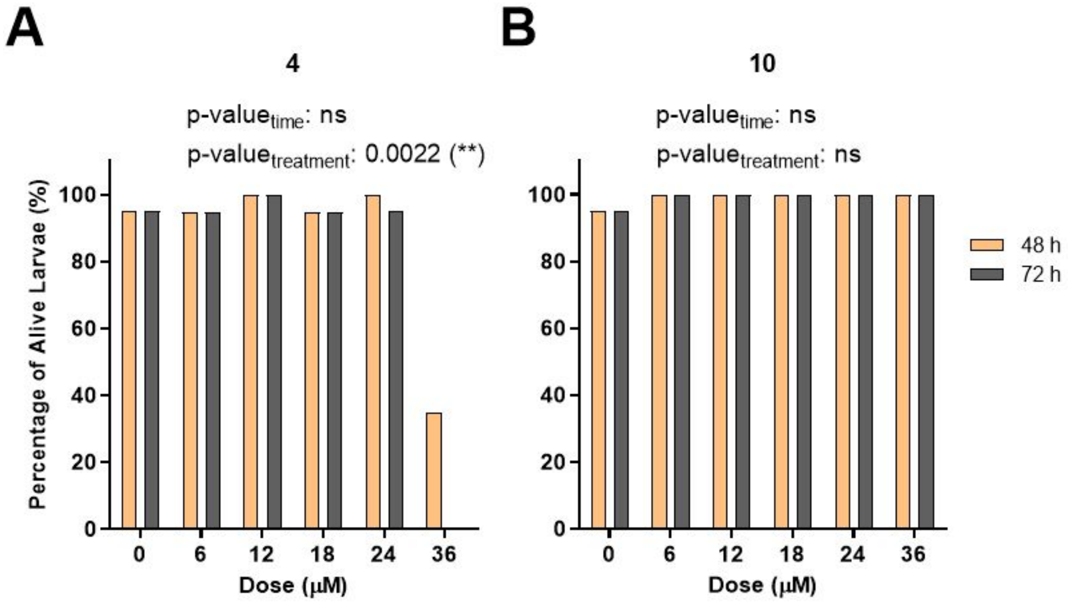

Figure S8. The percentage of larvae alive after treatment with different concentrations of compounds A) **4** and B) **10** for 48 and 72 hours starting from 2 dpf. The statistical analysis was performed using two-way ANOVA (*: p≤0.05, **: p≤0.01, ***: p≤0.001, ****: p≤0.0001).

Total Length Measurements on Zebrafish Larvae after Exposure to Compounds

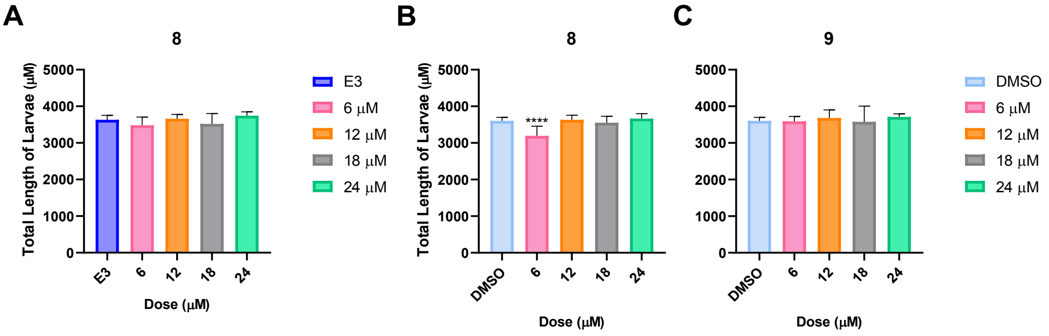

Figure S9. The total length of A) 4 dpf larvae after being exposed to compound 8 for 48 hours and 5 dpf larvae after being exposed to compound B) **8** and C) **9** for 72 hours starting from 2 dpf. The statistical analysis was performed using one-way ANOVA/Dunnett's test (*: p≤0.05, **: p≤0.01, ***: p≤0.001, ****: p≤0.0001).

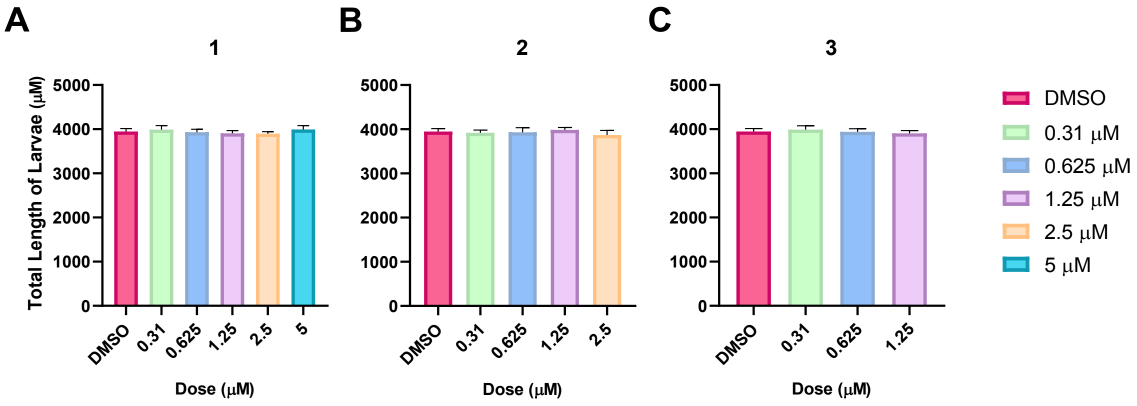

Figure S10. The total length of 5 dpf larvae after being exposed to intermediate phenothiazines A) **1**, B) **2** and C) **3** for 72 hours starting from 2 dpf. The statistical analysis was performed using one-way ANOVA/Dunnett's test (*: p≤0.05, **: p≤0.01, ***: p≤0.001, ****: p≤0.0001).

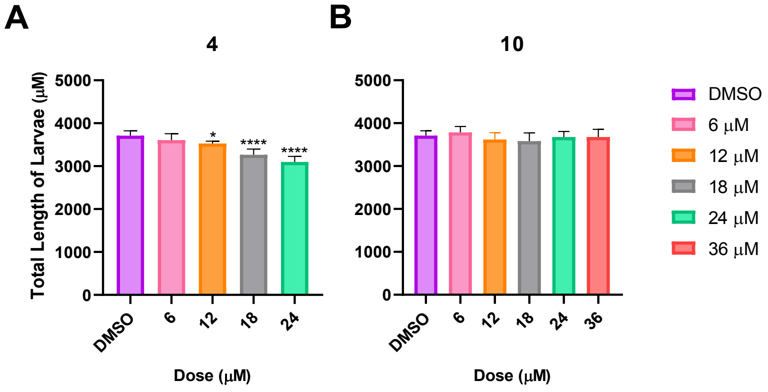

Figure S11. The total length of 5 dpf larvae after being exposed to compound A) **4** and B) **10** for 72 hours starting from 2 dpf. The statistical analysis was performed using one-way ANOVA/Dunnett's test (*: p≤0.05, **: p≤0.01, ***: p≤0.001, ****: p≤0.0001).

NMR Spectrums of the Phenothiazine Derivatives

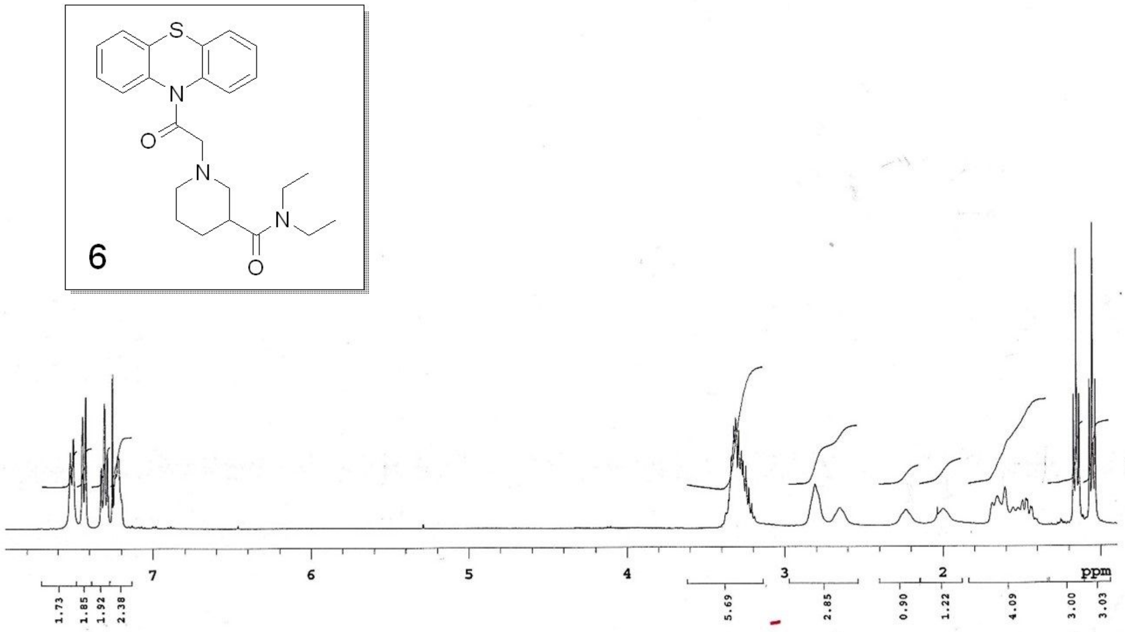

Figure S12. ^{1}H NMR spectrum of compound **6**.

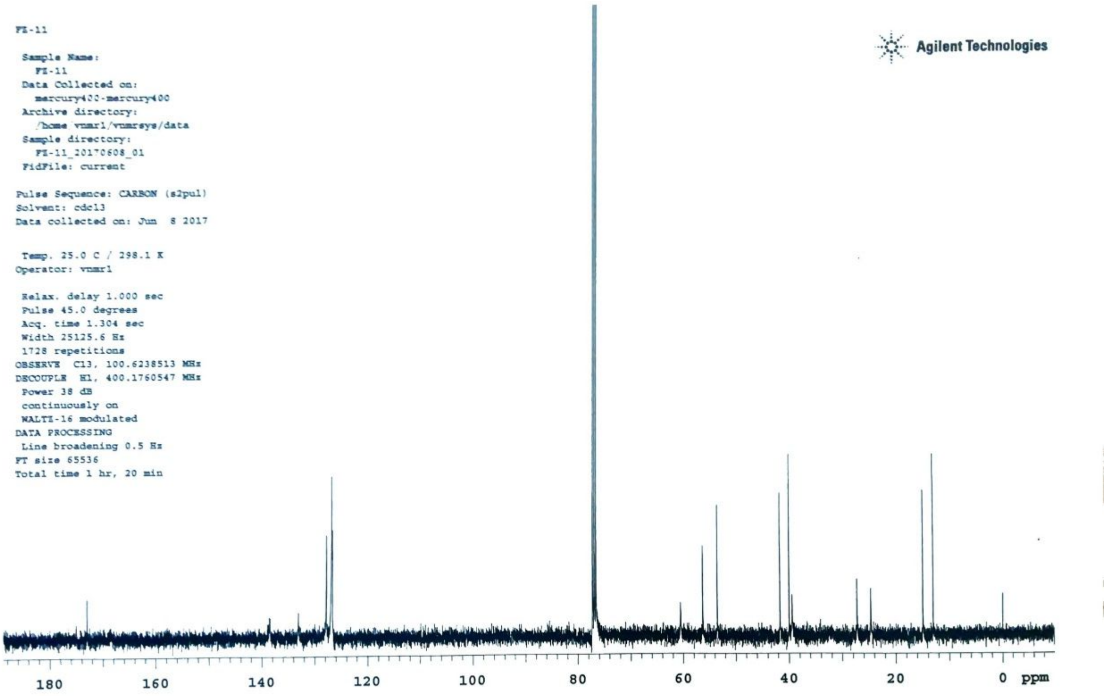

Figure S13. ^{13}C NMR spectrum of compound **6**.

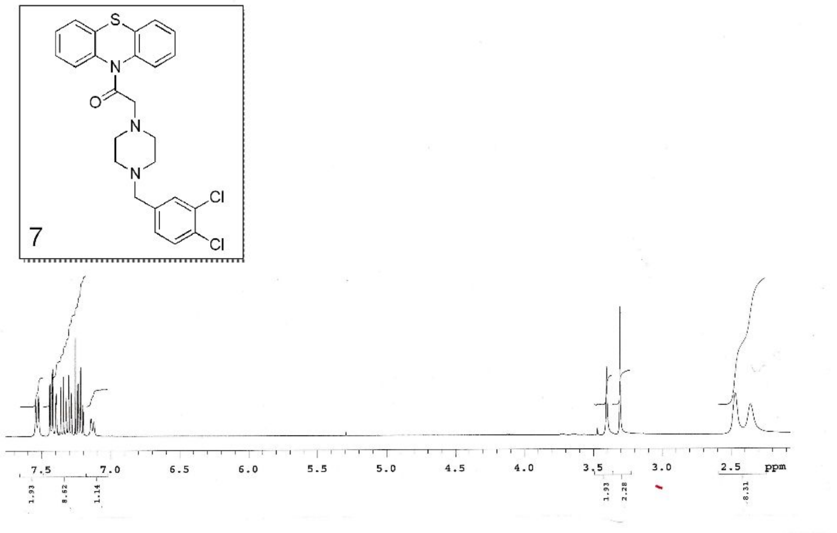

Figure S14. ^{1}H NMR spectrum of compound **7**.

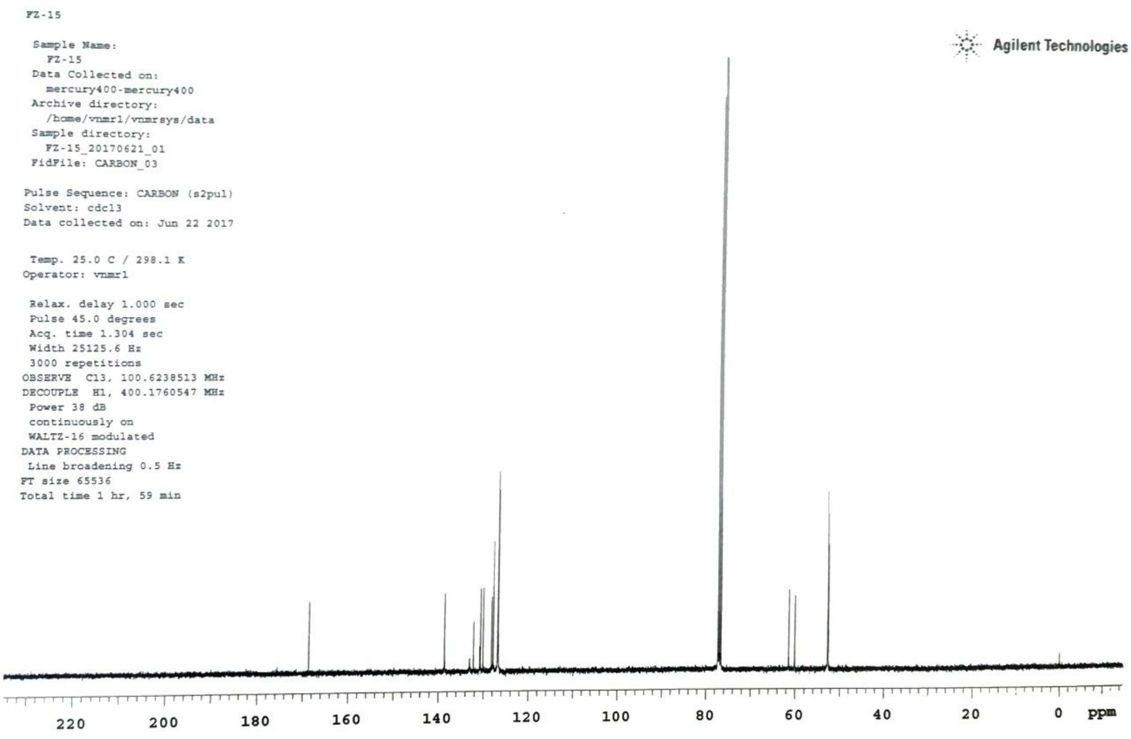

Figure S15. ^{13}C NMR spectrum of compound **7**.

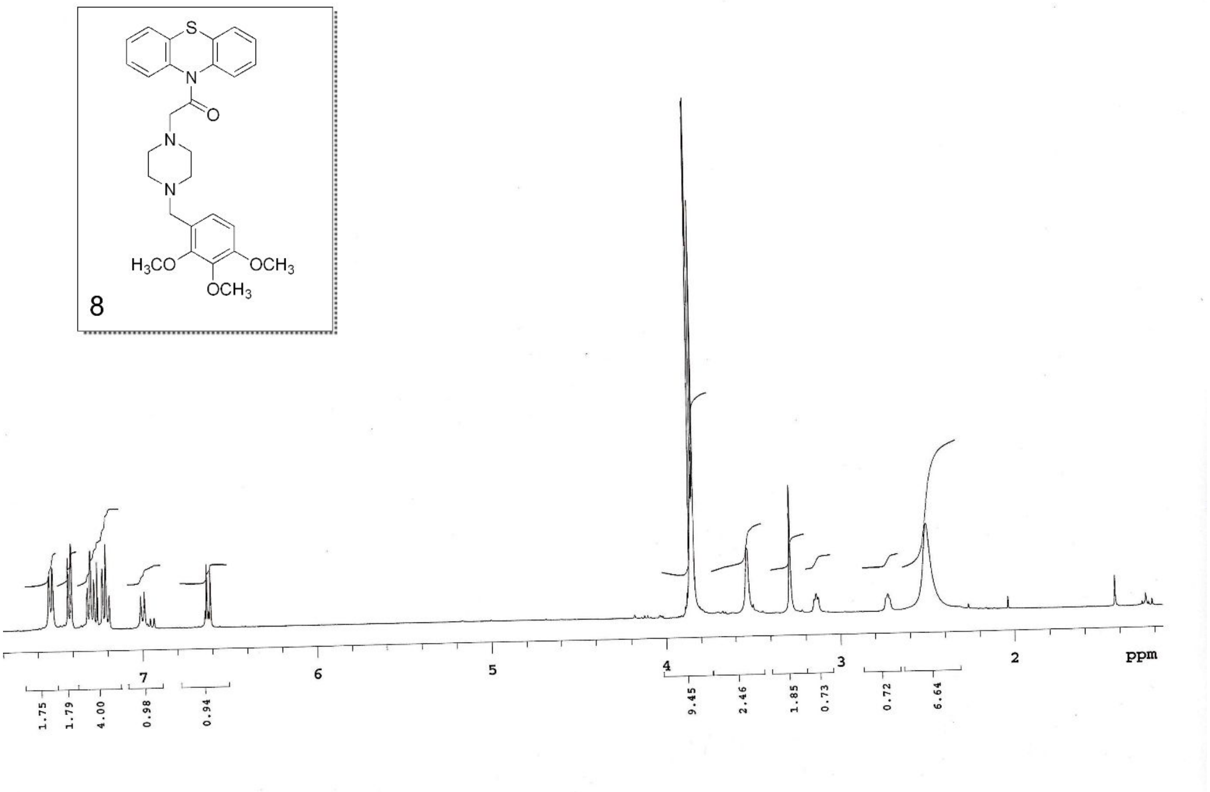

Figure S16. ^{1}H NMR spectrum of compound **8**.

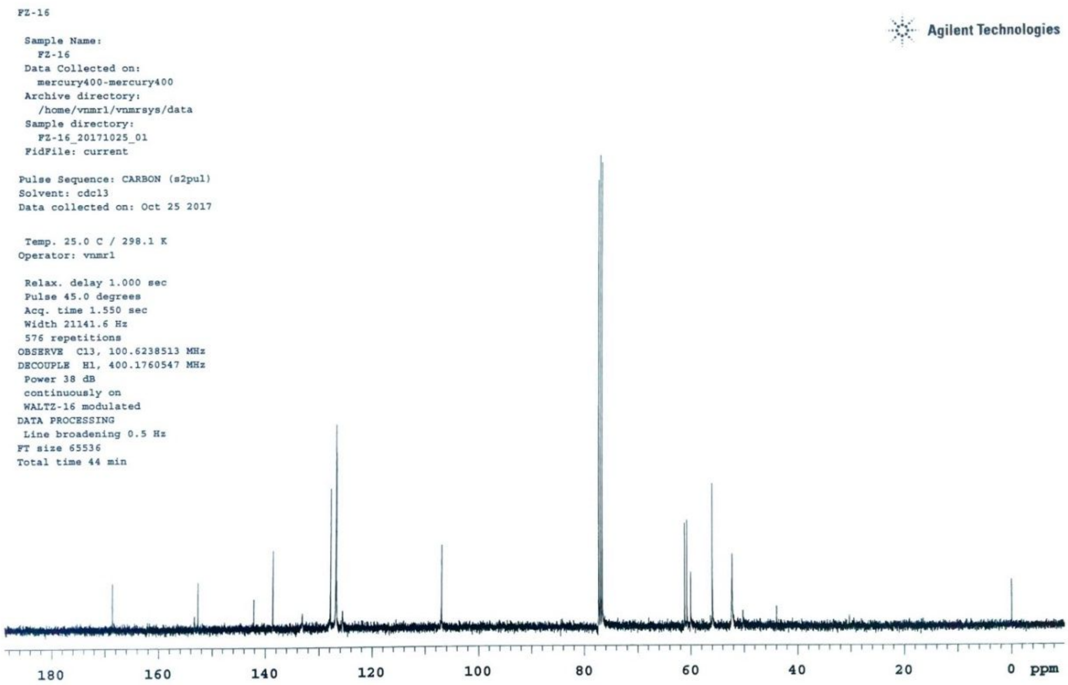

Figure S17. ^{13}C NMR spectrum of compound **8**.

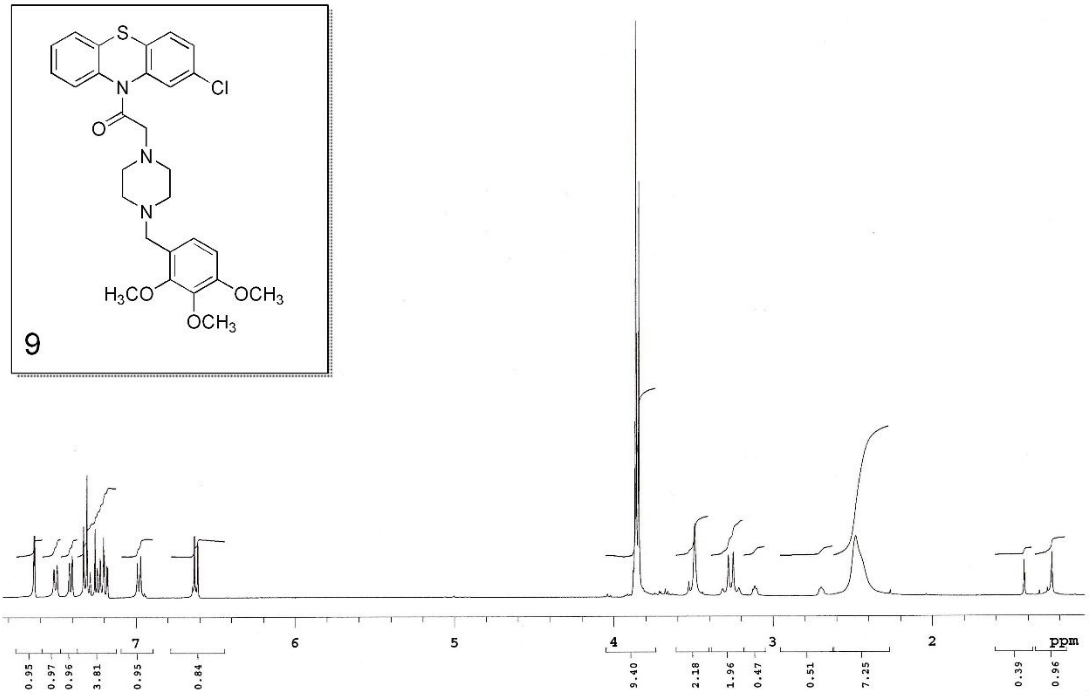

Figure S18. ^{1}H NMR spectrum of compound **9**.

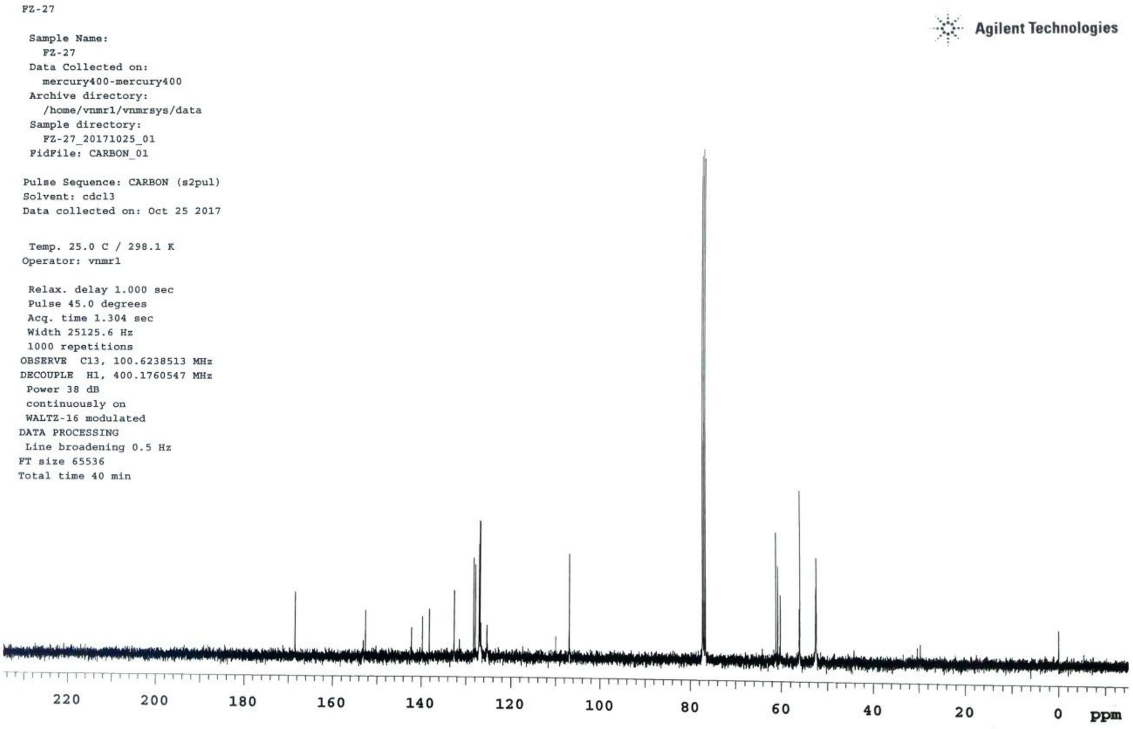

Figure S19. ^{13}C NMR spectrum of compound **9**.

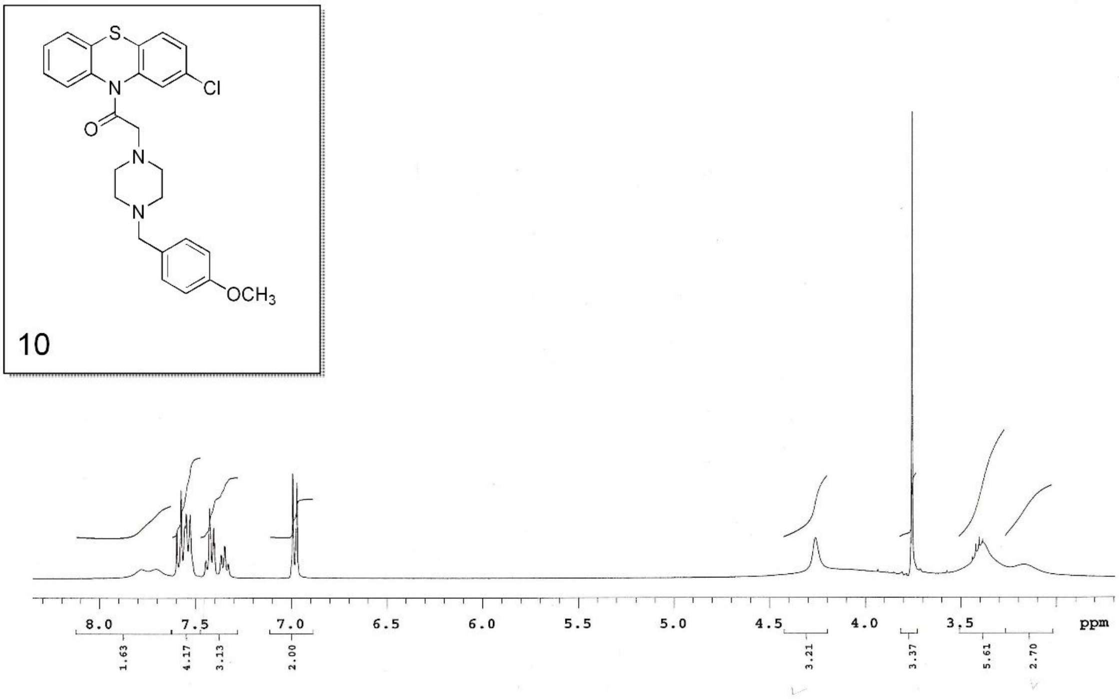

Figure S20. ^{1}H NMR spectrum of compound **10**.

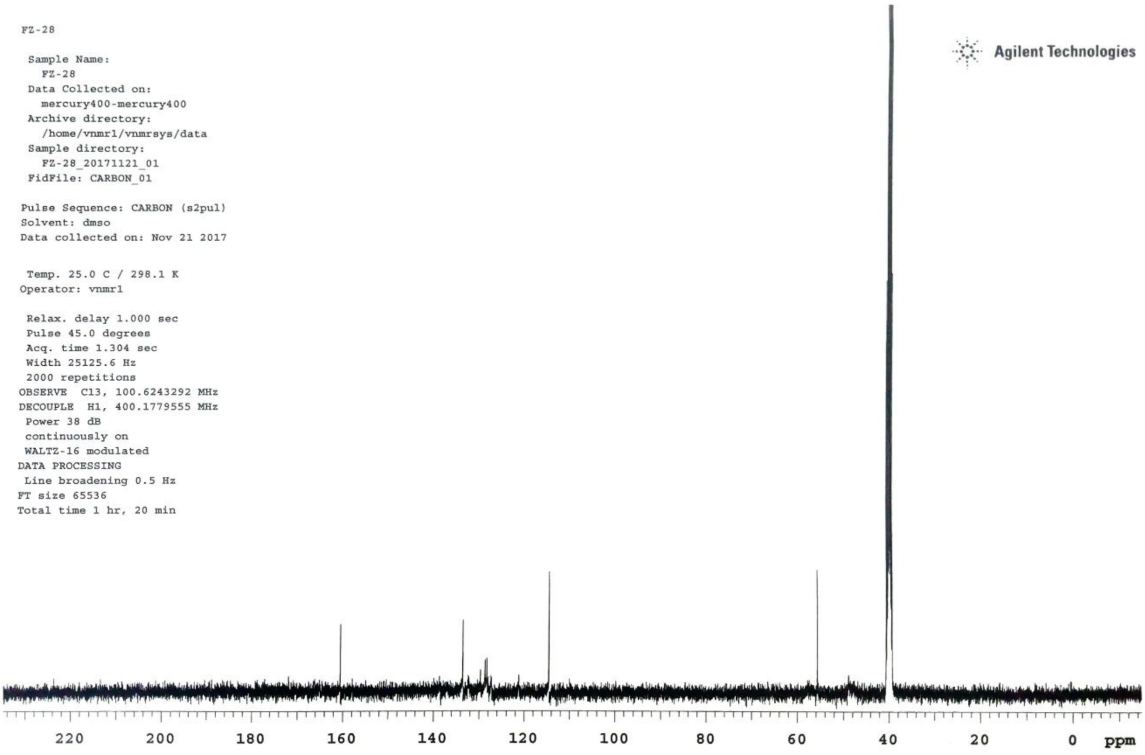

Figure S21. ^{13}C NMR spectrum of compound **10**.

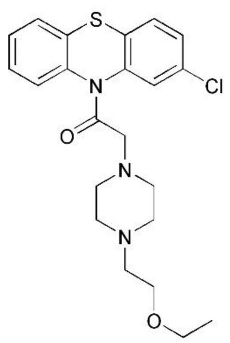

11

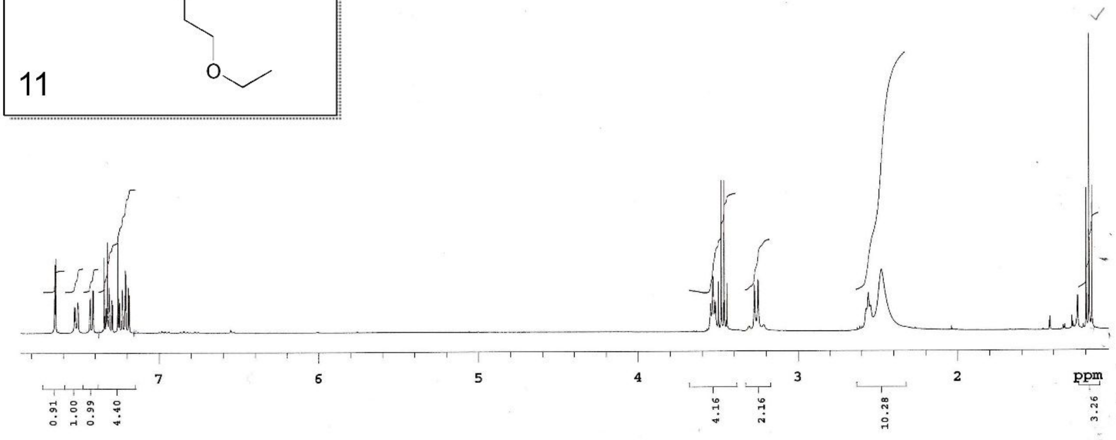

Figure S22. ^{1}H NMR spectrum of compound **11**.

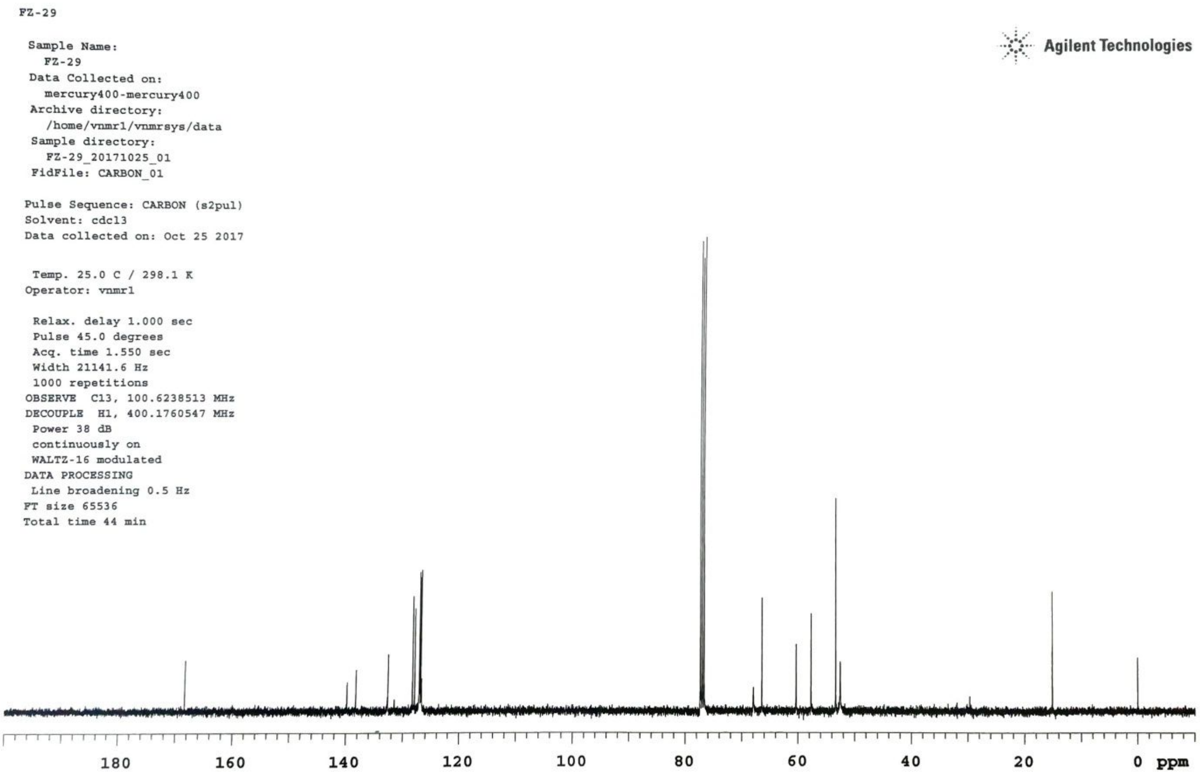

Figure S23. ^{13}C NMR spectrum of compound **11**.

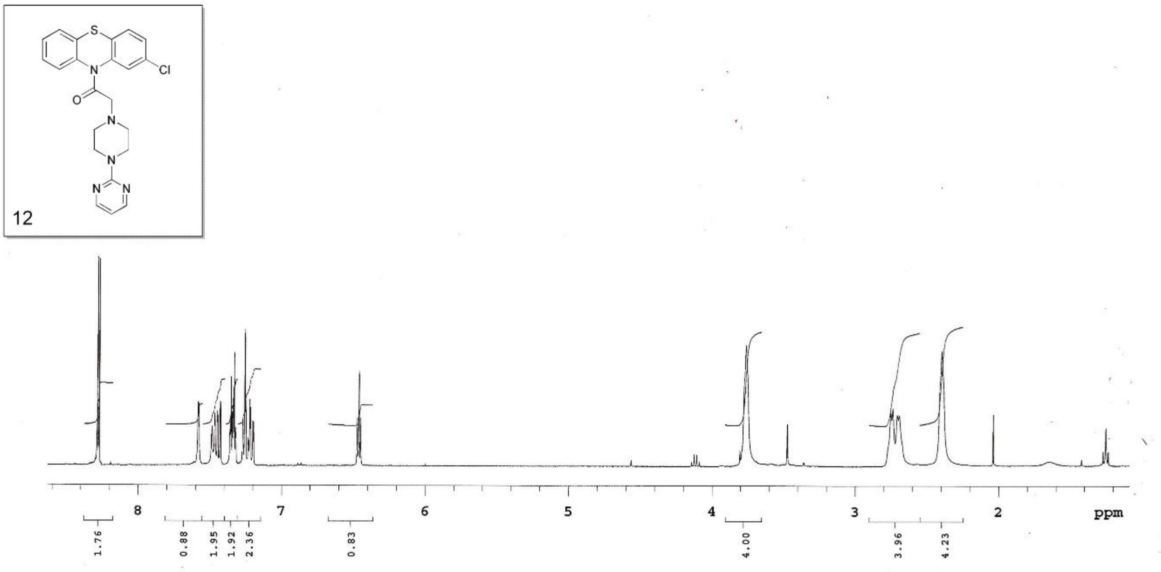

Figure S24. ^{1}H NMR spectrum of compound **12**.

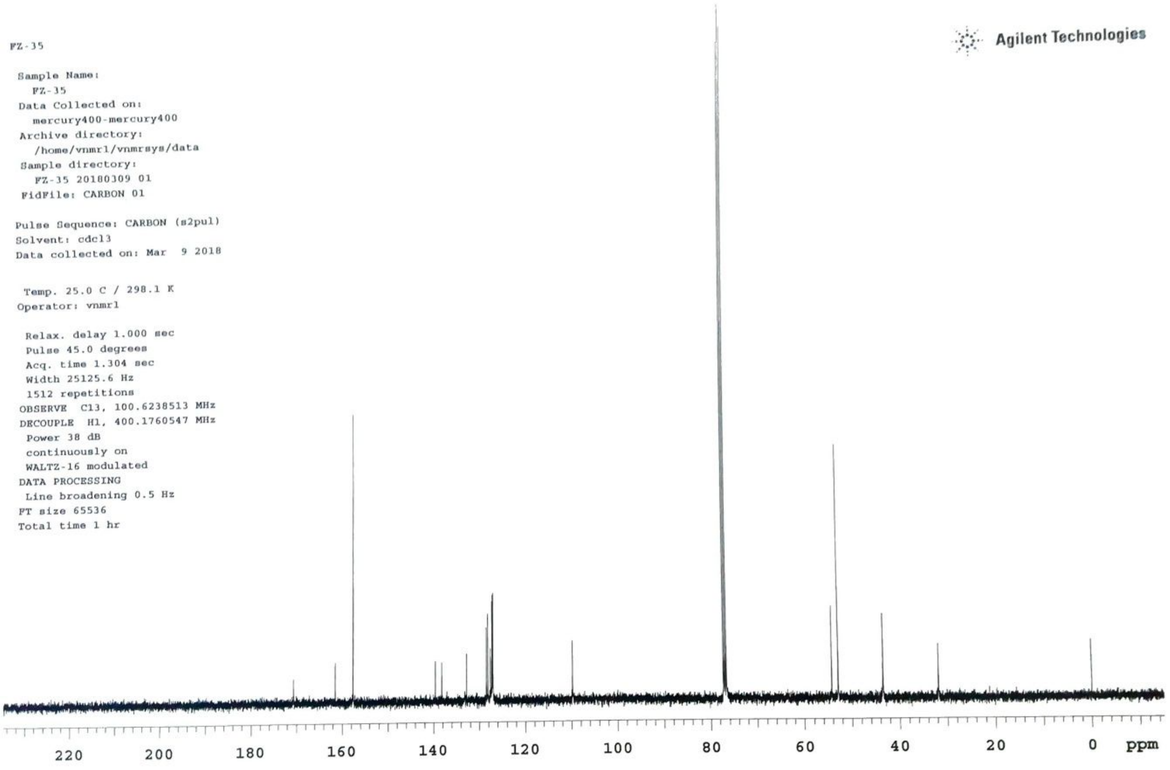

Figure S25. ^{13}C NMR spectrum of compound **12**.

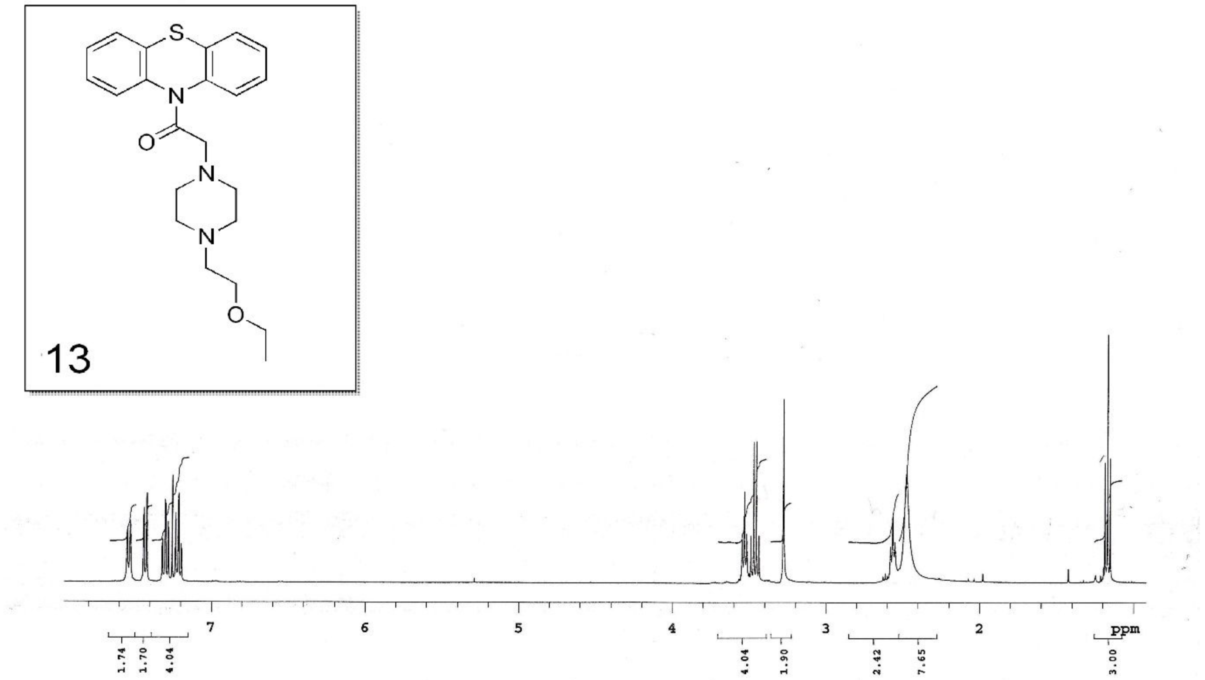

Figure S26. ^{1}H NMR spectrum of compound **13**.

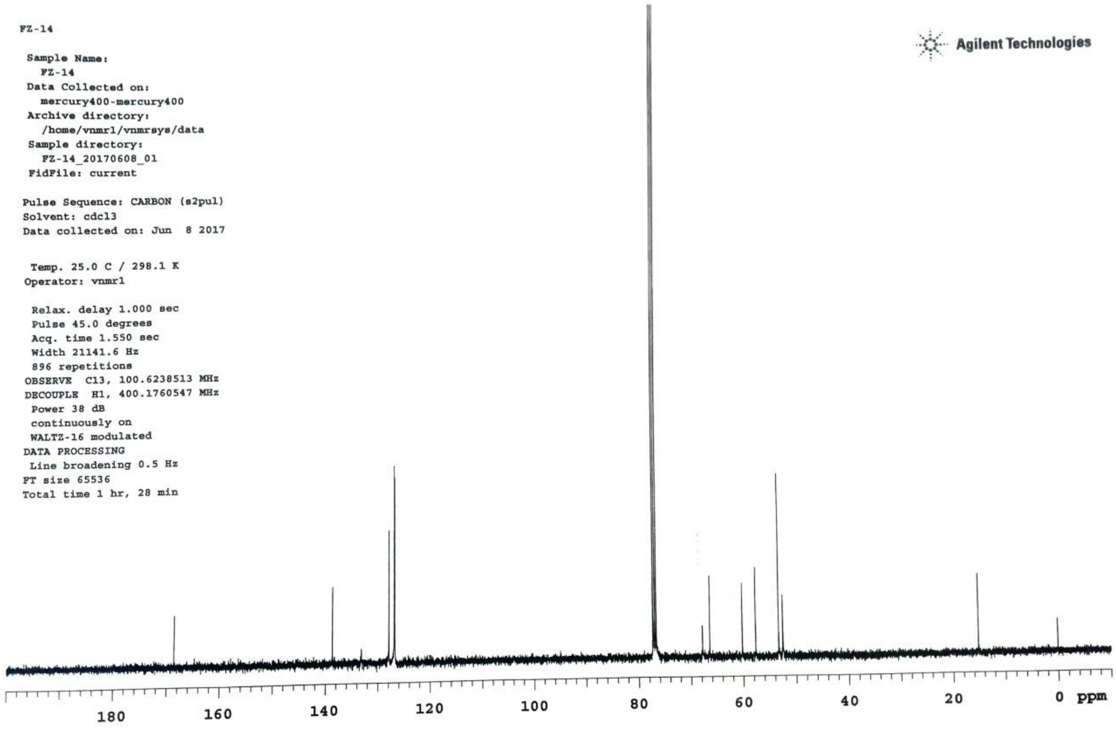

Figure S27. ^{13}C NMR spectrum of compound **13**.

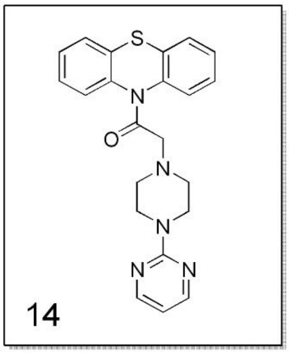

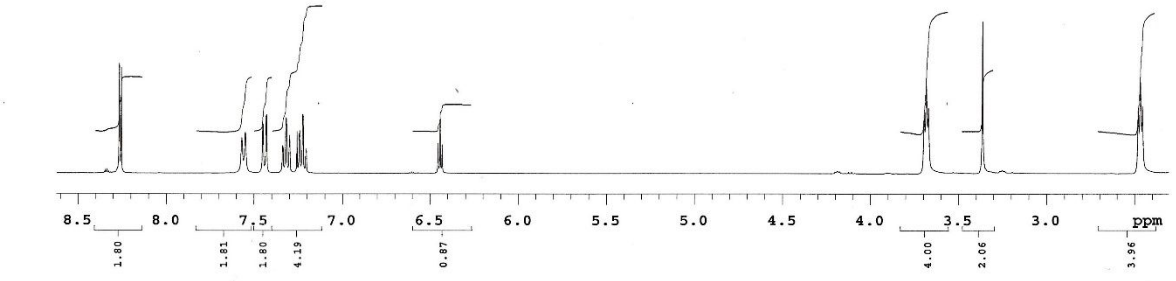

Figure S28. ^{1}H NMR spectrum of compound **14**.

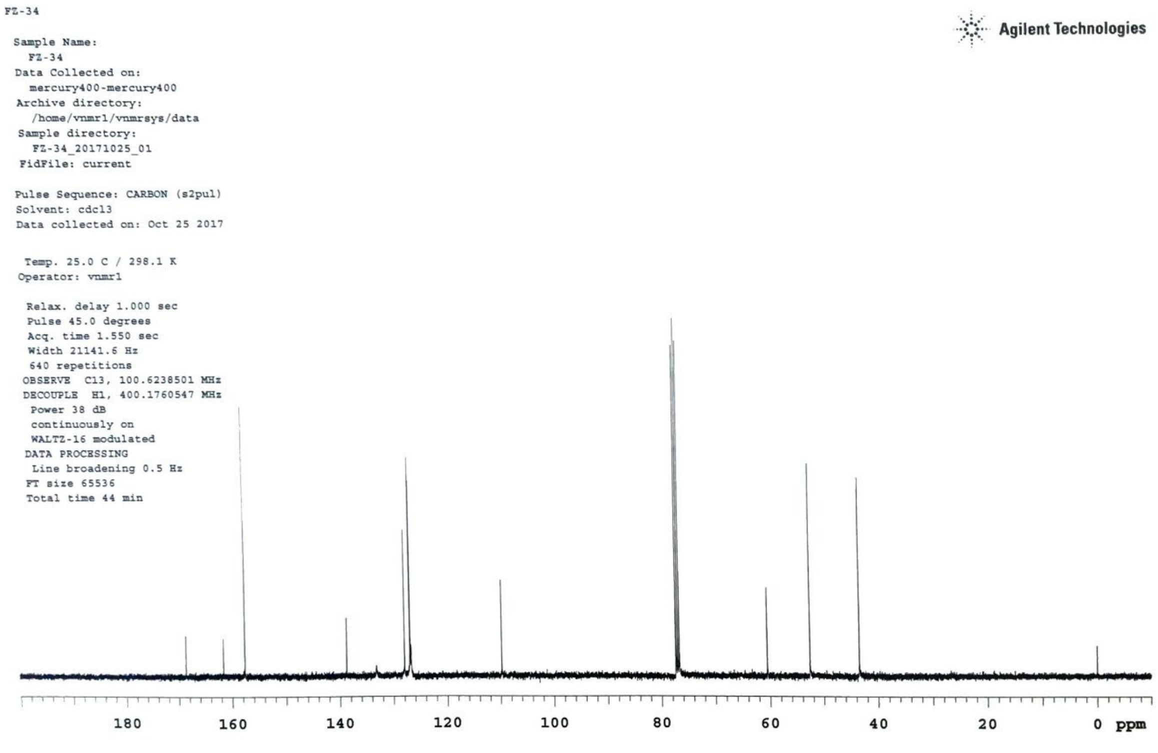

Figure S29. ^{13}C NMR spectrum of compound **14**.

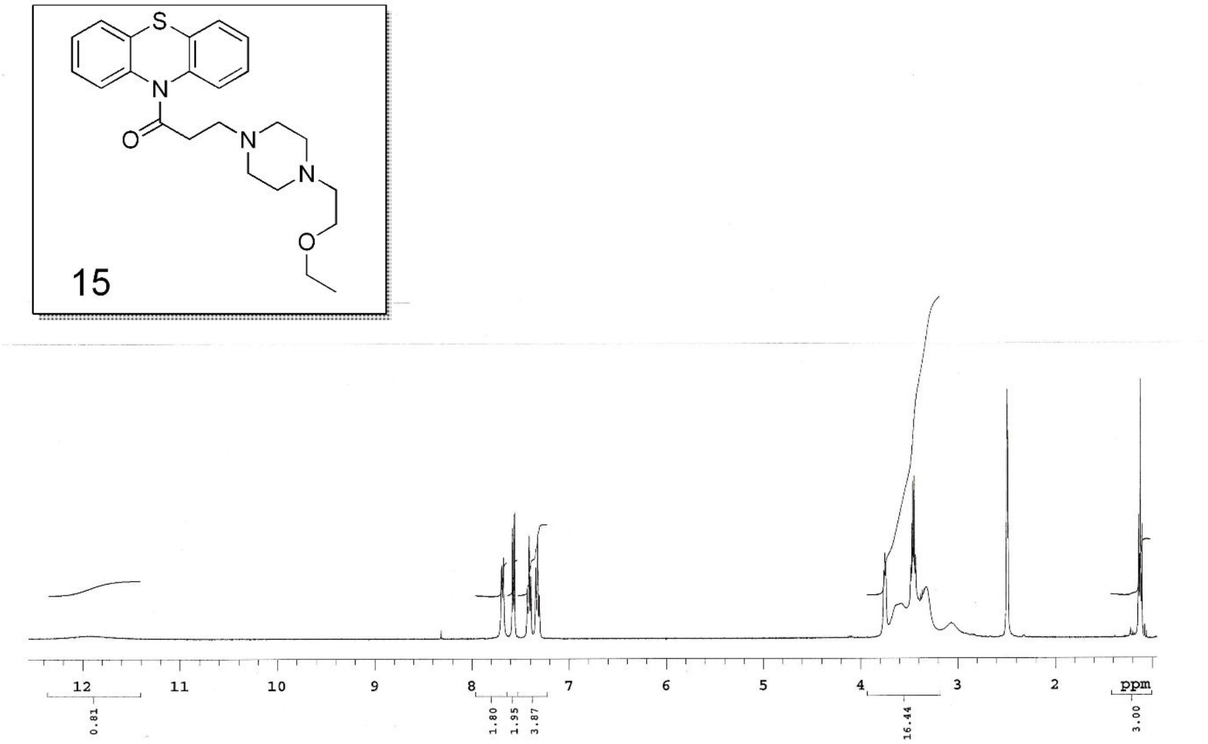

Figure S30. ^{1}H NMR spectrum of compound **15**.

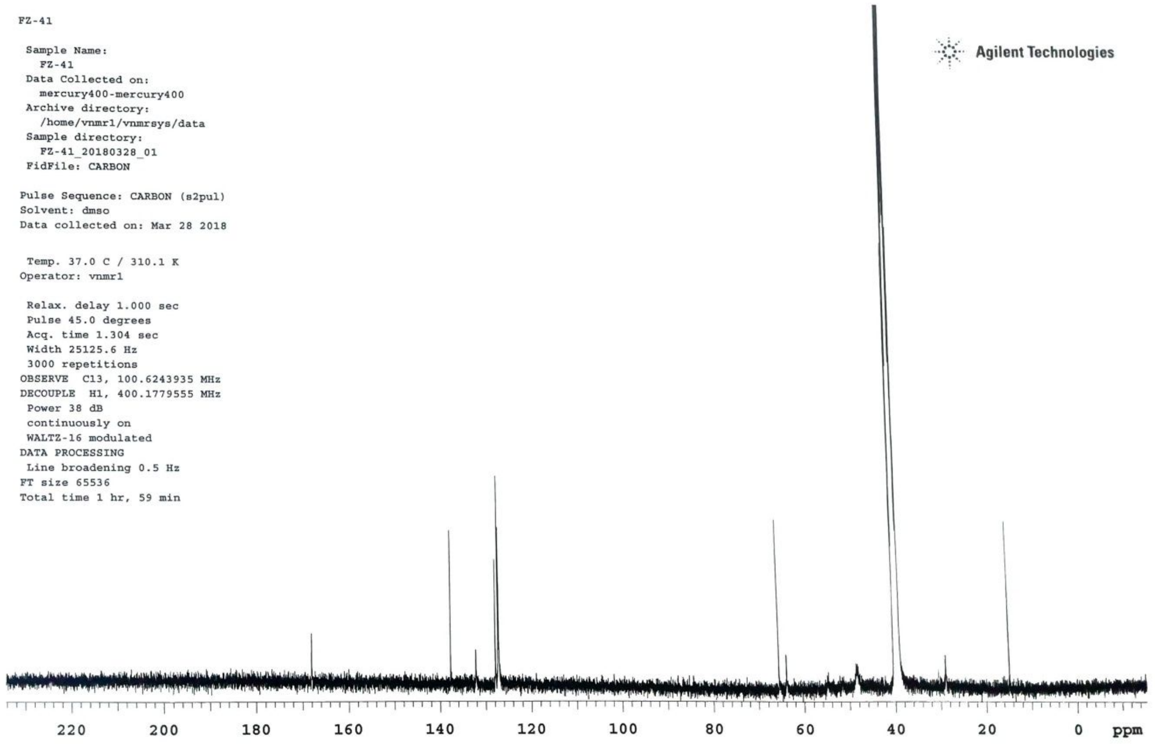

Figure S31. ^{13}C NMR spectrum of compound **15**.

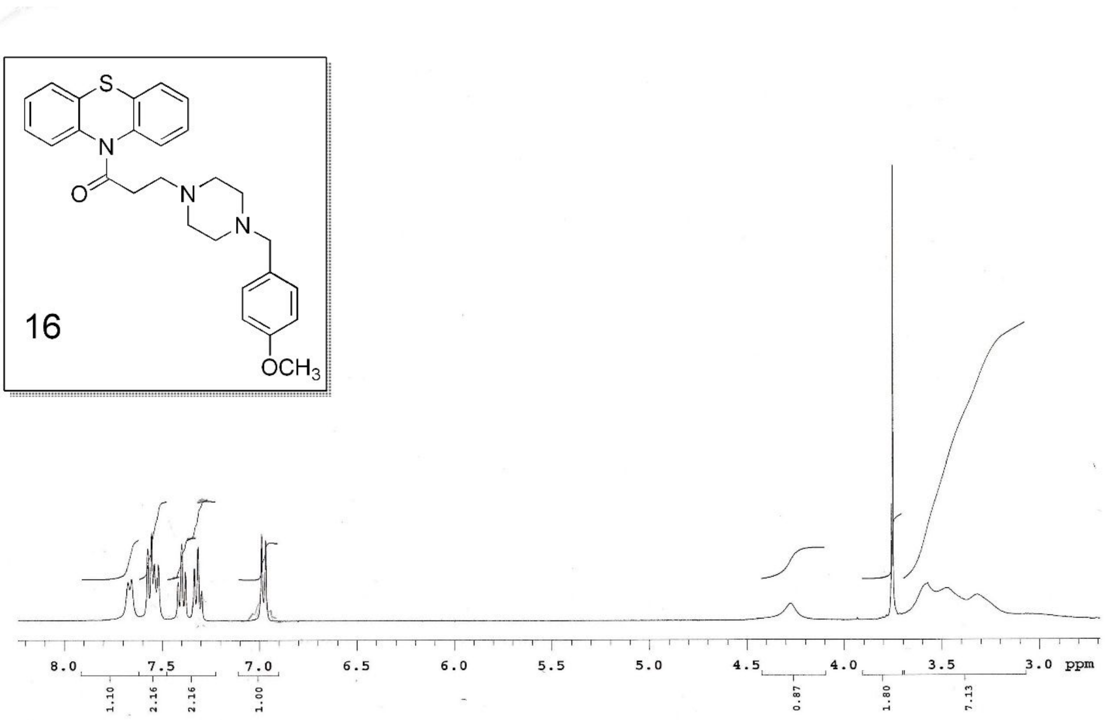

Figure S32. ^{1}H NMR spectrum of compound **16**.

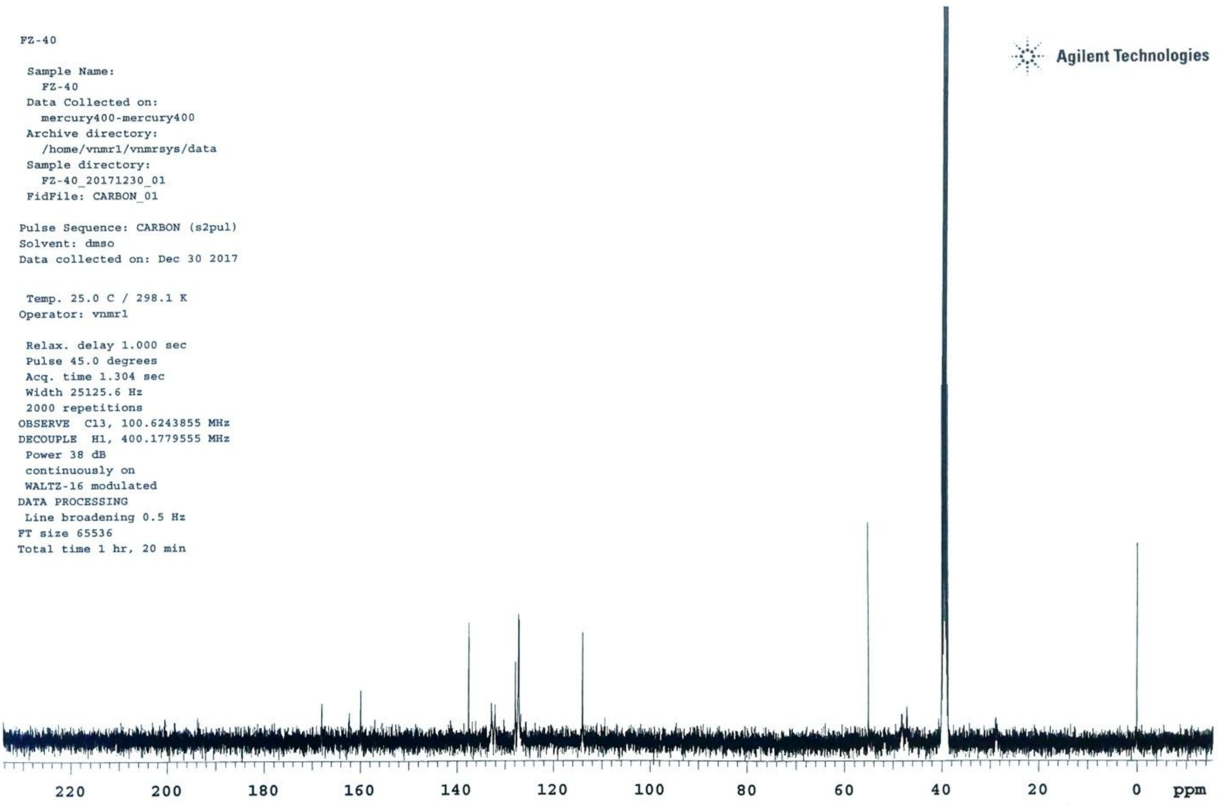

Figure S33. ^{13}C NMR spectrum of compound **16**.

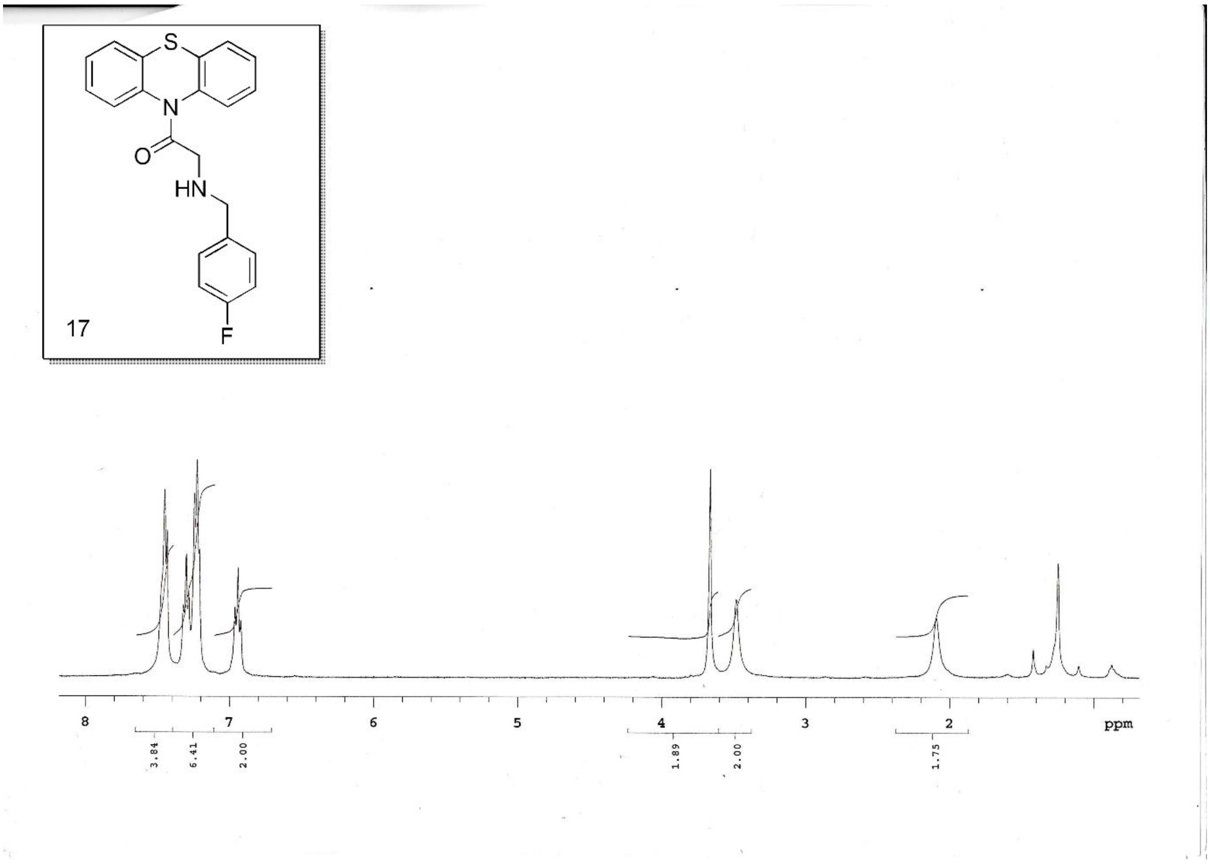

Figure S34. ^{1}H NMR spectrum of compound **17**.

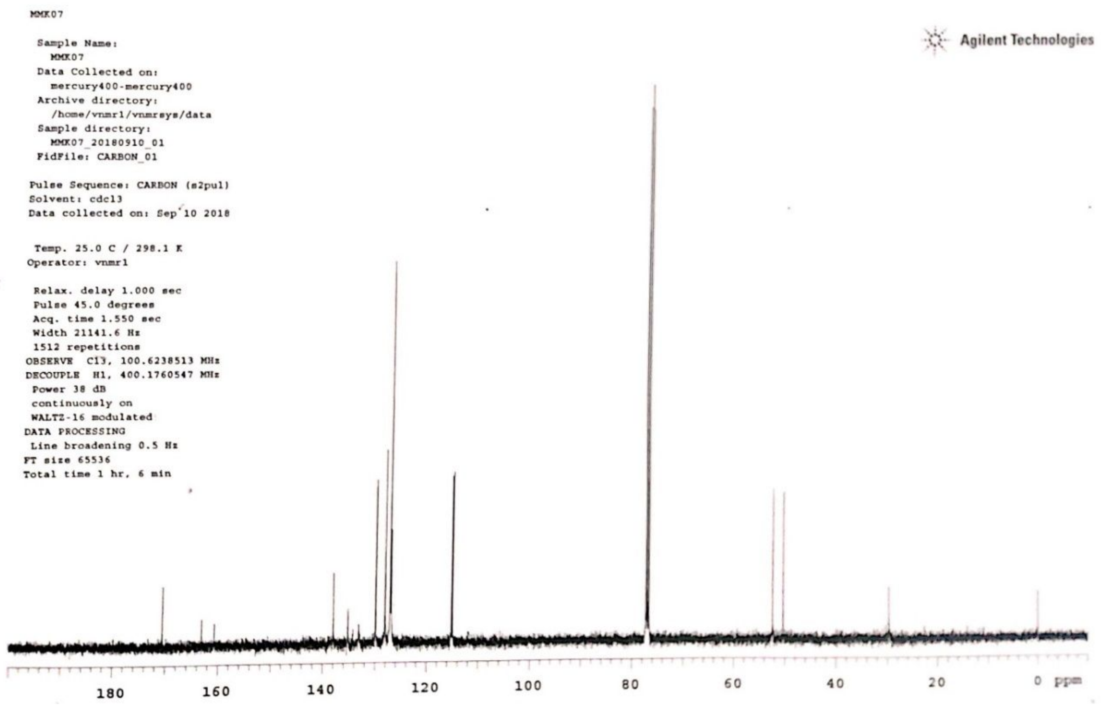

Figure S35. ^{13}C NMR spectrum of compound **17**.

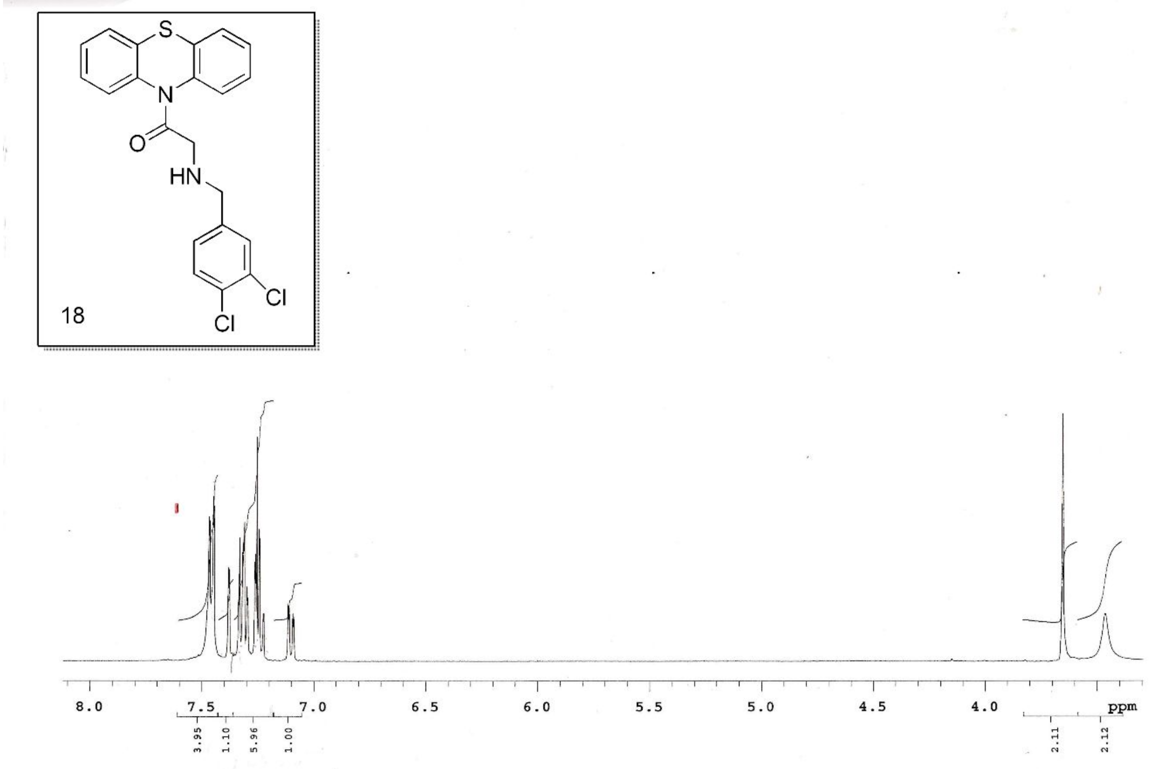

Figure S36. ^{1}H NMR spectrum of compound **18**.

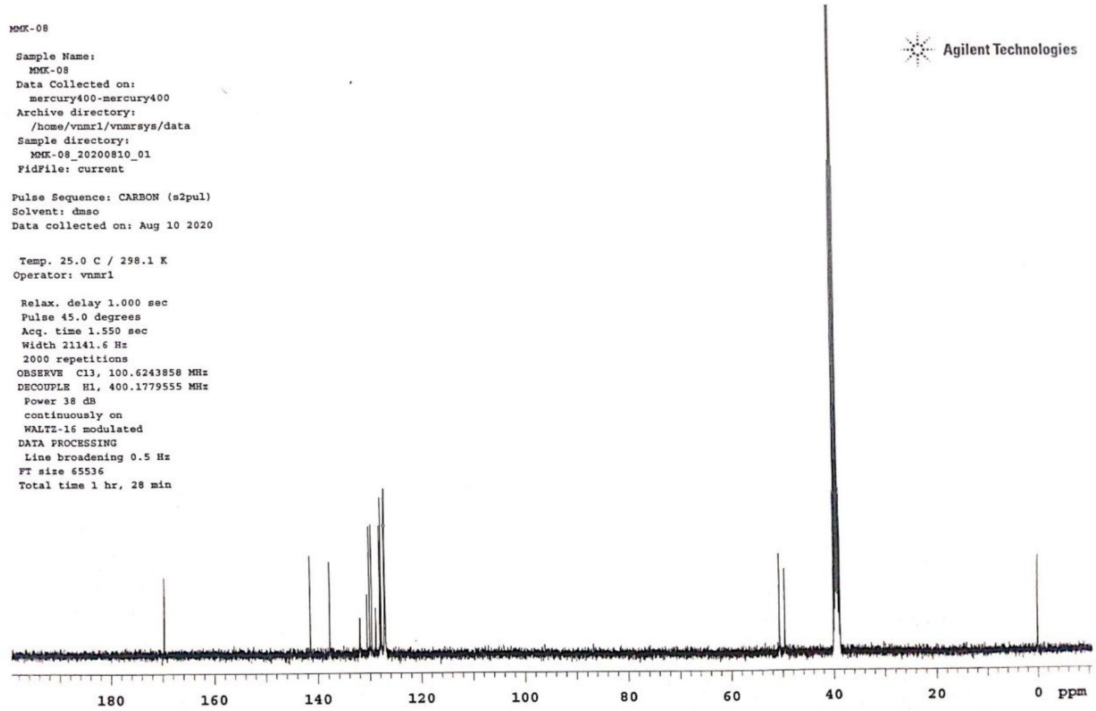

Figure S37. ^{13}C NMR spectrum of compound **18**.

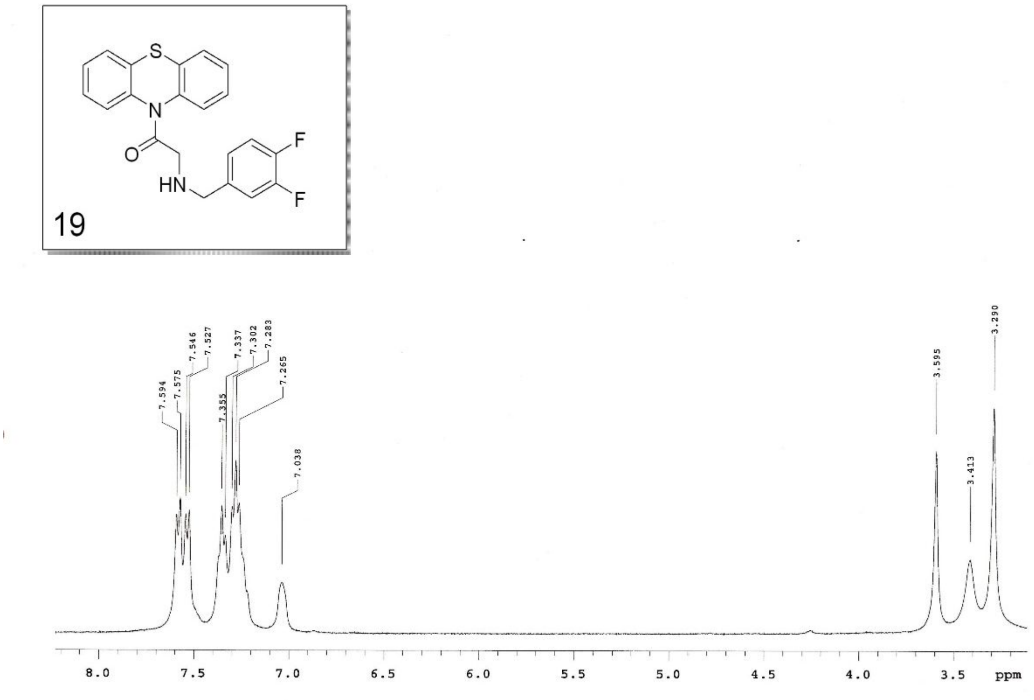

Figure S38. ^{1}H NMR spectrum of compound **19**.

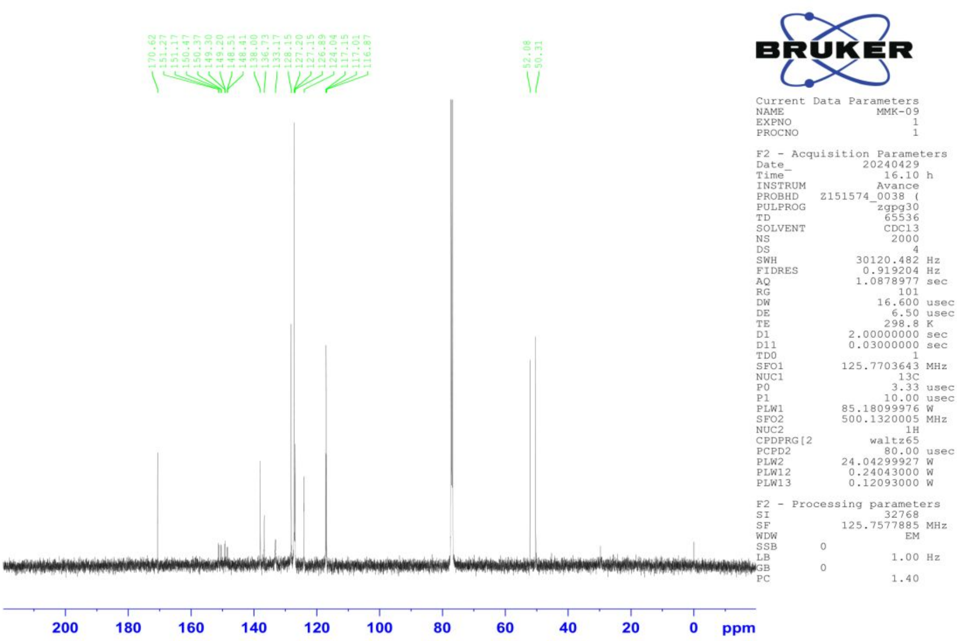

Figure S39. ^{13}C NMR spectrum of compound **19**.

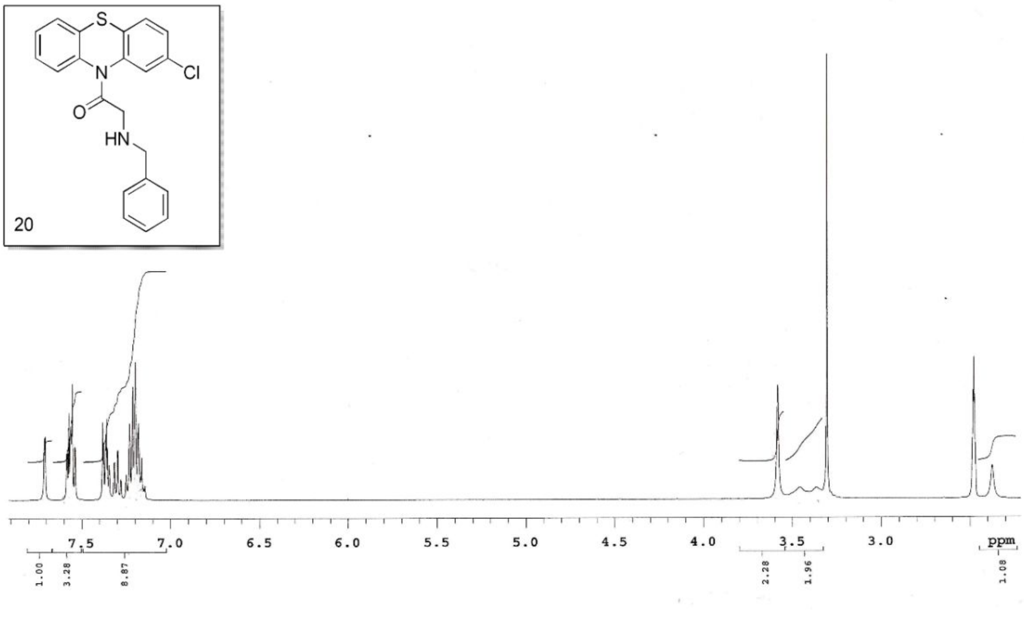

Figure S40. ^{1}H NMR spectrum of compound **20**.

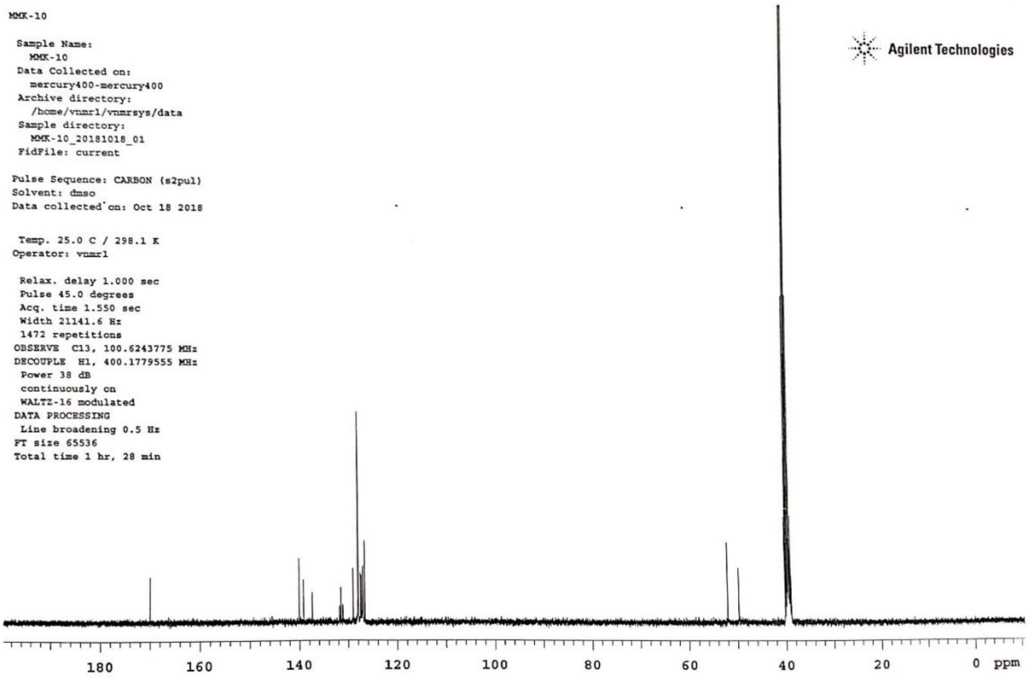

Figure S41. ^{13}C NMR spectrum of compound **20**.

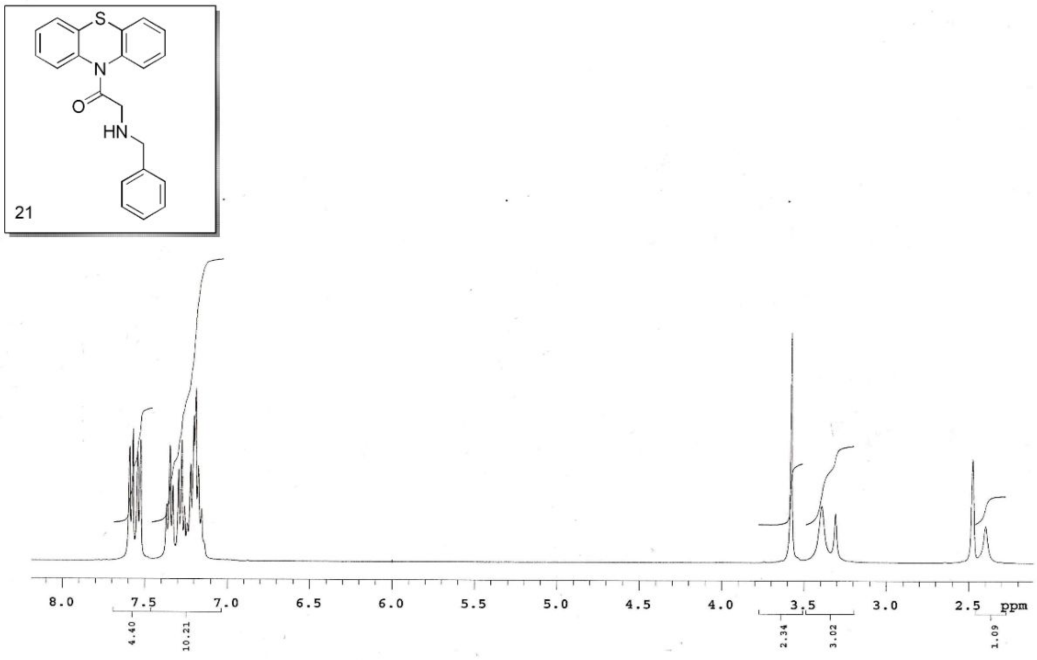

Figure S42. ^{1}H NMR spectrum of compound **21**.

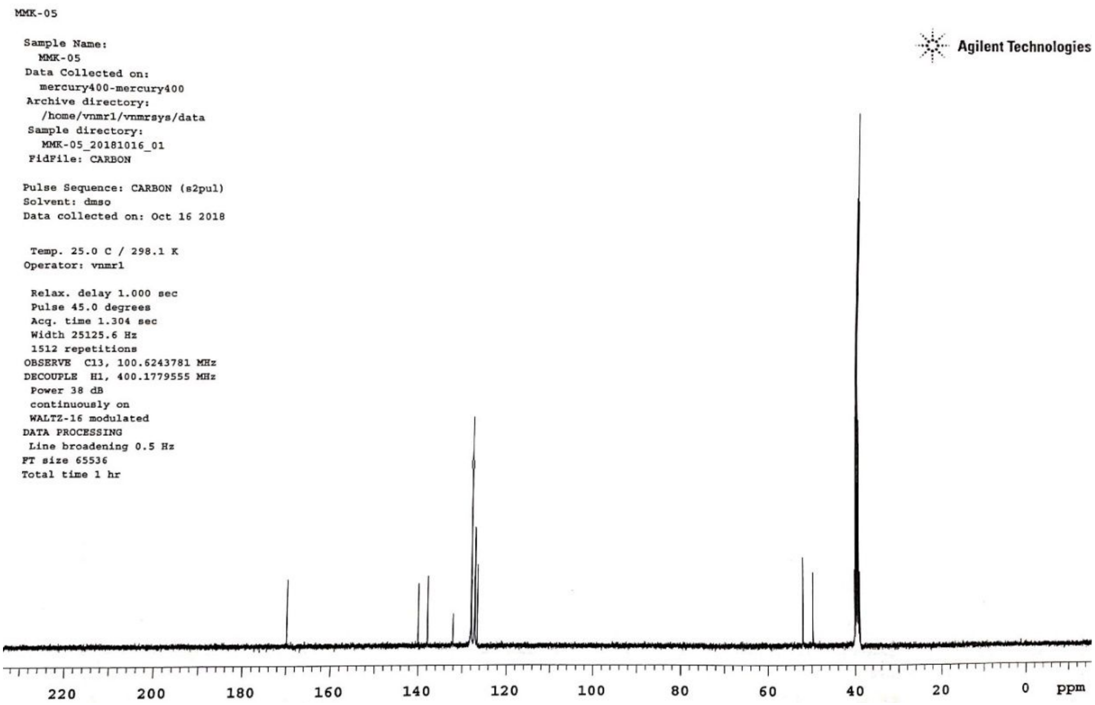

Figure S43. ^{13}C NMR spectrum of compound **21**.

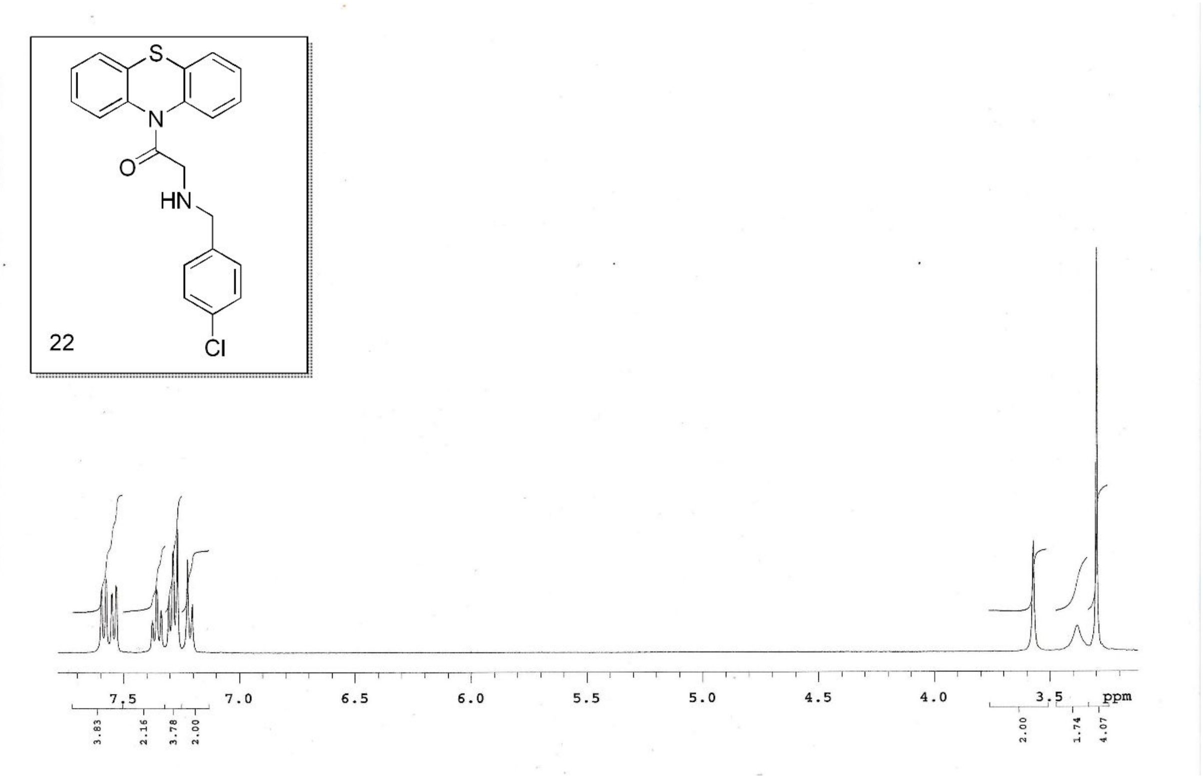

Figure S44. ^{1}H NMR spectrum of compound **22**.

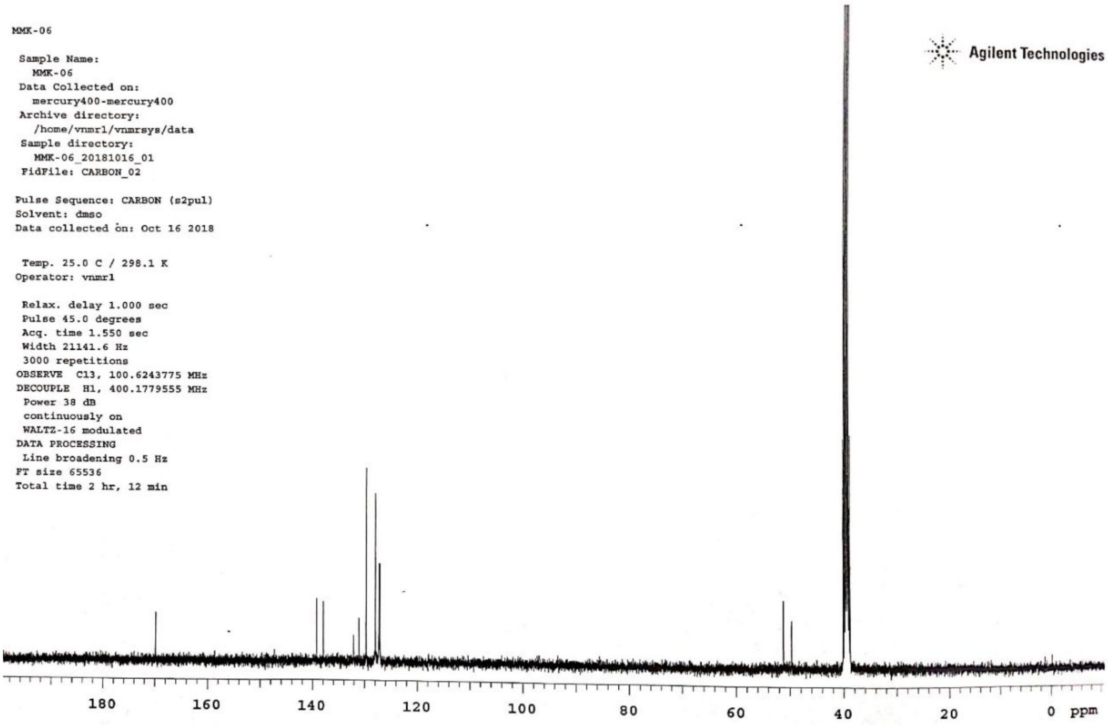

Figure S45. ^{13}C NMR spectrum of compound **22**.

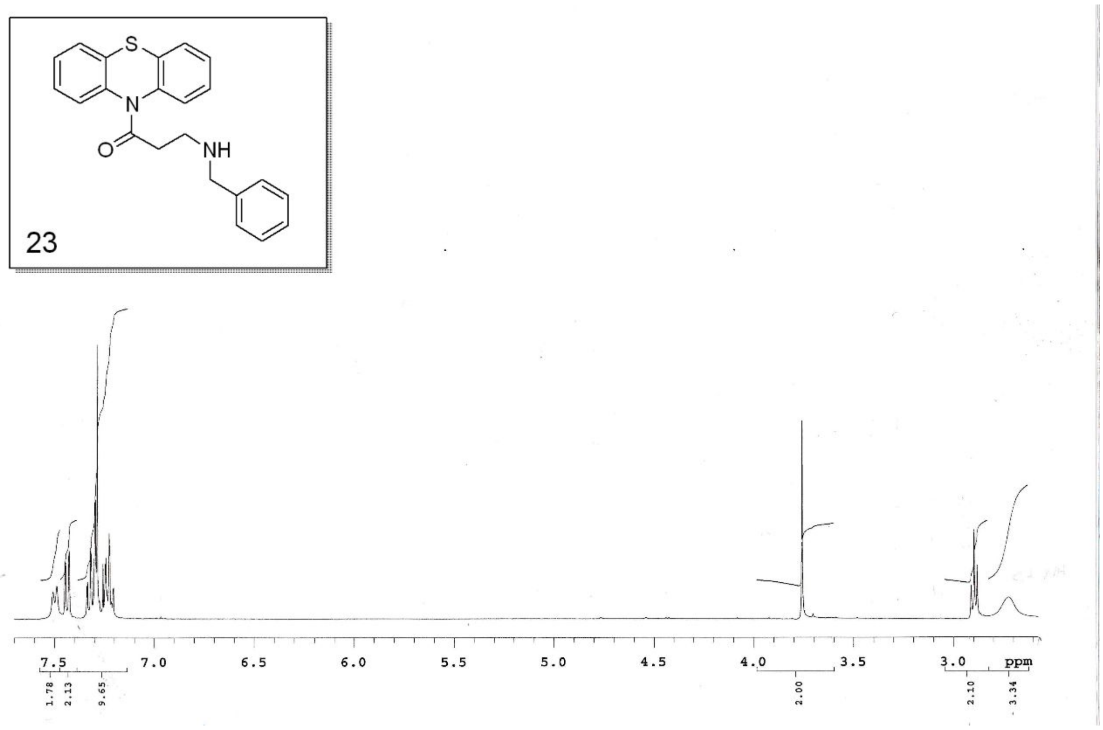

Figure S46. ^{1}H NMR spectrum of compound **23**.

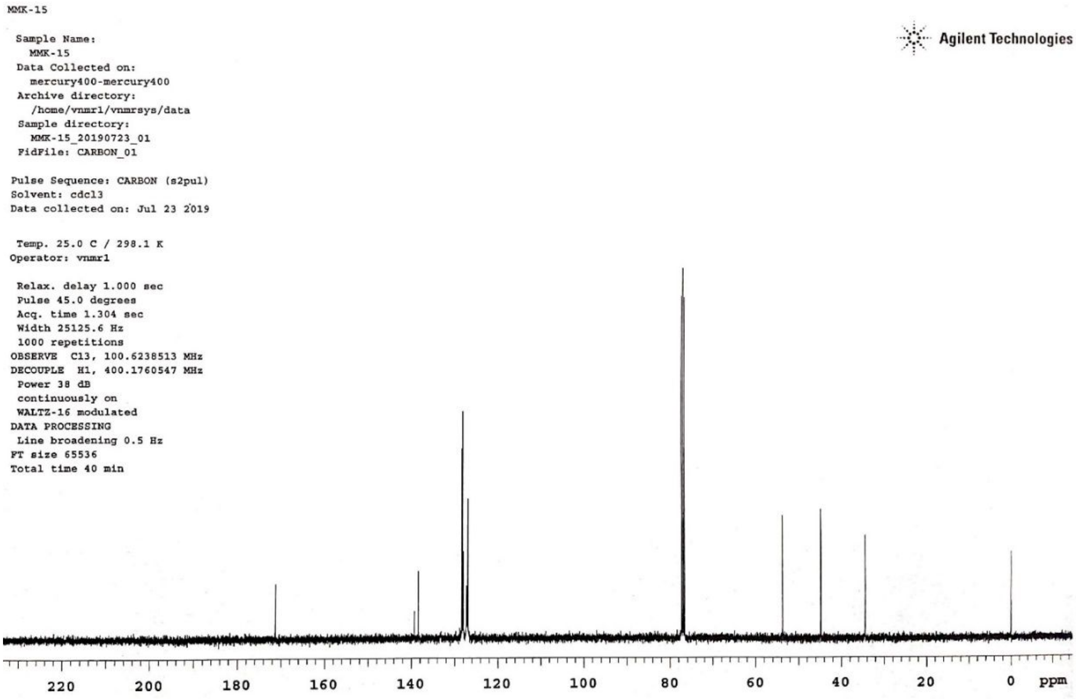

Figure S47. ^{13}C NMR spectrum of compound **23**.

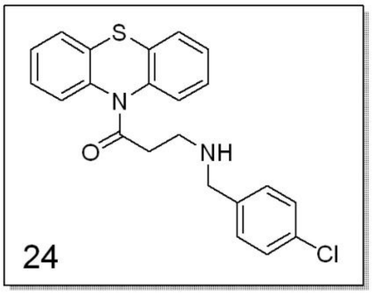

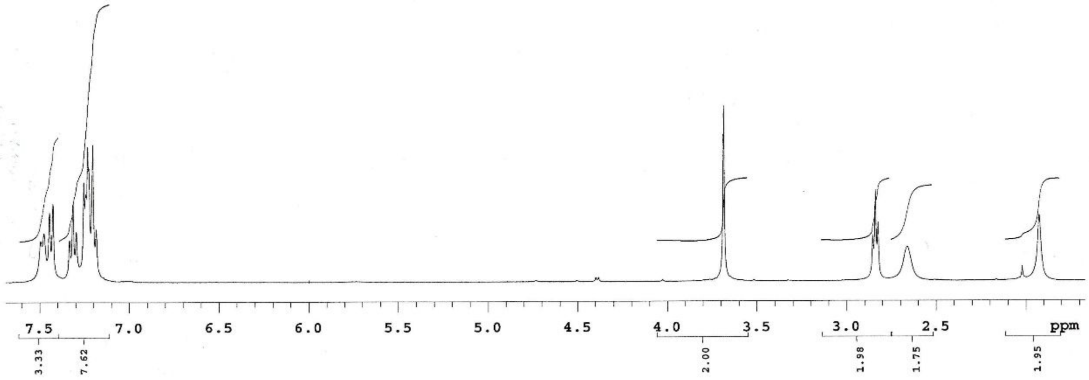

Figure S48. ^{1}H NMR spectrum of compound **24**.

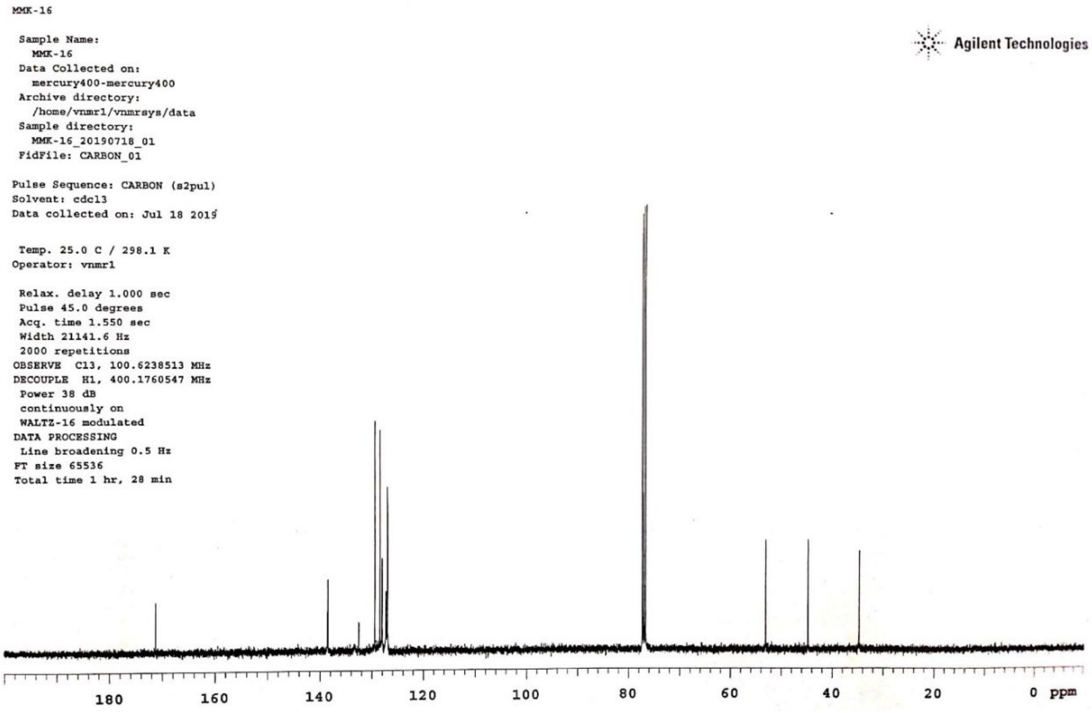

Figure S49. ^{13}C NMR spectrum of compound **24**.

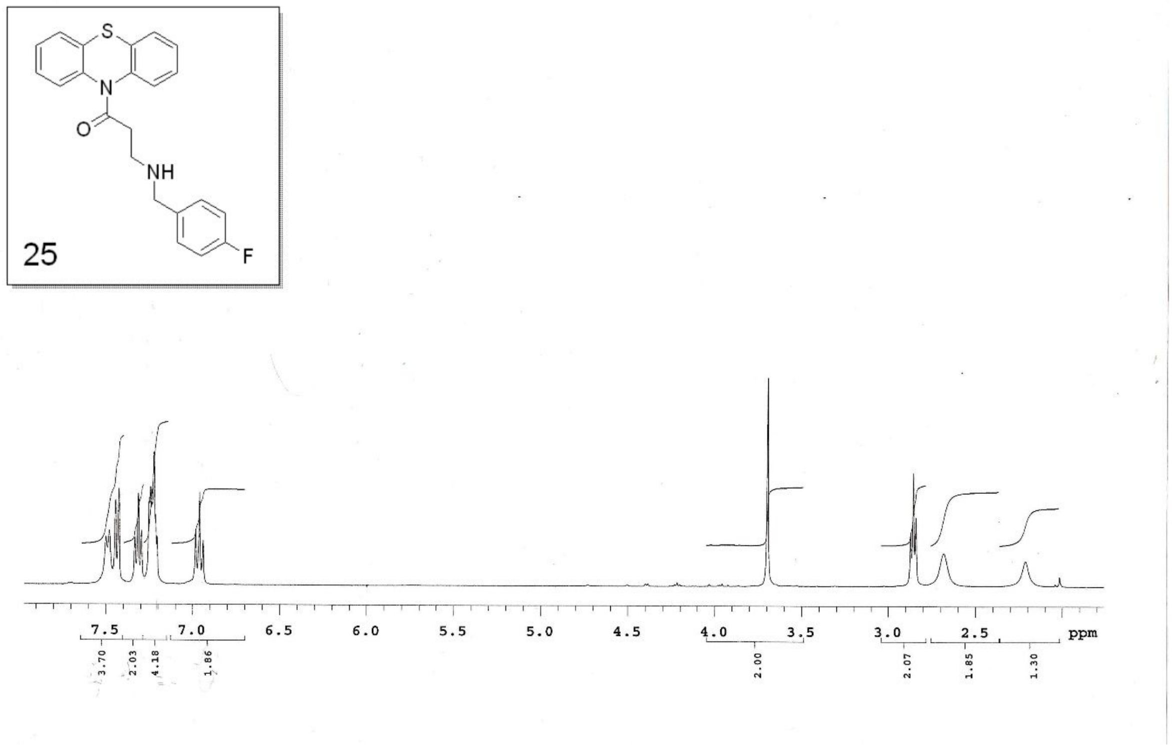

Figure S50. ^{1}H NMR spectrum of compound **25**.

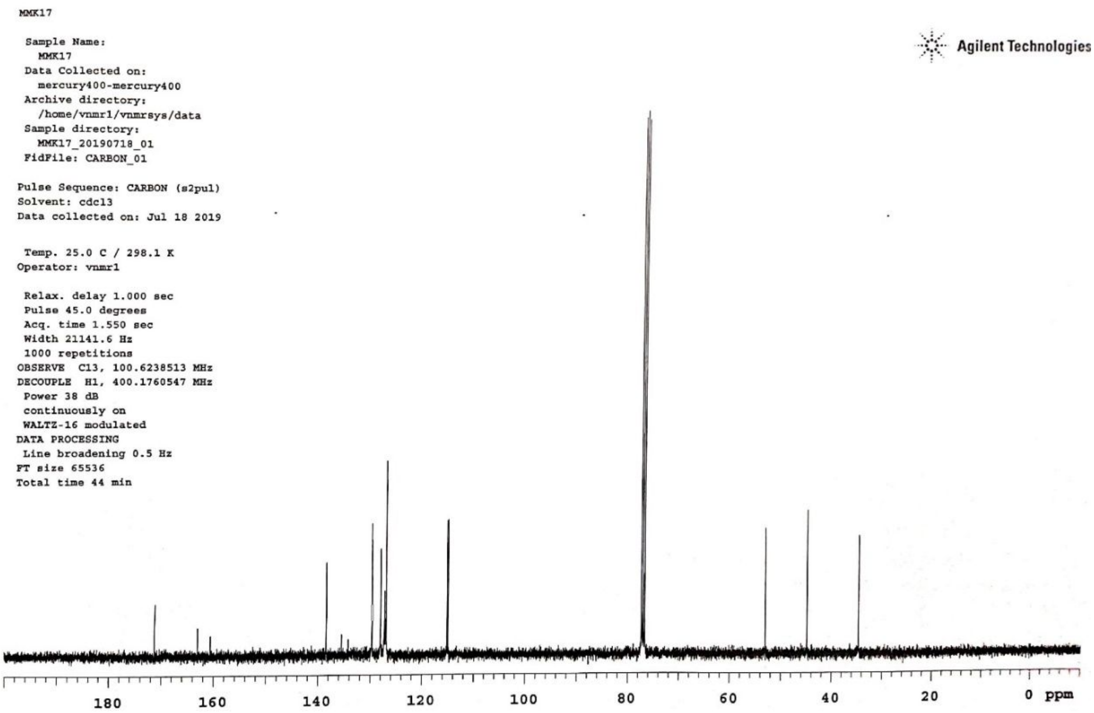

Figure S51. ^{13}C NMR spectrum of compound **25**.

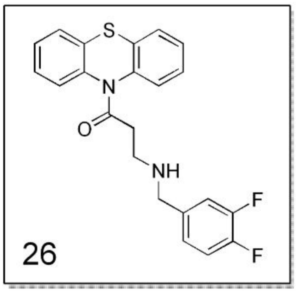

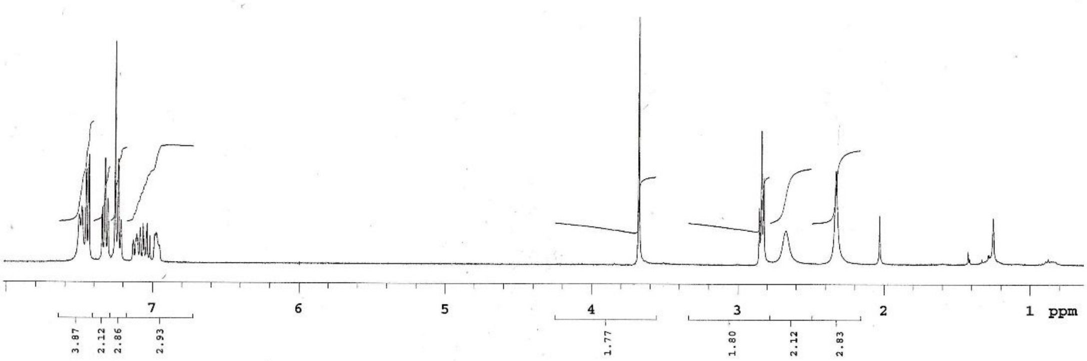

Figure S52. ^{1}H NMR spectrum of compound **26**.

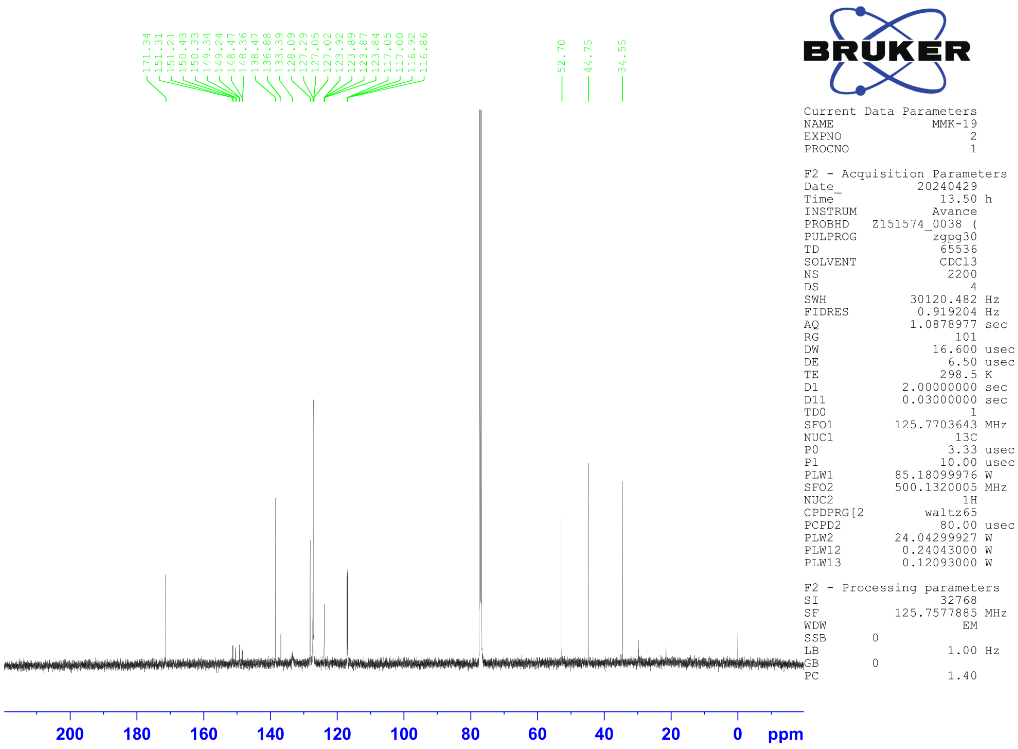

Figure S53. ^{13}C NMR spectrum of compound **26**.

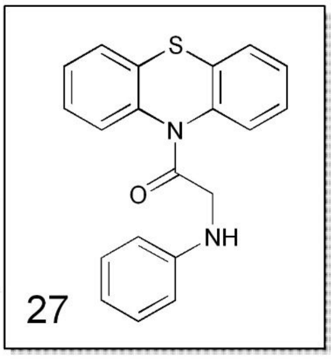

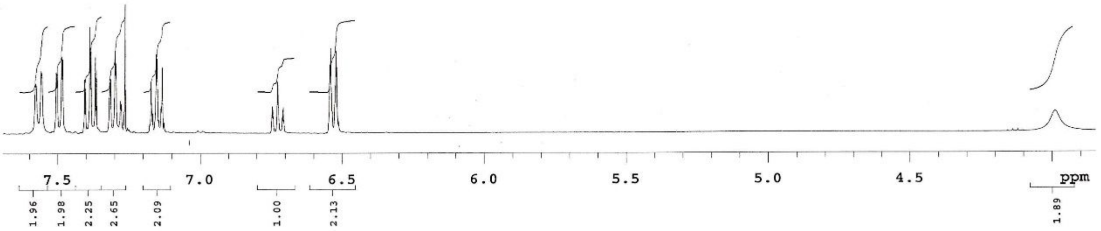

Figure S54. ^{1}H NMR spectrum of compound **27**.

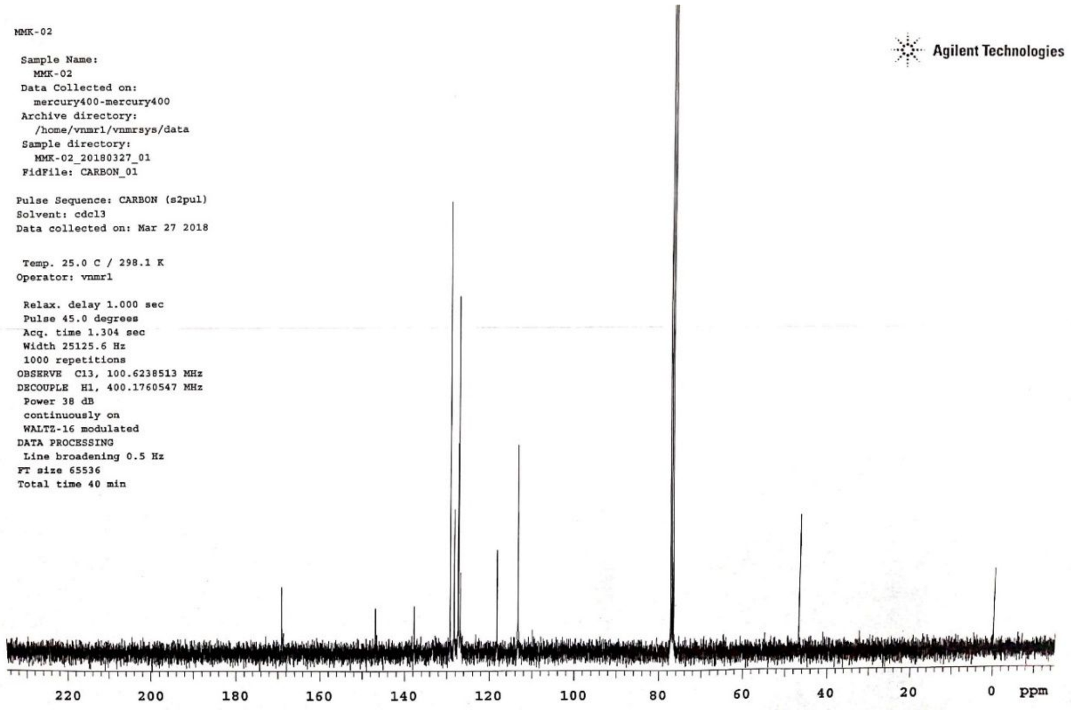

Figure S55. ^{13}C NMR spectrum of compound **27**.

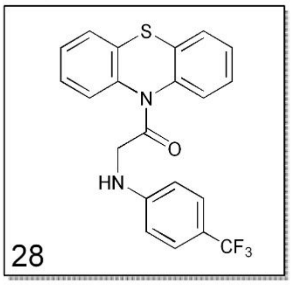

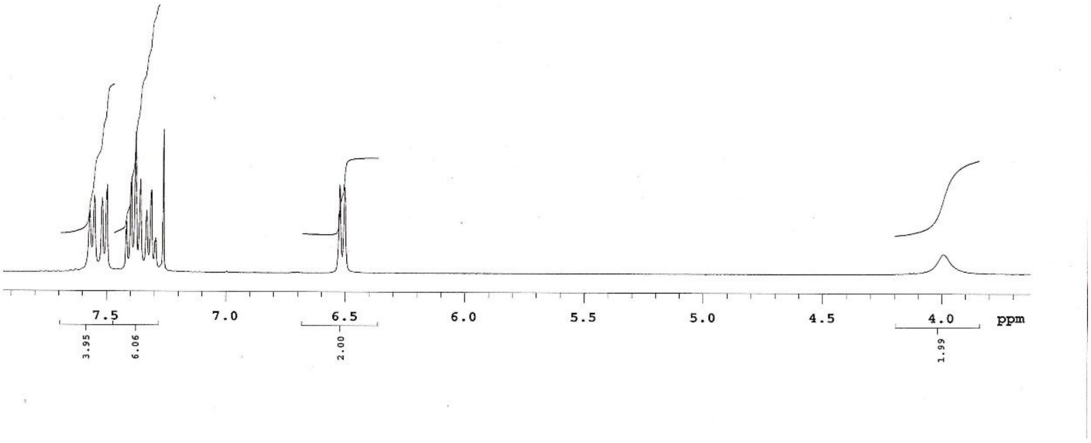

Figure S56. ^{1}H NMR spectrum of compound **28**.

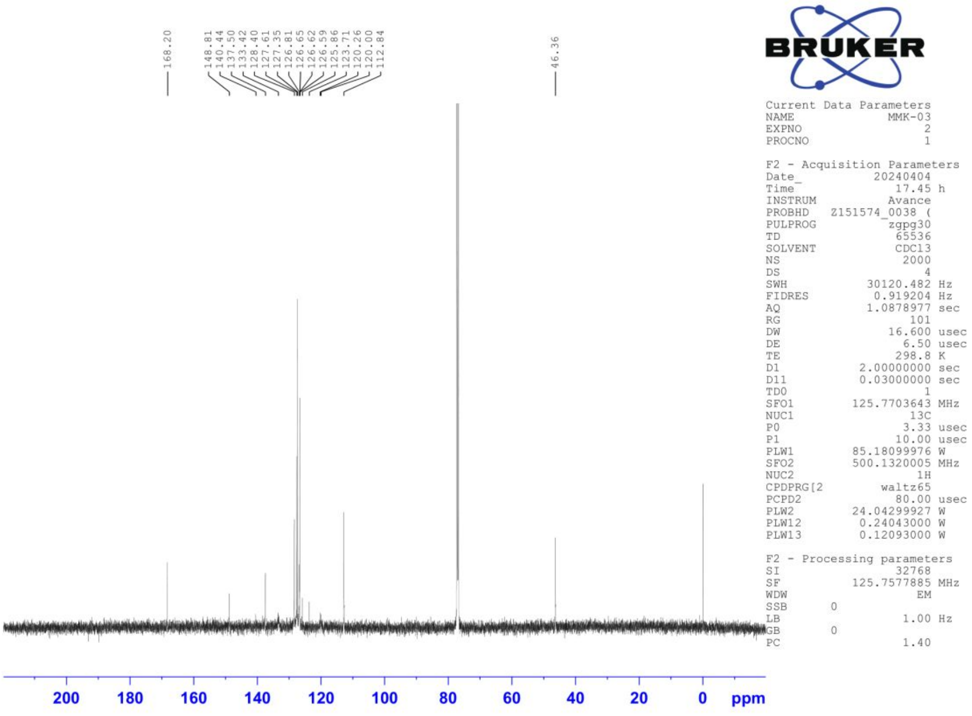

Figure S57. ^{13}C NMR spectrum of compound **28**.

Glide Scores of Phenothiazine Derivatives with AChE and BChE

Table S1. Glide scores of novel and intermediary phenothiazines with AChE (4BDT) and BChE (4BDS).

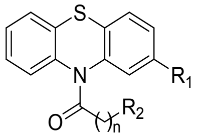

Compounds	n	R_1	R_2	Glide scores	
				AChE	BChE
Tacrine				nt*	-9.234
**HUW**				-14.76	nt
**1**	1	-H	-Cl	-8.216	-6.797
**2**	1	-Cl	-Cl	nb*	nb
**3**	1	$-SCH_3$	-Cl	-8.935	-6.560
**4**	2	-H	-Cl	-6.068	-6.643
**5**	2	-Cl	-Cl	-8.555	-6.729
**6**	1	-H	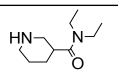	nb	nb
**7**	1	-H	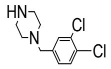	nb	nb
**8**	1	-H	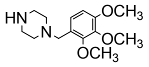	-6.976	nb
**9**	1	-Cl	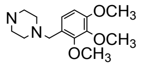	-8.069	nb
**10**	1	-Cl	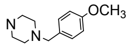	-7.739	nb
**11**	1	-Cl	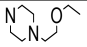	-6.803	nb
**12**	1	-Cl	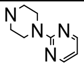	-5.590	nb
**13**	1	-H	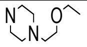	-6.964	nb
**14**	1	-H	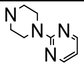	-6.787	nb
**15**	2	-H	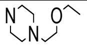	-7.219	nb
**16**	2	-H	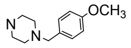	-5.906	nb
**17**	1	-H	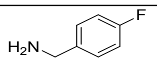	-8.504	-7.119
**18**	1	-H	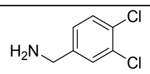	-6.059	-6.314

**19**	1	-H	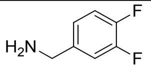	-7.681	-7.282
**20**	1	-Cl	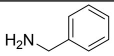	-6.579	nb
**21**	1	-H	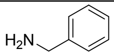	-8.260	-7.259
**22**	1	-H	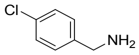	-7.851	-6.202
**23**	2	-H	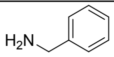	-8.795	-8.341
**24**	2	-H	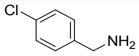	-9.205	-6.710
**25**	2	-H	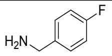	nb	nb
**26**	2	-H	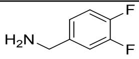	nb	nb
**27**	1	-H	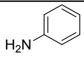	-8.272	-7.182
**28**	1	-H	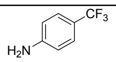	nb	-5.238

Table S2. Glide scores of commercial phenothiazines with AChE (4BDT) and BChE (4BDS).

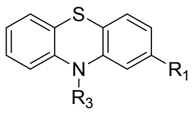

	Glide score		R_1	R_3
	**AChE**	**BChE**		
**Phenothiazine**	-6.814	-8.499	-H	-H
**Perphenazine**	-8.815	-6.285	-Cl	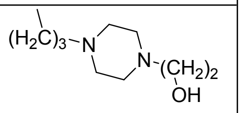
**Perchlorperazine**	-7.437	nb	-Cl	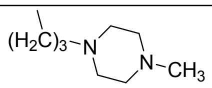
**Trifluoperazine**	-7.605	-6.177	$-CF_3$	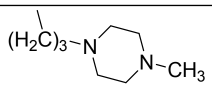
**Fluphenazine**	nb	nb	$-CF_3$	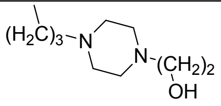
**Chlorpromazine**	-8.103	nb	-Cl	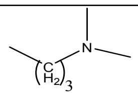
**Trifluopromazine**	-6.443	-7.105	$-CF_3$	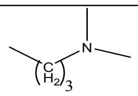
**Thioridazine**	-7.585	-7.452	$-SCH_3$	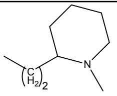

Pharmacophore Screening Based on HPRR_3 Pharmacophore Hypothesis

Table S3. Pharmacophore screening results of the compounds with the highest fitness scores.

Compound	Molecular formula	PhaseScreenScore
**9**	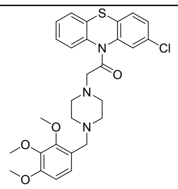	2.149
**10**	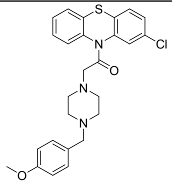	1.876
**8**	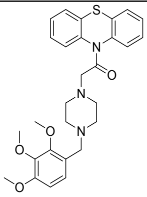	1.575
**7**	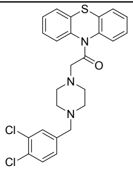	0.649
